# Synthesis, characterization, crystallography insights, and larvicidal activity of 4-substituted-8-(trifluoromethyl)quinoline derivatives against *Anopheles arabiensis*

**DOI:** 10.1039/d6ra05130h

**Published:** 2026-08-27

**Authors:** Sukumar Kotyan, Banavase N. Lakshminarayana, Lina A. Dahabiyeh, Karthik Kumara, Shankaranahalli N. Chandana, Arya Viswanath M, Raquel M. Gleiser, Abdulaziz K. Al Mouslem, Nefisath Pandikatte, Pran Kishore Deb, Ravikumar Jimmidi, Katharigatta N. Venugopala, Jagadeesh Prasad Dasappa

**Affiliations:** a Department of Chemistry, Mangalore University Mangalagangotri Karnataka 574199 India sukumarkotyan8793@gmail.com jprasad2003@gmail.com; b Research Center, Department of Physics, Adichunchanagiri Institute of Technology, Affiliated to Visvesvaraya Technological University Jyothinagara Chikkamagaluru Karnataka 577102 India bnlphysics@gmail.com; c Department of Pharmaceutical Sciences, School of Pharmacy, The University of Jordan Amman Jordan L.Dahabiyeh@ju.edu.jo; d Department of Physics & BSN Centre for Nano-Materials and Displays, B.M.S College of Engineering Bull Temple Road Bangalore 560019 India kk.phy21@gmail.com; e Department of Physics, Rajeev Institute of Technology Hassan Karnataka 573201 India snc.rit@gmail.com aryaviswanath@bmsce.ac.in; f CREAN-IMBIV (UNC-CONICET), Universidad Nacional de Cordoba Av. Valparaiso s.n., and FCEFyN, Av. V. Sarsfield 299 Cordoba 5000 Argentina raquel.gleiser@unc.edu.ar; g Department of Pharmaceutical Sciences, College of Clinical Pharmacy, King Faisal University Al-Ahsa 31982 Saudi Arabia aalmoslem@kfu.edu.sa; h Department of PG Studies and Research in Chemistry, Sri Dharmasthala Manjunatheshwara College (Autonomous) Ujire Karnataka 574240 India nafeesath@sdmcujire.in; i Department of Pharmaceutical Sciences and Technology, Birla Institute of Technology (BIT) Mesra 835215 Jharkhand India prankishoredeb@bitmesra.ac.in; j Center for Drug Discovery, Department of Pathology & Immunology, Baylor College of Medicine Houston TX 77030 USA RAVIKUMAR.JIMMIDI@bcm.edu; k Department of Biotechnology and Food Science, Faculty of Applied Sciences, Durban University of Technology Durban 4001 South Africa kvenugopala@kfu.edu.sa

## Abstract

A new series of 8-trifluoromethyl quinoline derivatives bearing substituted saturated amines has been synthesized by standard conventional reflux methods. The structures of the novel saturated amine derivatives 4a–4h were characterized by ^1^H and ^13^C-NMR and mass spectrometry. Among the designed compounds 4a–4h, compound 4a was subjected to the single-crystal X-ray diffraction technique (SCXRD). According to the SCXRD analysis, the compound crystallized in a monoclinic lattice structure with the space group *P*2_1_/*n*. The crystal structure is stabilized through intermolecular C–H⋯O interactions. Hirshfeld surface analysis is performed to validate the intermolecular interactions of compound 4a. Density functional theory (DFT) calculations are performed to study the optimized structure, molecular geometry, and molecular electrostatic potential (MEP) of compound 4a using the B3LYP/6–311++G(d,p) level of theory in the gas phase. We evaluated the title compounds for larvicidal activity using Temephos as the standard reference against *Anopheles arabiensis.* Compound 4b exhibited the highest larval mortality, at 97%, after 48 h of exposure. The synthesized compounds were generally non-toxic to normal fibroblasts. To further validate the biological activity, molecular docking studies were performed on the designed title compounds 4a–4h against five malaria vectors (PDB IDs: 1ZP4, 2CH2, 4JBV, 5V13, and 6ARY). In *silico* ADMET profiles were also screened to evaluate the drug-likeness and toxicity of the tested compounds.

## Abstract 2

Fluorinated quinoline derivatives incorporating saturated amines showed potent larvicidal activity against *Anopheles arabiensis*. Compound 4b emerged as the most active derivative, supported by crystallographic and computational investigations.

## Graphical abstract

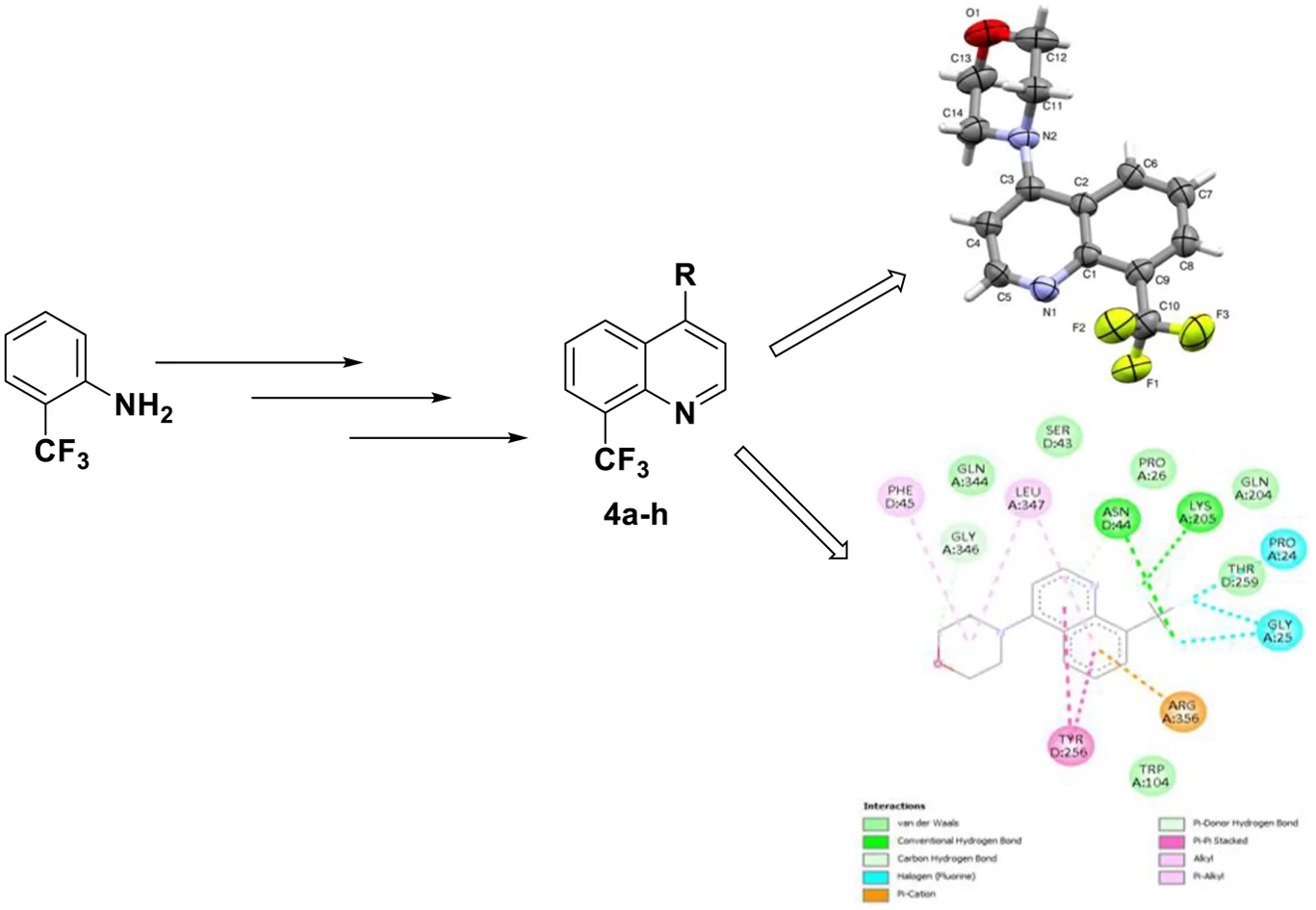

## 1. Introduction

Quinoline is a heterocyclic aromatic compound that plays a crucial role in medicinal chemistry due to its broad pharmacological activities.^1^ Quinolines, which contain a nitrogen atom in their structure, exhibit a wide range of important pharmacological activities, including antimalarial,^2^ antimicrobial,^3^ antimycobacterial,^4^ antidepressant,^5^ antiviral,^6^ antiproliferative,^7^ antimalarial,^8^ leishmanicidal,^9^ anticonvulsant,^10,11^ antihypertensive,^12^ and anti-inflammatory effects.^13^ They are widely used in the treatment of urinary tract,^14^ respiratory,^15^ and bone joint infections,^16^ as well as pneumonia,^17^ prostatitis,^18^ acute bronchitis,^19^ and sexually transmitted diseases.^20^ Quinine and quinidine, both obtained from the bark of the cinchona plant, have been used to treat palpitations and fevers, and as antimalarial^21^ and antiarrhythmic agents,^22^ respectively. Structural modifications of quinoline, such as the introduction of trifluoromethyl and saturated amine groups, significantly enhance its pharmacological activity and physicochemical properties.

The incorporation of the trifluoromethyl group in quinoline scaffolds is widely employed in medicinal chemistry because it enhances lipophilicity,^23^ metabolic stability,^24^ bioavailability,^25^ and target-binding affinity.^26^ Trifluoromethyl quinolines have shown promising results in drug discovery, particularly as anticancer agents,^27,28^ antimicrobial agents,^29^ and anti-inflammatory agents.^30^ Compounds containing a quinoline moiety are well known for their broad spectrum of biological activities.^31^ Saturated amines, including piperidine, pyrrolidine, and morpholine derivatives, are commonly introduced into quinoline structures to improve solubility, enhance receptor interactions, and modify pharmacokinetic properties.^32,33^ These derivatives have demonstrated enhanced potency in various therapeutic applications, including kinase inhibitors,^34^ antimalarial agents,^35^ and central nervous system (CNS) drugs.^36^

**Fig. 1:**
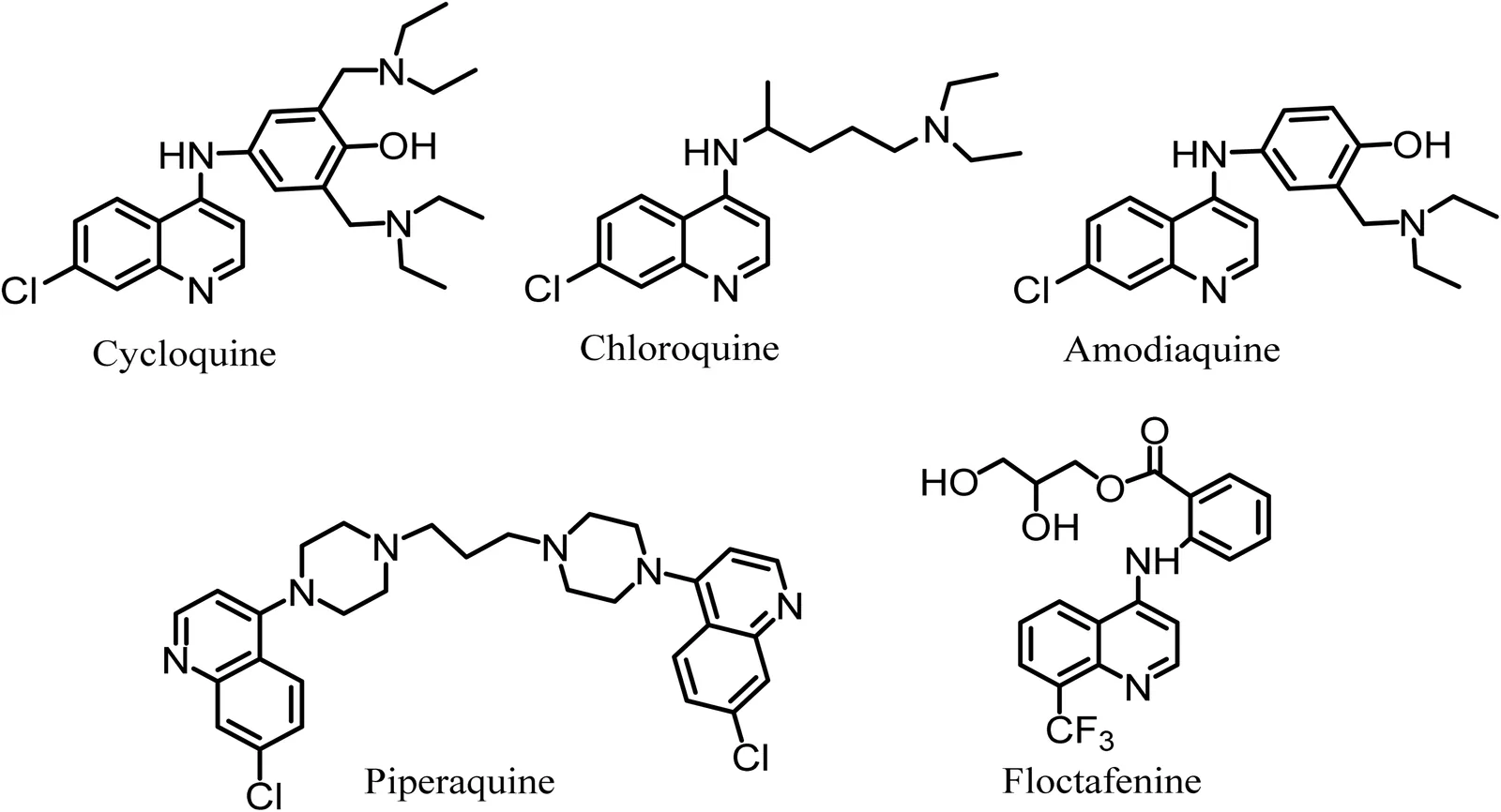
Some quinoline drugs bearing aliphatic and aromatic amines have therapeutic applications.

The chemical structure of representative quinoline-based drugs with established therapeutic activity is shown in Fig. 1. The CF_3_ group is highly electronegative and withdraws electron density from the quinoline ring system through inductive effects. This makes the ring more electron-deficient. The presence of the fluorinated group increases the molecule's lipophilicity (fat solubility), a crucial factor in medicinal chemistry for improving cell membrane permeability and bioavailability.

The strong carbon-fluorine bonds confer increased thermal and chemical stability to the quinoline ring system, making it more resistant to metabolic degradation in biological systems. Introduction of the CF_3_ group into the organic molecule immensely increases pharmacological activity and lipophilicity.^37,38^ Hence, our strategy is to replace the chlorine atom at the 4th position by attaching various alkyl amines and other heterocyclic ring systems. In the present work, we have taken 4-chloro-8-trifluoromethyl quinoline as the starting material. Fluorinated compounds can exhibit dramatically improved potency compared to their non-fluorinated analogs.^39^ Most widely used antimalarial drugs are chloroquine and amodiaquine, which belong to the class of 4-aminoquinoline, and the biological activity of these compounds has been attributed to the presence of electron-releasing alkyl amine/side chain/saturated amine/arylamine attached at the 4th position of quinoline.

Although several quinoline derivatives containing piperidine, piperazine, and morpholine substituents have been reported for diverse biological activities, including antimicrobial,^40^ antimalarial, and anticancer applications,^41^ relatively few studies have investigated 8-(trifluoromethyl)quinoline derivatives as larvicidal agents against *Anopheles arabiensis*. The present compounds differ from previously reported analogues by combining an electron-withdrawing CF_3_ substituent at the 8-position with structurally diverse saturated cyclic amines at the 4-position of the quinoline nucleus. This dual structural modification was designed to improve lipophilicity, metabolic stability, and target interactions while enabling systematic evaluation of how different cyclic amines influence larvicidal activity and structure–activity relationships.

In continuation of our ongoing drug discovery efforts focused on larvicidal activity^42–44^ and the evaluation of heterocyclic compounds for polymorphic properties,^45–47^ we herein report the design and synthesis of 4-substituted-8-(trifluoromethyl)quinoline derivatives targeting *Anopheles arabiensis*.

## 2. Experimental

### 2.1. Chemistry

The reactants, such as 4-chloro-8-trifluoromethyl quinoline (TFMQ) 3 and saturated amines, were procured from Sigma-Aldrich (India) and Alfa Aesar (U.K.) and used as received. The melting points of target compounds 8-trifluoromethyl quinoline derivatives bearing substituted saturated amines 4a–4h were determined by the open capillary method and were uncorrected. The purity of the product was monitored by TLC on Merck silica gel 60 F254-coated alumina plates using chloroform/methanol as the mobile phase, with spots visualized by iodine vapor and ultraviolet light. The ^1^H and ^13^C NMR spectra were recorded on a Bruker AMX-400 (400 MHz) spectrometer using CDCl_3_ and DMSO as solvents and TMS as the internal standard. The splitting patterns are designated as follows: s, singlet; d, doublet; t, triplet; m, multiplet. The mass spectrum was recorded on a PerkinElmer 018444Y, triple quadrupole LC-MS spectrometer. The molecular ion peak (*m*/*z*) was equivalent to its calculated molecular weight.

### 2.2 General procedure for the synthesis of 8-trifluoromethyl quinoline derivatives bearing substituted saturated amines (4a–4h)

A mixture of 4-chloro-8-(trifluoromethyl) quinoline 3 (1.0 eq) and saturated amines (3.0 eq) was refluxed in ethanol in the presence of triethylamine (2.0 eq) on a water bath for 22 hours. The progress of the reaction was monitored by TLC at appropriate time intervals. After completion of the reaction, the solution was poured onto crushed ice; the solid thus separated was filtered, washed with ice-cold water, and dried. Finally, the products 4a–4h thus obtained were recrystallized from methanol.

#### 2.2.1 4-(8-(Trifluoromethyl)quinolin-4-yl)morpholine (4a)

White solid; yield: 80%; m.p.: 193–198 °C; ^1^H-NMR (400 MHz, CDCl_3_) *δ* = 8.92 (d, 1H, *J* = 4.8 Hz, TFMQ), 8.27 (d, 1H, *J* = 8 Hz, TFMQ), 8.05 (d, 1H, *J* = 7.2 Hz, TFMQ), 7.57 (m, 1H, TFMQ), 6.98 (d, 1H, *J* = 4.8 Hz, TFMQ), 4.03–4.01 (m, 4H, O–(CH_2_)_2_), 3.26–3.23 (m, 4H, N–(CH_2_)_2_); ^13^C-NMR (100 MHz CDCl_3_) *δ* = 156.9, 151.7, 146.2, 128.3, 128.1, 127.8, 127.7, 125.5, 124.0, 123.9, 122.8, 109.5, 66.8, 52.7. MS: *m*/*z* = 282.9 (M^+^).

#### 2.2.2 4-(4-Phenylpiperazin-1-yl)-8-(trifluoromethyl)quinoline (4b)

Brown solid; yield: 70%; m.p.: 172–177 °C; ^1^H-NMR (400 MHz, CDCl_3_) *δ* = 8.93 (d, 1H, *J* = 4.8 Hz, TFMQ), 8.31 (d, 1H, *J* = 8.4 Hz, TFMQ), 8.06 (d, 1H, *J* = 7.6 Hz, TFMQ), 7.56 (m, 1H, TFMQ), 7.36–7.27 (m, 2H, Ar–H), 7.05–7.02 (m, 3H, Ar–H), 6.95 (m, 1H, TFMQ), 3.50–3.49 (m, 4H, N–(CH_2_)_2_), 3.42–3.40 (m, 4H, *J* = 4.4 Hz, N–(CH_2_)_2_); ^13^C-NMR (100 MHz CDCl_3_) *δ* = 157.0, 151.7, 151.0, 146.3, 129.3, 128.2, 127.7, 124.2, 123.9, 120.4, 116.4, 109.7, 52.4, 49.4. MS: *m*/*z* = 358.0 (M^+^).

#### 2.2.3 4-(4-Ethylpiperazin-1-yl)-8-(trifluoromethyl)quinoline (4c)

Off white solid; yield: 72%; m.p.: 135–140 °C; ^1^H-NMR (400 MHz, CDCl_3_) *δ* = 8.88 (d, 1H, *J* = 4.8 Hz, TFMQ), 8.24 (d, 1H, *J* = 8.0 Hz, TFMQ), 8.02 (d, 1H, *J* = 7.2 Hz, TFMQ), 7.52 (t, 1H, *J* = 8.0 Hz, TFMQ), 6.96 (d, 1H, *J* = 4.8 Hz, TFMQ), 3.27 (br, t, 4H, *J* = 4.4 Hz, N–(CH_2_)_2_), 2.76 (br, 4H, N–(CH_2_)_2_), 2.56 (q, 2H, *J* = 6.8, 7.2 Hz, –CH_2_), 1.17 (t, 3H, *J* = 6.8, 7.6 Hz, –CH_3_); ^13^C-NMR (100 MHz DMSO-*d*6) *δ* = 156.5, 151.6, 145.3, 128.9, 127.7, 126.6, 126.3, 125.6, 124.1, 123.3, 122.9, 109.8, 52.0, 51.8, 51.5, 11.8. MS: *m*/*z* = 310.0 (M^+^).

#### 2.2.4 4-(4-Methylpiperazin-1-yl)-8-(trifluoromethyl)quinoline (4d)

White solid; yield: 82%; m.p.: 152–157 °C; ^1^H-NMR (400 MHz, DMSO-*d*_6_) *δ* = 8.88 (d, 1H, *J* = 4.8 Hz, TFMQ), 8.24 (d, 1H, *J* = 8.0 Hz, TFMQ), 8.02 (d, 1H, *J* = 7.2 Hz, TFMQ), 7.52 (t, 1H, *J* = 8.0 Hz, TFMQ), 7.12 (d, 1H, *J* = 4.4 Hz, TFMQ), 3.27 (d, 4H, *J* = 57.2 Hz, N–(CH_2_)_2_), 2.57 (d, *J* = 35.6 Hz, 4H, N–(CH_2_)_2_), 2.30 (s, 3H, N–CH_3_); ^13^C-NMR (100 MHz DMSO-*d*_6_) *δ* = 156.5, 151.6, 145.3, 128.9, 127.7, 127.6, 126.6, 126.3, 125.6, 124.0, 123.3, 122.9, 109.8, 54.4, 51.7, 45.6. MS: *m*/*z* = 296.1 (M^+^).

#### 2.2.5 4-(Piperidin-1-yl)-8-(trifluoromethyl)quinoline (4e)

Off white solid; yield: 60%; m.p.: 160–165 °C; ^1^H-NMR (400 MHz, CDCl_3_) *δ* = 8.86 (d, 1H, *J* = 5.2 Hz, TFMQ), 8.22 (d, 1H, *J* = 8.8 Hz, TFMQ), 8.01 (d, 1H, *J* = 7.2 Hz, TFMQ), 7.52 (t, 1H, TFMQ), 6.92 (d, 1H, *J* = 5.2 Hz, TFMQ), 3.20–3.17 (m, 4H, N–(CH_2_)_2_), 1.88–1.86 (m, 4H, (CH_2_)_2_), 1.73–1.70 (m, 2H, CH_2_); ^13^C-NMR (100 MHz DMSO-*d*_6_) *δ* = 157.4, 151.6, 145.4, 141.8, 129.4, 128.8, 127.6, 126.1, 123.5, 122.7, 109.8, 53.1, 25.5, 23.7. MS: *m*/*z* = 281.13 (M^+^).

#### 2.2.6 4-(4-Methylpiperidin-1-yl)-8-(trifluoromethyl)quinoline (4f)

Off-white solid; yield: 94%; m.p.: 168–173 °C; ^1^H-NMR (400 MHz, DMSO-*d*_6_) *δ* = 8.79 (d, 1H, *J* = 4.8 Hz, TFMQ), 8.25 (d, 1H, *J* = 8.4 Hz, TFMQ), 8.10 (d, 1H, *J* = 7.2 Hz, TFMQ), 7.65 (t, 1H, *J* = 8.0 Hz, TFMQ), 7.06 (d, 1H, *J* = 4.8 Hz, TFMQ), 3.47 (d, 2H, *J* = 12.0 Hz, (CH_2_)_2_), 2.80 (t, 2H, *J* = 32.0 and 11.6 Hz), 1.77 (d, 2H, *J* = 12.0 Hz), 1.58–1.45 (m, 3H), 1.00 (d, 3H, *J* = 6.4 Hz, CH_3_); ^13^C-NMR (100 MHz DMSO-*d*_6_) *δ* = 157.2, 151.5, 145.4, 128.9, 127.6, 126.6, 125.6, 123.5, 122.9, 109.8, 52.4, 33.7, 33.5, 30.1, 21.6. MS: *m*/*z* = 295.1 (M^+^).

#### 2.2.7 4-(2-Methylpiperidin-1-yl)-8-(trifluoromethyl)quinoline (4g)

Brown solid; yield: 59%; m.p.: 155–160 °C; ^1^H-NMR (400 MHz, CDCl_3_) *δ* = 8.81–8.76 (dd, 1H, *J* = 5.3 Hz, TFMQ), 8.13 (d, 1H, *J* = 10.0 Hz, TFMQ), 7.92 (d, 1H, *J* = 8.8 Hz, TFMQ), 7.43–7.39 (m, 1H, TFMQ), 6.83–6.71 (m, 1H, TFMQ), 3.42–3.35 (m, 2H, pip-H), 2.73–2.68 (m, 1H, pip-H), 2.43–2.38 (m, 1H, pip-H), 1.95–1.90 (m, 3H, pip-H), 1.89–1.79 (m, 1H, pip-H), 1.77–1.5 (m, 1H, *J* = 6.9 Hz, –CH_3_), 1.48–1.10 (m, 3H); ^13^C-NMR (100 MHz DMSO-*d*_6_) *δ* = 151.4, 144.6, 141.8, 129.4, 128.8, 127.1, 126.7, 126.4, 126.1, 125.2, 122.7, 122.4, 63.2, 52.0, 34.1, 26.1, 22.9, 17.7. MS: *m*/*z* = 295.3 (M^+^).

#### 2.2.8 4-(3,5-Dimethylpiperidin-1-yl)-8-(trifluoromethyl)quinoline (4h)

Brown solid; yield: 50%; m.p.: 192–197 °C; ^1^H-NMR (400 MHz, DMSO-*d*_6_) *δ* = 8.77 (d, 1H, *J* = 4.8 Hz, TFMQ), 8.25 (d, 1H, *J* = 8.4 Hz, TFMQ), 8.10 (d, 1H, *J* = 7.2 Hz, TFMQ), 7.66 (t, 1H, *J* = 8.0 and 7.6 Hz, TFMQ), 7.07 (d, 1H, *J* = 4.8 Hz, TFMQ), 3.47–3.38 (m, 2H, N–CH–H), 2.37 (t, 2H, *J* = 11.6 and 11.2 Hz, N–CH–H), 1.98–1.85 (m, 2H, CH–(CH_3_)_2_, 1.07 (d, 1H, *J* = 6.8 Hz, CH–H), 0.9 (d, 6H, *J* = 6.4 Hz, –(CH_3_)_2_), 0.80–0.71 (m, 1H, –CH–H); ^13^C-NMR (100 MHz DMSO-*d*_6_) *δ* = 156.8, 151.4, 145.3, 129.0, 127.7, 127.6, 125.6, 123.9, 123.4, 110.0, 109.8, 59.3, 58.9, 30.7, 26.9, 18.9, 18.5. MS: *m*/*z* = 309.60 (M^+^).

### 2.3. Single-crystal X-ray diffraction studies for compound (4a)

A Single-crystal of C_14_H_13_F_3_N_2_O with dimensions of 0.32 mm × 0.25 mm × 0.21 mm was chosen for X-ray diffraction analysis. X-ray intensity data for the crystallized compound were collected on a Bruker X8 APEX II diffractometer using Mo Kα radiation (*λ* = 0.71073 Å). The intensity data were collected at a temperature of 293 K. A total of 10 388 reflections were collected in the *θ* range 2.144° to 25.022°, of which 2259 reflections were found to be unique, and 1848 reflections had *I* > 2*σ*(*I*). A total of 182 parameters were refined against 2259 unique reflections.^48^ When the residual factor converged to *R* = 0.0607, the refinement was terminated; the weight factor *wR*_2_ was 0.1889, and the goodness of fit (*S*) =1.077.

### 2.4. Computational studies (Hirshfeld and DFT analysis)

The impact of intermolecular interaction on crystal packing in a molecular crystal is visualized and quantified by developing a Hirshfeld surface and its related 2D fingerprint plot using Crystal Explorer 17.5 software^49^ by providing the.cif as an input file obtained from the SCXRD analysis. To compute interaction energies within the molecule, the energy-density model of the B3LYP method with the 6-311++G(d,p) basis set is employed.^50^

One extremely effective method for determining the ground state characteristics of many-electron systems is density functional theory.^51^ The Gaussian 16 package was used in the current work to perform DFT calculations for the novel molecule, which were then displayed using Gaussview 6.0.^52^ The B3LYP functional and 6-311++G(d,p) basis set were used to successfully optimize the geometrical parameters in the gas phase without any symmetry constraints. The degree of agreement between the experimental and optimized crystal structures has been examined. Global chemical descriptors were computed and compared, and the HOMO–LUMO energies of the initial chemicals and the new cocrystal hydrate were obtained. Additionally, by creating the molecular electrostatic potential map, the electron-rich and electron-poor areas of the molecule were determined.

### 2.5. Larvicidal activity

**Fig. 2:**
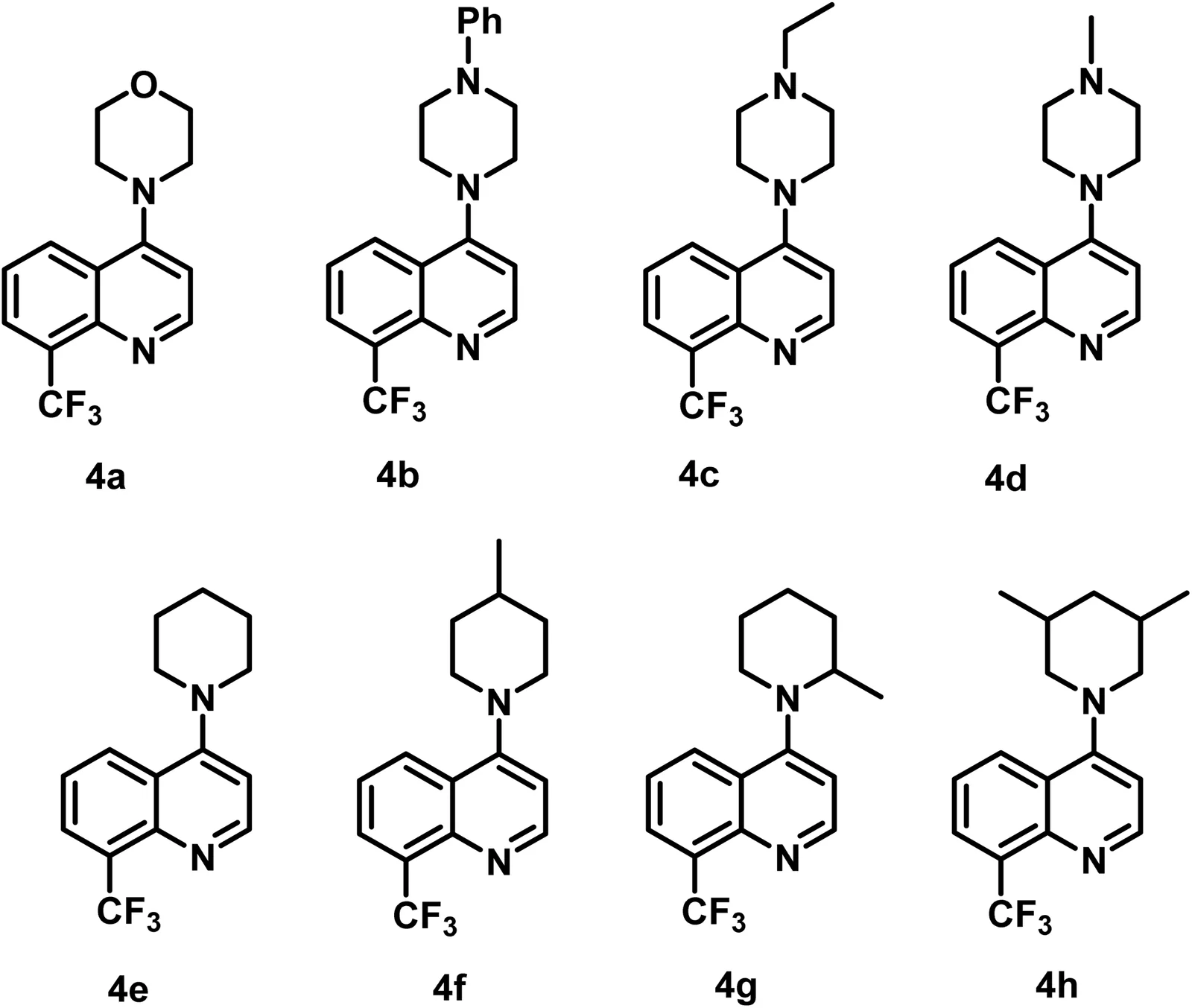
Chemical structures of compounds synthesized (4a–4h) and used for larvicidal activity against *Anopheles arabiensis*.

The larvicidal activity of selected synthesized compounds was assessed against *Anopheles arabiensis* following the World Health Organization (WHO, 1975) protocol under controlled insectary conditions (27.5 °C, 70% relative humidity, and lighting a 12 h light/12 h dark photoperiod).^53^ One milligram of test compound (Fig. 2) was added to 1 mL of acetone (1 mg mL^−1^), followed by 249 mL of distilled water to obtain a final concentration of 4 µg mL^−1^. Thirty individuals of 3rd instar larvae were introduced into each test container. A negative control was set up using the solvent (acetone) and distilled water, and a positive control included Temephos, an active emulsified organophosphate larvicidal used by malaria control programs. Larvae were fed specially made cat food that contained less oil/fat content. Larval mortality was recorded after 24 and 48 hours, and larvicidal activity was expressed as a percentage (or proportion) of mortality relative to the initial number of larvae initially exposed.

Additional dose–response bioassays were carried out for the most promising compounds (those resulting in >70% mortality) identified in the initial screening. Compounds 4b, 4c, 4d and Temephos as positive reference standard were further tested against *A. arabiensis* at 4.50, 4.00, 3.00, 2.00, 1.33, 0.89, 0.59 and 0.39 µg mL^−1^ final concentration; three replicates were performed per dilution series. An acetone solvent control (30 larvae × 24 replicates at 24 and 48 h) was run in parallel to monitor background mortality, which was low (3.3% after 48 h) and below the threshold typically warranting Abbott's correction.

#### 2.5.1 Data analysis

Larval mortality was analyzed using generalized linear models (GLM) with a quasibinomial error distribution to account for dispersion in R^54^ using RStudio.^55^ The response variable was the proportion of *Anopheles arabiensis* dead larvae, modeled as a function of treatment (compounds 4a to 4h, plus negative control acetone and positive control Temephos), and exposure time (24 hours and 48 hours). Estimated marginal means (EMMs) were obtained using the emmeans package in R.^56^ Tukey-adjusted pairwise comparisons were performed to identify significant differences among treatments, and results were summarized using compact letter displays, where treatments sharing the same letter are not significantly different (*p* < 0.05). Mortality means ± standard error (SE) are reported in Table 1.

**Table 1:** Larvicidal activity of the synthesized 4-substituted-8-(trifluoromethyl)quinolines (4a–4h) after 24 h and 48 h of exposure^a^

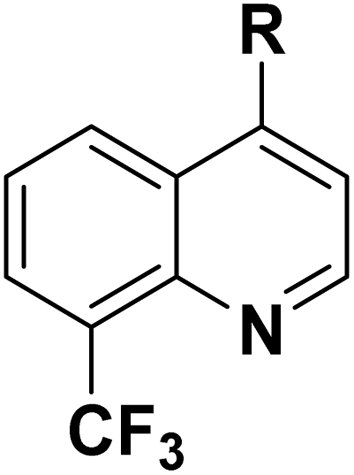			
Entry	R	Mortality	
		24 h	48 h
4a	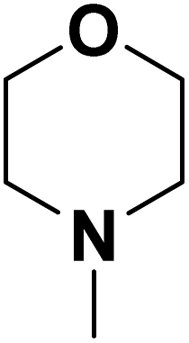	0.40 ± 0.03^bc^	0.44 ± 0.05^bc^
4b	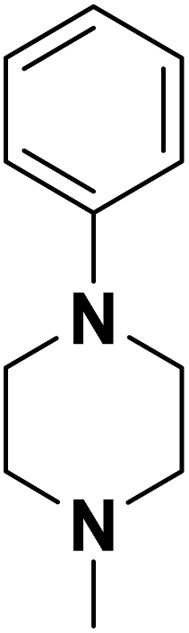	0.92 ± 0.04^d^	0.97 ± 0.03^dh^
4c	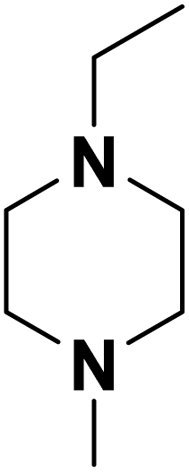	0.81 ± 0.02^e^	0.87 ± 0.03^e^
4d	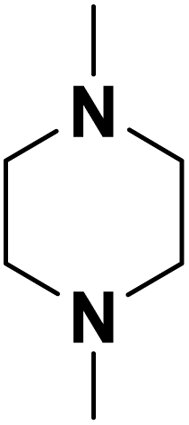	0.69 ± 0.02^f^	0.72 ± 0.02^f^
4e	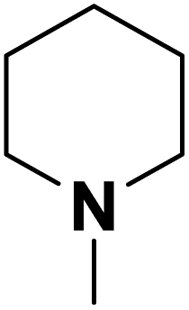	0.51 ± 0.04^cg^	0.56 ± 0.05 ^cg^
4f	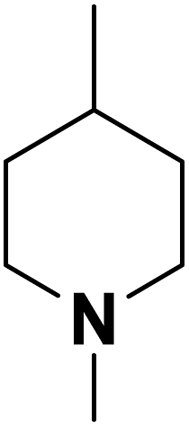	0.59 ± 0.02^fg^	0.62 ± 0.02 ^fg^
4g	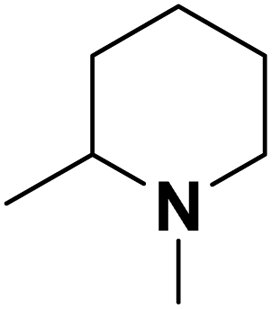	0.39 ± 0.04^bc^	0.43 ± 0.03^bc^
4h	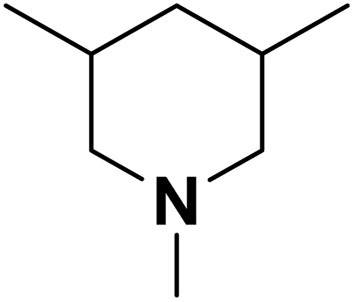	0.32 ± 0.02^b^	0.37 ± 0.03^b^
Acetone		0.01 ± 0.02^a^	0.03 ± 0.00^a^
Temephos		0.99 ± 0.02^h^	1.00 ± 0.00^h^

a a–h means with a common letter are not significantly different (*p* > 0.05).

Dose–response curves were fitted separately for each exposure time using two-parameter log-logistic models (equivalent to logistic regression on log-transformed concentration), implemented in R^54^ using RStudio,^55^ using the drc package^57^ and cross-validated with binomial generalized linear models (GLMs). Median (LC_50_) and LC_90_ lethal concentrations were estimated with 95% confidence intervals using Pearson chi-square test comparing observed and predicted mortality; because these indicated mild-to moderate extra-binomial variations in some groups (dispersion factors 0.33–1.98), standard errors and confidence intervals were recalculated using quasi-binomial GLMs, which adjust for overdispersion *via* a quasi-likelihood scale parameter without altering point estimates.^58^ Equality of slopes (parallelism) among the four dose–response curves was tested within each exposure time using a quasi-F test comparing nested quasi-binomial models with and without a compound × log(concentration) interaction term. Relative potency of each compound *versus* Temephos was expressed as the resistance ratio (RR = LC_50_ compound/LC_50_ Temephos), with 95% CI obtained *via* the delta method; RR was considered statistically significant when its CI excluded 1.

### 2.6. MTT cellular bioassay

The cytotoxic effects of the synthesized compounds (4a–4h) were evaluated against normal human dermal fibroblasts obtained from the American Type Culture Collection (ATCC), Manassas, VA, USA) using an MTT (3-[4,5-dimethylthiazol-2-yl]-diphenyltetrazolium bromide) colorimetric assay kit purchased from (Promega, USA). Normal fibroblast cells were cultured in Iscove's Modified Dulbecco's Medium (IMDM) (Euroclone S.p.A., Italy) supplemented with 10% fetal bovine serum (FBS), 1% (v/v) penicillin-streptomycin, and 1% (v/v) l-glutamine. All culture reagents were obtained from (Euroclone S.p.A., Italy), unless otherwise stated. Cells were maintained in an incubator at 37 °C under a humidified atmosphere containing 5% CO_2_ and 95% air in 25 cm^2^ or 75 cm^2^ cell culture flasks.

Fresh stock solutions of all compounds were prepared in culture-grade dimethyl sulphoxide (DMSO) (Santa Cruz Biotechnology, Dallas, TX, USA) and diluted in cell culture medium, ensuring that the final DMSO concentration did not exceed 0.1%. Cells were seeded into 96-well plates at a density of 5 × 10^3^ cells/well and allowed to attach overnight at 37 °C in a 5% CO_2_ atmosphere. Fibroblast cells were then treated with compounds 4a–4h at concentrations of 200, 100, 50, 10, 2, and 0.4 µM for 24 hours.

Following incubation, the culture medium was replaced with 100 µL of complete culture medium, and 10 µL of MTT dye solution was added to each well. The plates were incubated for an additional 4 h at 37 °C. Subsequently, 100 µL of the solubilization/stop solution was added, and the absorbance was measured at 570 nm using a Multiskan Go microplate reader (Thermo Fisher Scientific, Hampshire, UK).^59,60^ Cell Viability was calculated using the formula below:
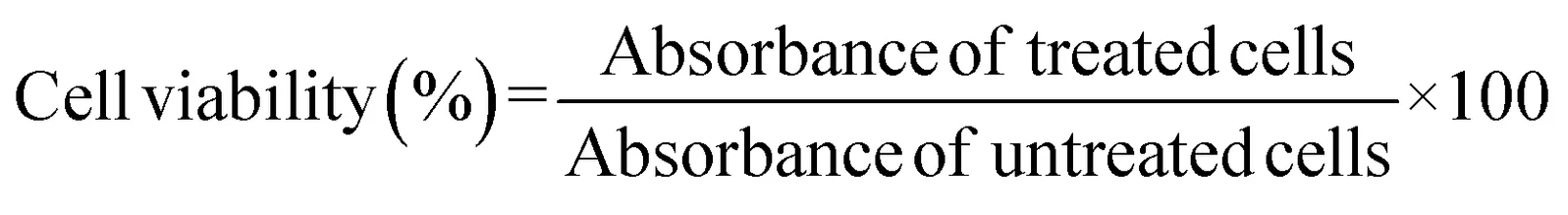


The IC_50_ values were determined using nonlinear regression analysis in GraphPad Prism version 9.3.1. All experiments were performed in triplicate across three independent experiments.

### 2.7. Molecular docking studies

In order to evaluate the larvicidal effects, all the designed title compounds 4a–4h are further, evaluated for molecular docking simulations to assess the potent mechanisms that were done against malarial vectors. The five proteins were selected (PDB ID: 1ZP4, 2CH2, 4JBV, 5V13, and 6ARY) retrieved from the PDB database (RCSB PDB).^61^ The protein and ligand preparations were done in AutoDock Vina tools.^62^ The co-crystal ligand and water molecules were removed. Polar hydrogens and Kollman charges were added to the proteins. All the ligands were prepared by adding Gasteiger charges and setting the torsional degrees of freedom. The docking grid was positioned to cover the active site of the protein (Table S5), with dimensions as reported in Tables S6–S10, for PDB ID: 1ZP4, 2CH2, 4JBV, 5V13, and 6ARY, respectively. Docking parameters were fine-tuned for optimal accuracy. The Lamarckian Genetic Algorithm (LGA) was used as the AutoDock suite.^63^ The output file was generated, and binding scores were analyzed. Interaction analysis and pose visualization were conducted using Discovery Studio.^64^

### 2.8. *In silico* ADMET studies

ADMET studies encompass absorption, distribution, metabolism, excretion, and toxicity of drugs, providing an essential assessment of their *in vivo* behavior in the human body and predicting their clinical efficacy by evaluating their pharmacokinetic effects.

It has been found that in recent years, various computational tools have been developed to predict ADMET properties. In *silico* methods use tools, such as machine learning (ML), artificial intelligence (AI), like pkCSM,^65^ ADMETlab 3.0,^66^ SWISS ADME tool,^67–69^ vNN-ADMET,^70^ ADMETboost,^71^ and ADMET-AI.^72^ ADMET profiles used to study properties such as solubility, bioavailability, intestinal absorption, plasma protein binding, liver enzymes, biliary excretion, and hERG inhibition, *etc.*, are generated using the SWISS ADME and pkCSM web servers (Table S4).

### 2.9. Toxicity studies

*In silico* toxicity screening plays a very important role in assessing the safety and toxicity of chemicals and drugs. Using the PKCSM web server,^65^*in silico* toxicity profiles leading to various adverse effects can be predicted, such as *in vitro* hERG channel blockages, hepatotoxicity (DILI), nephrotoxicity, neurotoxicity, and skin sensitization.

## 3. Results and discussion

### 3.1. Chemistry

**Scheme 1:**
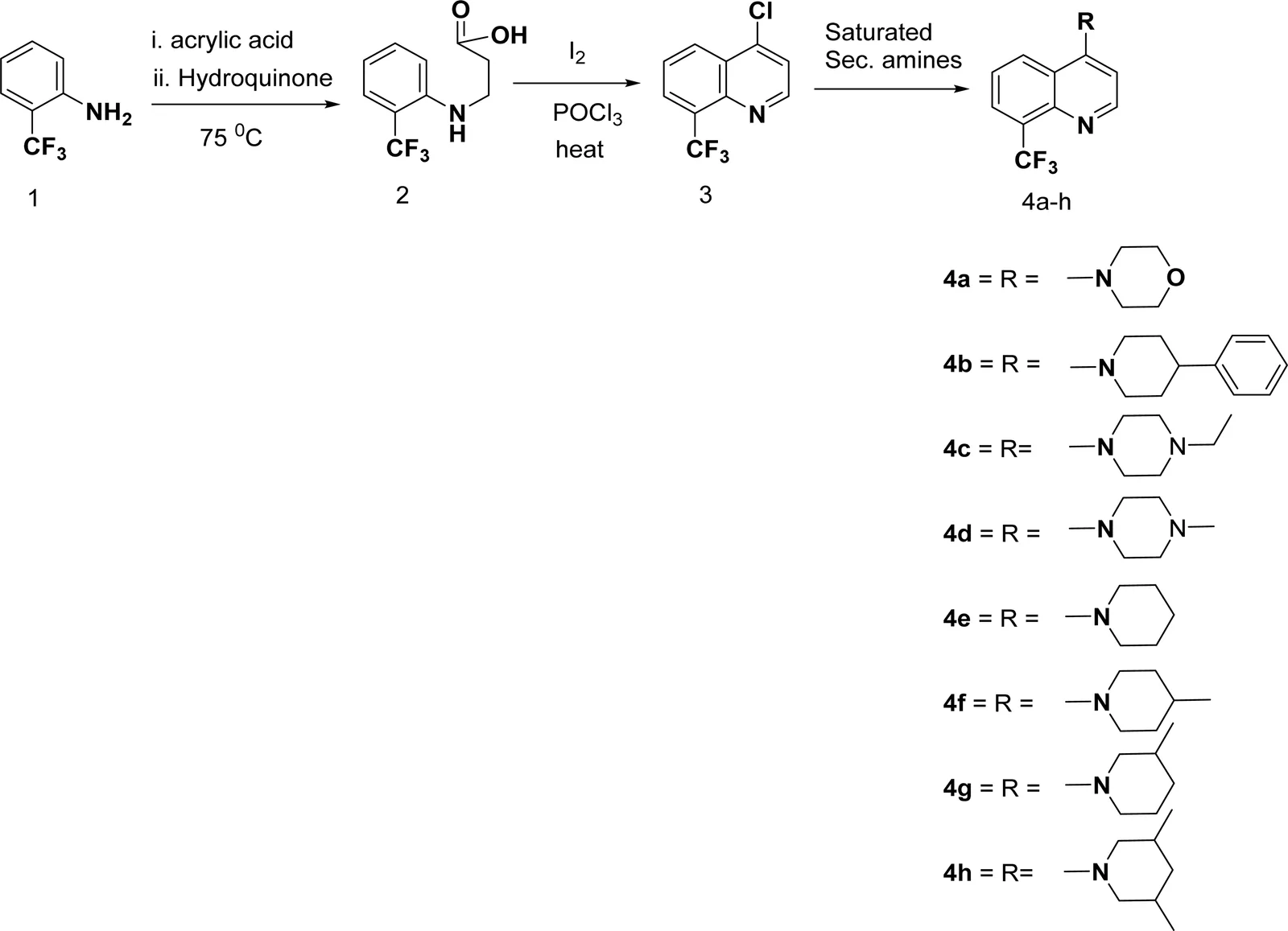
Synthetic route for the preparation of 8-trifluoromethyl quinoline derivatives bearing substituted saturated amines (4a–4h).

8-Trifluoromethyl quinoline derivatives bearing substituted saturated amines (4a–4h) were synthesized by the reaction of 4-chloro-8-trifluoromethyl quinoline 4 with various substituted saturated amines in ethanol at reflux temperature in the presence of base triethylamine (Scheme 1). The synthesized compounds were characterized using ^1^H-NMR, ^13^C-NMR, and mass spectral studies. The ^1^H-NMR spectrum of compound 4a displayed both the N–CH_2_ and O–CH_2_ protons as multiplet in the region *δ* 4.03–4.01 and 3.26–3.23. Four distinct doublets at *δ* 8.92 (with a coupling constant *J* = 4.8 Hz), 8.27 (with a coupling constant *J* = 8.0 Hz), 8.05 (with a coupling constant *J* = 7.2 Hz), and 6.98 (with a coupling constant *J* = 4.8 Hz) ppm were assigned to the four aromatic protons of trifluoromethyl quinoline, and one proton appears as a multiplet at *δ* 7.57 ppm and thus confirming the formation of the desired product. The ^13^C-NMR spectrum of compound 4a showed a peak at *δ* 124.0 ppm, which corresponds to the trifluoromethyl carbon. The carbon peaks at *δ* 66.8 and 52.7 ppm correspond to the four carbon atoms of the morpholine ring. The rest of the aromatic carbon peaks appeared in the range of *δ* 156.9–109.5 ppm. The molecular ion peak at *m*/*z* 282.9 (M^+^) for the targeted compound 4a was confirmed and consistent with the molecular formula C_14_H_13_N_2_F_3_O.

### 3.2. Single-crystal X-ray studies for 4-(8-(trifluoromethyl)quinolin-4-yl)morpholine (4a)

**Fig. 3:**
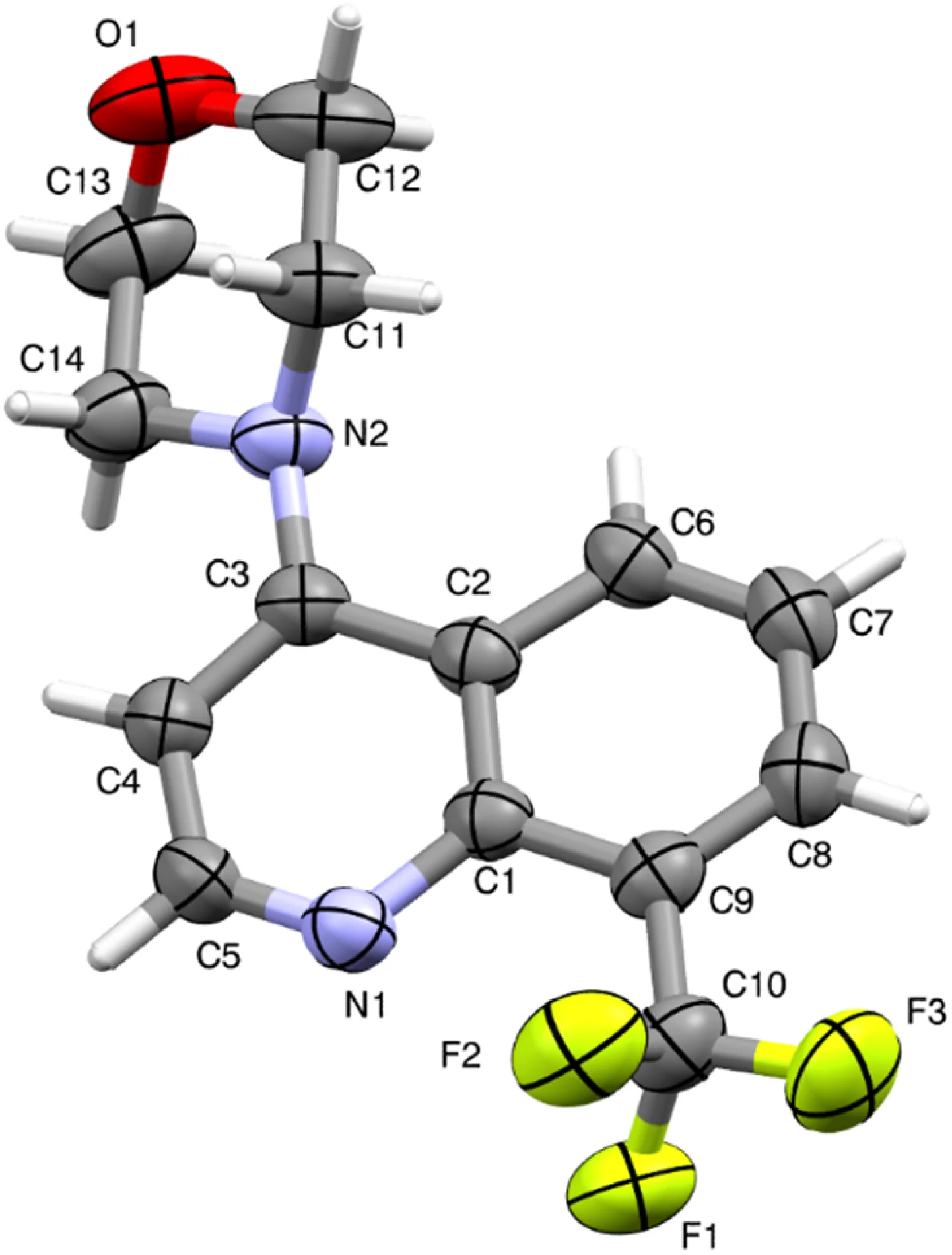
An atom-labeled ORTEP diagram drawn at 50% probability of the title compound 4a.

The title molecule 4a comprises of trifluoromethyl quinoline (N1/C1–C9) ring and morpholine (Cg1:N2/C11/C12/O1/C13/C14) moiety. The dihedral angle between the mean plane of quinoline and morpholine ring is found to be 42.57(9)°, which confirms the non-planarity as well as bisectional orientation of the molecule. In a molecule, the morpholine ring exhibits the puckering characteristics and adopts the chair conformation with a maximum puckering amplitude *Q* = 0.577(2) Å and related phase angle *φ* = 37(13)°.^73^ An Oak Ridge Thermal Ellipsoid Plot (ORTEP) of the molecule is shown in Fig. 3. The torsion angles between the atoms N1–C1–C6–C10 and C10–C6–C7–C8 are −3.02(2) and 177.73(18), respectively, indicating that the trifluoromethyl group lies within the mean plane of the quinoline ring. All the atoms in the quinoline ring are sp^2^ hybridized, whereas the atoms in the morpholine rings are sp^3^ hybridized. Furthermore, the molecule exhibits C8–H8⋯O1 intermolecular interactions and weak intramolecular C7–H7⋯F1 and C9–H9⋯N2 interactions. Additionally, the crystal structure is stabilized by π⋯π interactions of the type Cg2⋯Cg3 exists with a distance of 3.7588(10) Å.

### 3.3. Structural analysis

The crystallographic data obtained from XRD analysis, along with details of the structure refinement^74^ of compound 4a, are presented in Table 2. From XRD analysis, it was confirmed that the compound crystallizes in the monoclinic lattice with space group *P*2_1_/*n*. The atom labeled ORTEP^75^ of the molecule drawn at 50% probability is shown in Fig. 3. The experimentally obtained geometrical parameters, such as bond lengths, bond angles, and torsion angles, are listed in Tables S1–S3, respectively. The values are in good agreement with the reported values.^73,76^

**Table 2:** Crystal data and details of structure refinement of compound 4a

CCDC	2202316
Chemical formula	C_14_H_13_F_3_N_2_O
Molecular weight	283.27
Crystal system	Monoclinic
Space group	*P*2_1_/*n*
Unit cell dimensions (Å)	*a* = 11.5185(10), *b* = 10.2985(6), *c* = 11.5827(10)*β* = 110.652(10)°
Volume (Å^3^)	1285.69(19)
*Z*	4
Density (Mgm^−3^)	1.458
Absorption coefficient *µ* (mm^−1^)	0.123
*F*_000_	584
Crystal size (mm)	0.21 × 0.25 × 0.32
Diffractometer	Bruker X8 APEX II diffractometer
Wavelength (*λ*) Å and radiation	0.71073 and MoKα
Temperature (K)	293
*θ* range for data collection	2.144 to 25.022
Index ranges	*h* = −15 → 15; *k* = −14 → 14; *l* = −14 → 15
Refinement method	Full matrix least-squares on *F*^2^
Reflections collected	10 388
Independent reflections	2259
Observed reflections [*I* > 2*σ*(*I*)]	1848
*R*_int_	0.042
*N*_ref_, *N*_par_	2 274 182
*R* Indices	*R*_1_ = 0.0607, w*R*_2_ = 0.1889
Goodness-of-fit	1.077
Δ*ρ*_max_, Δ*ρ*_min_ eÅ^−3^	−0.50 and 0.35

The title compound 4a is constituted by two rings: trifluoromethyl quinoline and the morpholine rings. The dihedral angle between the mean planes of quinoline (N1–C1–C2–C3–C4–C5–C6–C7–C8–C9) and morpholine (C12–C13–C14–C15–C16–C17) rings is in nearly bisectional orientation as indicated by the value of 42.57(9)°. The morpholine ring is puckered and adopts the chair conformation, with a maximum puckering amplitude *Q* = 0.577 (2) Å, *θ* = 0.0 (2)°, and a related phase angle *φ* = 37(13)°. The torsion angle is found to have 179.23(16)°, and 177.73(18)° between the atoms C2–C1–C9–C10, and C7–C8–C9–C10, respectively, indicating that the trifluoromethyl group lies within the mean plane of the quinoline ring and also exhibits the −syn-periplanar and +anti-periplanar orientation. The fused quinoline ring exhibits predominantly sp^2^ hybridization, associated with aromatic π-electron delocalization and planarity, whereas the morpholine moiety displays sp^3^ hybridization, consistent with its puckered chair conformation. The bond length of O1–C12 = 1.418(3) Å and O1–C13 = 1.418(3) Å depicts the single bond character.

**Fig. 4:**
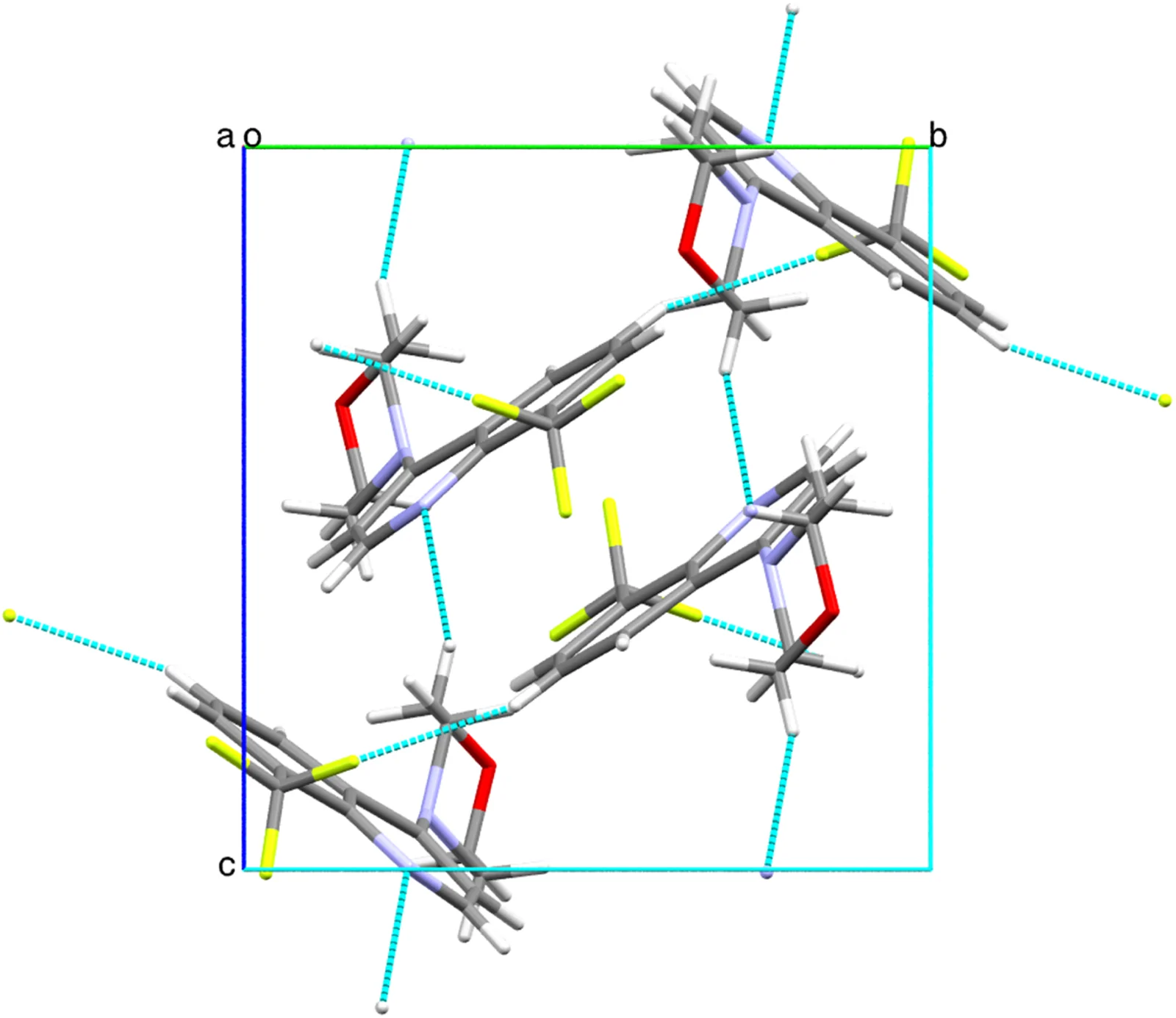
Packing view of compound 4a along the *a* axis.

The packing of compound 4a is stabilized by an intermolecular interaction of the type C7–H7⋯O1 with symmetry 1/2 − *x*, 1/2 + *y*, 1/2 − *z*, and a weak intramolecular interaction of the type C6–H6⋯N2 and C8–H8⋯F3 are mentioned in Table 3*.* The structure of compound 4a shows C10–F3⋯Cg2 interaction observed between C10–F3 atoms and the centroid of the ring (N1–C1–C2–C3–C5–C5) with H–cg distance of 3.9412(17) Å, *γ* = 19.59°, C10–cg2 = 5.032 Å and the angle of C10–F3 with π plane is 67.72° respectively. The molecular packing diagrams along the crystallographic *a-axis* are shown in Fig. 4.

**Table 3:** Hydrogen bond geometry of compound 4a

Atoms	D–H (Å)	H⋯A (Å)	D–A (Å)	D–H⋯A (◦)
C7–H7⋯O1^a^	0.93	2.55	3.259(3)	100
C6–H6⋯N2^bb^	0.93	2.61	2.913(2)	133
C8–H8⋯F3^b^	0.93	2.32	2.670(3)	102

a Symmetry code: 1/2 − *x*, 1/2 + *y*, 1/2 − *z*.

b Intra molecular interaction.

### 3.4. Hirshfeld surfaces (HS), 2-D fingerprint plots, and DFT calculations of compound 4a

**Fig. 5:**
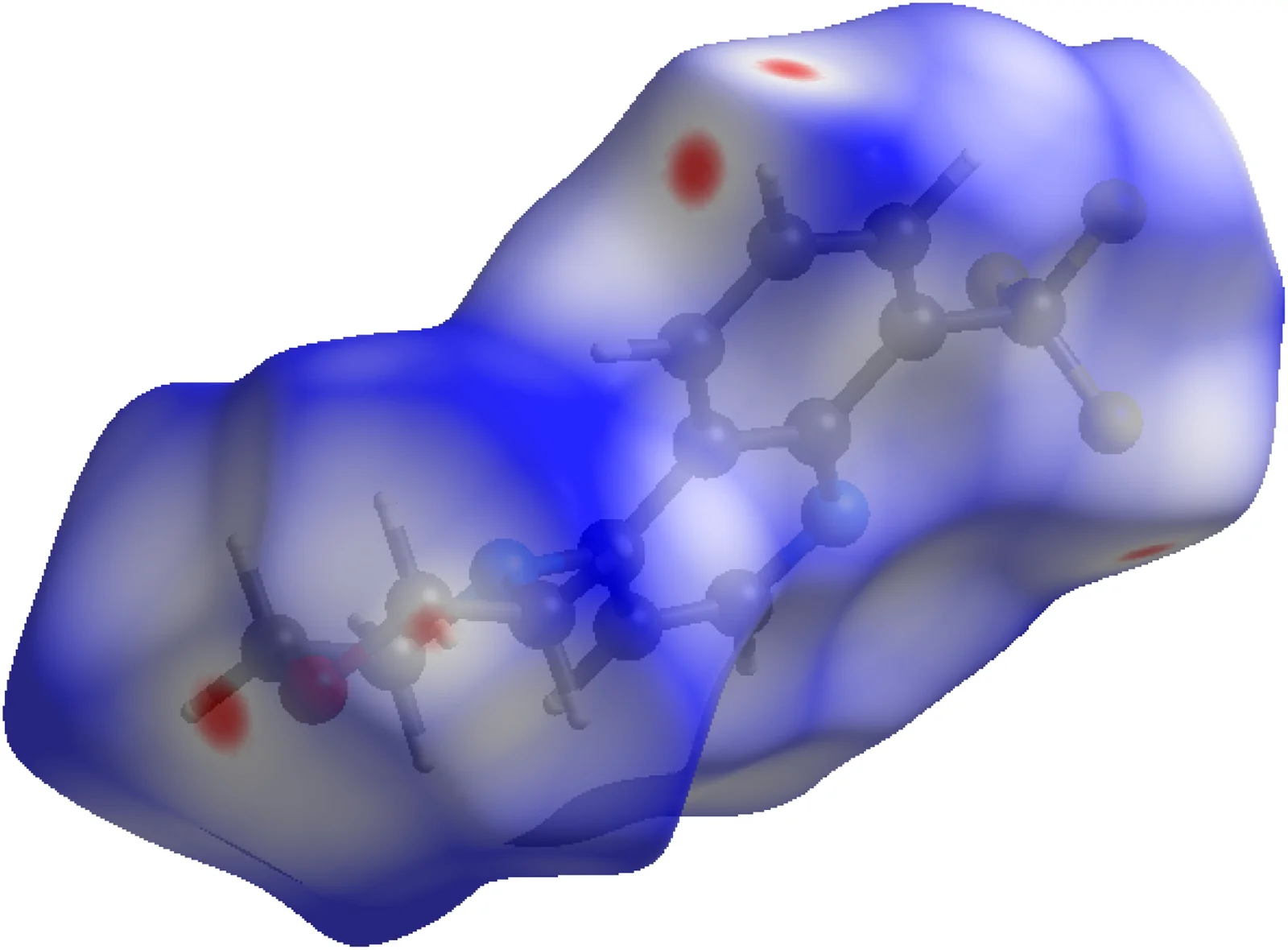
Hirshfeld surface mapped on *d*_norm_ of compound 4a.

The chemical behavior of the molecule is directly related to the intra and intermolecular interactions that take place in a molecule; hence, the understanding and visualization of these interactions, Hirshfeld surfaces, and a 2D fingerprint plot were generated by using Crystal Explorer 17.5. The Hirshfeld surface mapped over *d*_norm_ of the title molecule was generated with a color scale ranging from −0.0833(red) to −1.388(blue). Electrostatic potential mapped to the Hirshfeld surface is shown in Fig. 5. On this map, blue and red regions correspond to positive and negative potentials on the HS mapped over the electrostatic potential. In Fig. 5, a dark red circular spot on the surface predicts the C8–H8⋯O1 intermolecular hydrogen interaction. The HS analysis validates the intermolecular interactions observed in the single-crystal XRD structure analysis.

The Hirshfeld surface mapped on *d*_norm_ is shown in Fig. 5. The intensely red-colored circular spots appear on the *d*_norm_ highlights that the donor and acceptor atoms participate in an intermolecular C7–H7⋯O1 interaction.

The two-dimensional fingerprint plot displays the breakdown of the Hirshfeld surface into the relative contribution of different intermolecular interactions existing in the crystal structures shown in Fig. S30. The H⋯H interaction contributed the most (33.2%), appearing as two symmetrical broad peaks in the middle region of the fingerprint plot 1.2 Å (*d*_e_ + *d*_i_) < 1.19 Å. A pair of C⋯H/H⋯C interatomic contacts gives the other significant contribution of 12.4%, which appears in the form of blue colored symmetrical wings around the region of 1.18 Å (*d*_e_ + *d*_i_) 1.78 Å. The H⋯N/N⋯H pair of inter-atomic contacts made 5.8% of the contribution reflected as concave spikes in the region of 1.10 Å (*d*_e_ + *d*_i_) 1.48 Å. The O⋯H/H⋯O pair of contacts made 6.1% of significant contribution appears in the region of 1.10 Å. The least contribution from C⋯F/F⋯C, N⋯C/C⋯N, and O⋯C/C⋯O, also contributes to the total area of the Hirshfeld surface.

### 3.5. Three-dimensional energy frameworks

The interaction energies for the selected molecule (4a) were calculated by constructing a cluster of various color-coded molecules within a radius of 3.8 Å. The red-colored solid cylinders connecting the molecules represent the coulombic interactions; green-colored solid cylinders highlight the dispersion interaction energy; and blue-colored solid cylinders represent the total interaction energy, as depicted in Fig. S31. The total energy components are shown in Table S11. Accordingly, the highest total interaction energy (*E*_tot_ = −57.9 kJ mol^−1^) in the cluster was noticed for an ink blue colored molecule interacting at the least molecular centroid distance of *R* = 4.20 Å associated with the rotational symmetry code of −*x*, −*y*, −*z*. A pair of red colored molecules interacting at the centroid distance of *R* = 11.52 Å showed the least interaction energy of (*E*_tot_ = −7.0 kJ mol^−1^) associated with the symmetry code of *x*, *y*, *z*. The molecule's total energies were calculated by adding the electrostatic (*E*_ele_), dispersion (*E*_ele_), polarization (*E*_pol_), and repulsion (*E*_rep_) energies. Computed total interaction energies of compound 4a are electrostatic (−48.83 kJ mol^−1^), dispersion (−184.64 kJ mol^−1^), polarization (−10.65 kcal mol^−1^), and repulsion (57.53 kJ mol^−1^). The combination of these energies provides the total energy (−301.65 kJ mol^−1^). In the crystal environment, dispersion energy dominates over electrostatic and polarization energies.

### 3.6. Density functional theory (DFT) calculations

**Fig. 6:**
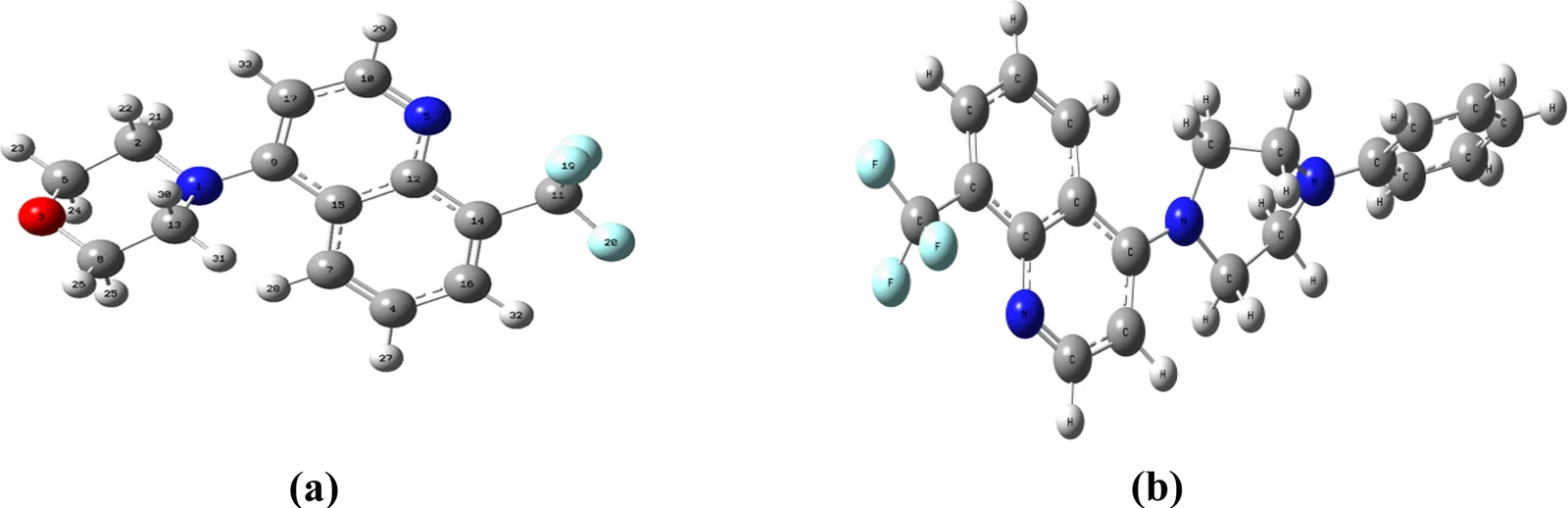
Optimized structure of the title molecule (a) 4a (b) 4b.

It is preferable to determine the optimized structure for the title compounds 4a and 4b (Fig. 6). The optimized structural parameters are listed in Table 4 in accordance with the atom labeling in Fig. 6 and were calculated using the basis set B3LYP/6-311 ++ G (d,p).

**Table 4:** Comparison of the experimental and optimized geometric parameters of the title compound 4a

Atom	Bond length (Å)		Atom	Bond angle (°)		Atom	Torsion angle (°)	
	XRD	DFT		XRD	DFT		XRD	DFT
F1–C10	1.336(3)	1.3363	C12–O1–C13	109.74(2)	109.8415	N1–C1–C2–C3	2.7(2)	2.6091
F2–C10	1.333(2)	1.3335	C1–N1–C5	116.05(2)	116.1103	N1–C1–C9–C8	176.28(17)	176.31
F3–C10	1.341(3)	1.3417	C3–N2–C11	116.33(1)	116.5258	C9–C1–C2–C3	−179.69(15)	−179.613
O1–C12	1.418(3)	1.416	C3–N2–C14	116.41(2)	116.4614	N1–C1–C2–C6	−173.65(16)	−173.9705
O1–C13	1.418(3)	1.4182	C11–N2–C14	109.46(2)	109.3223	C2–C1–C9–C10	179.23(16)	179.2338
N1–C1	1.366(2)	1.366	N1–C1–C2	123.37(1)	123.3589	C2–C1–C9–C8	−1.5(3)	−1.5863
N1–C5	1.315(2)	1.3147	N1–C1–C9	117.98(2)	118.0885	N1–C1–C9–C10	−3.02(2)	−2.8699
N2–C3	1.406(2)	1.4058	C2–C1–C9	118.62(2)	118.5168	C6–C2–C3–N2	−7.2(3)	7.239
N2–C11	1.462(3)	1.4623	C1–C2–C3	117.63(1)	117.5769	C3–C2–C6–C7	−179.70(17)	−179.7366
N2–C14	1.464(3)	1.4655	C1–C2–C6	118.87(2)	118.8708	C1–C2–C6–C7	−3.6(3)	−3.689
C1–C2	1.418(3)	1.4179	C3–C2–C6	123.40(2)	123.4399	C6–C2–C3–C4	170.02(17)	169.8733

### 3.7. HOMO–LUMO energy calculation for compound 4a and 4b

**Fig. 7:**
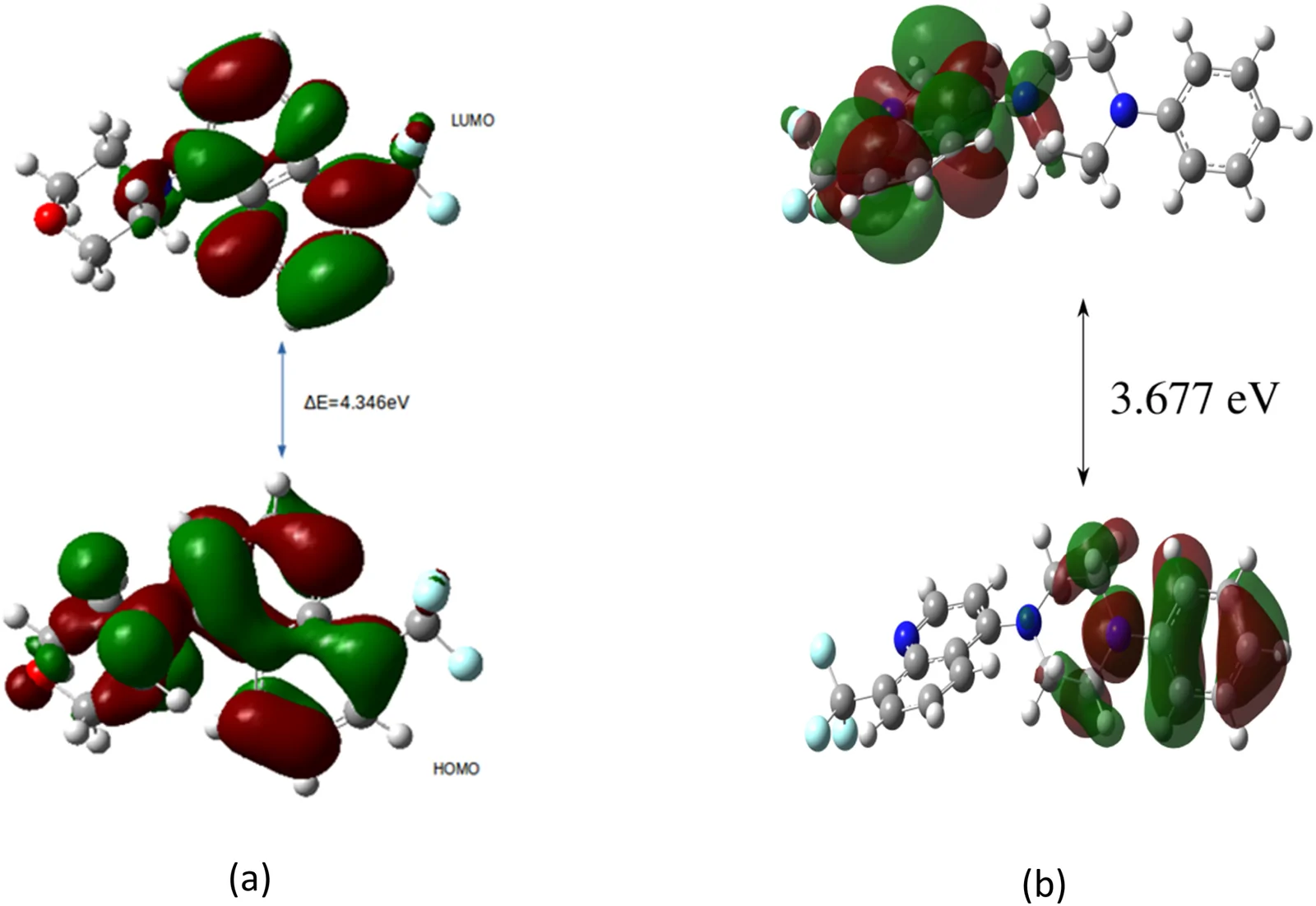
HOMO–LUMO of compound (a) 4a and (b) 4b.

The HOMO and LUMO molecular orbitals are delineated in Fig. 7. The calculated E_HOMO_ and E_LUMO_ are −6.50 eV and −2.154 eV, respectively. The gap between the HOMO and LUMO is known as the energy gap, and it is found to be 4.35 eV for compound 4a and 3.677 eV for compound 4b respectively. The energy difference between the HOMO and LUMO for compound 4a is high compared to 4b, hence, the molecule 4b is kinetically more stable and fast reactive. The calculated global descriptive parameters for the compound 4a and 4b is mentioned in Table 5.

**Table 5:** Global descriptive parameters of compound 4a and 4b

Chemical parameters	Values	
	4a	4b
*E*_HOMO_	−6.50 eV	−5.6866
*E*_LUMO_	−2.154 eV	−2.0096
*E*_gap_	4.35 eV	3.6771
Ionization potential (*I*)	6.50 eV	5.6866
Electron affinity (*A*)	2.1547 eV	2.0096
Electronegativity (*χ*)	4.327 eV	3.8481
Chemical hardness (*η*)	2.173 eV	1.8385
Global softness (*σ*)	0.460 eV^−1^	0.5439
Electrophilicity (*ω*)	4.231 eV	4.0271
Chemical potential (*µ*)	−4.327 eV	−3.8481

### 3.8. Molecular electrostatic potential map of compound 4a and 4b

**Fig. 8:**
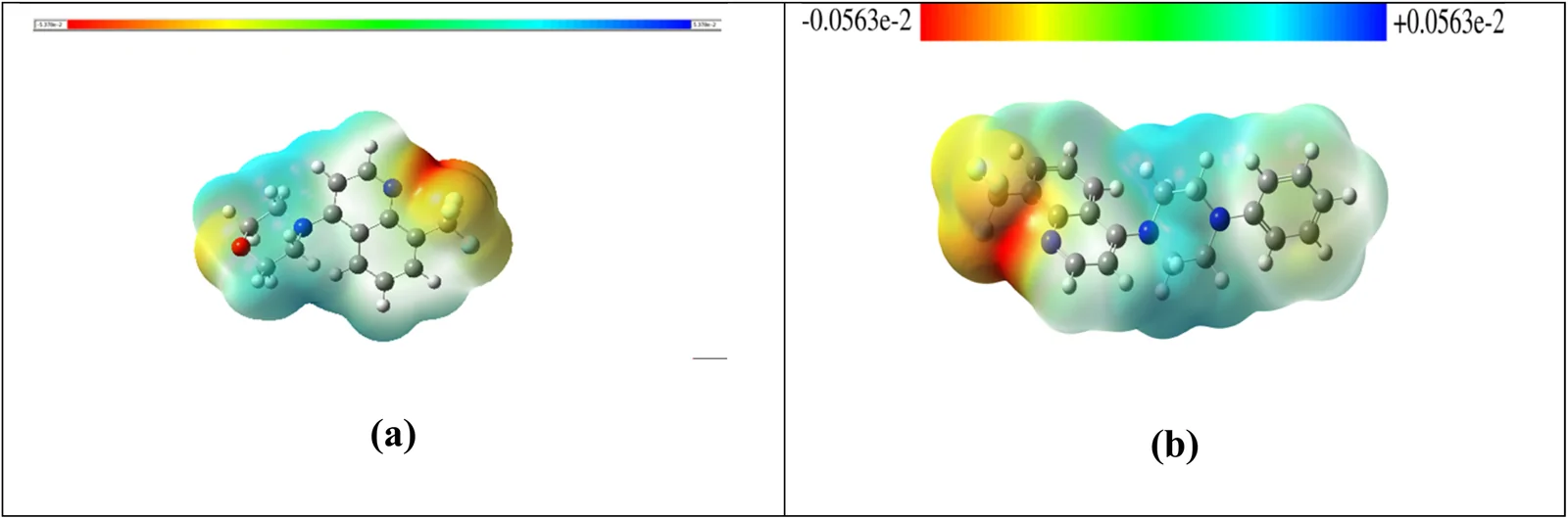
MEP (molecular electrostatic potential) of compound (a) 4a and (b) 4b.

The molecular electrostatic potential map of the title compounds 4a and 4b is shown in Fig. 8. The color code for these maps ranged from −6.330 × 10^−2^ a.u. to +6.330 × 10^−2^ a.u for compound 4a and from −0.0563 × 10^−2^ to +0.0563 × 10^−2^ for compound 4b. The red color on the surface indicates the electron-rich region can be observed near the fluorine atom for both compounds 4a and 4b, but the electron-rich region is more active for compound 4b, which can be seen at the fluorine atoms. whereas the blue color indicates the electron-deficient region. Hence, the oxygen atom (O1) acts as an acceptor, indicated in blue color.

### 3.9. Larvicidal activity

**Fig. 9:**
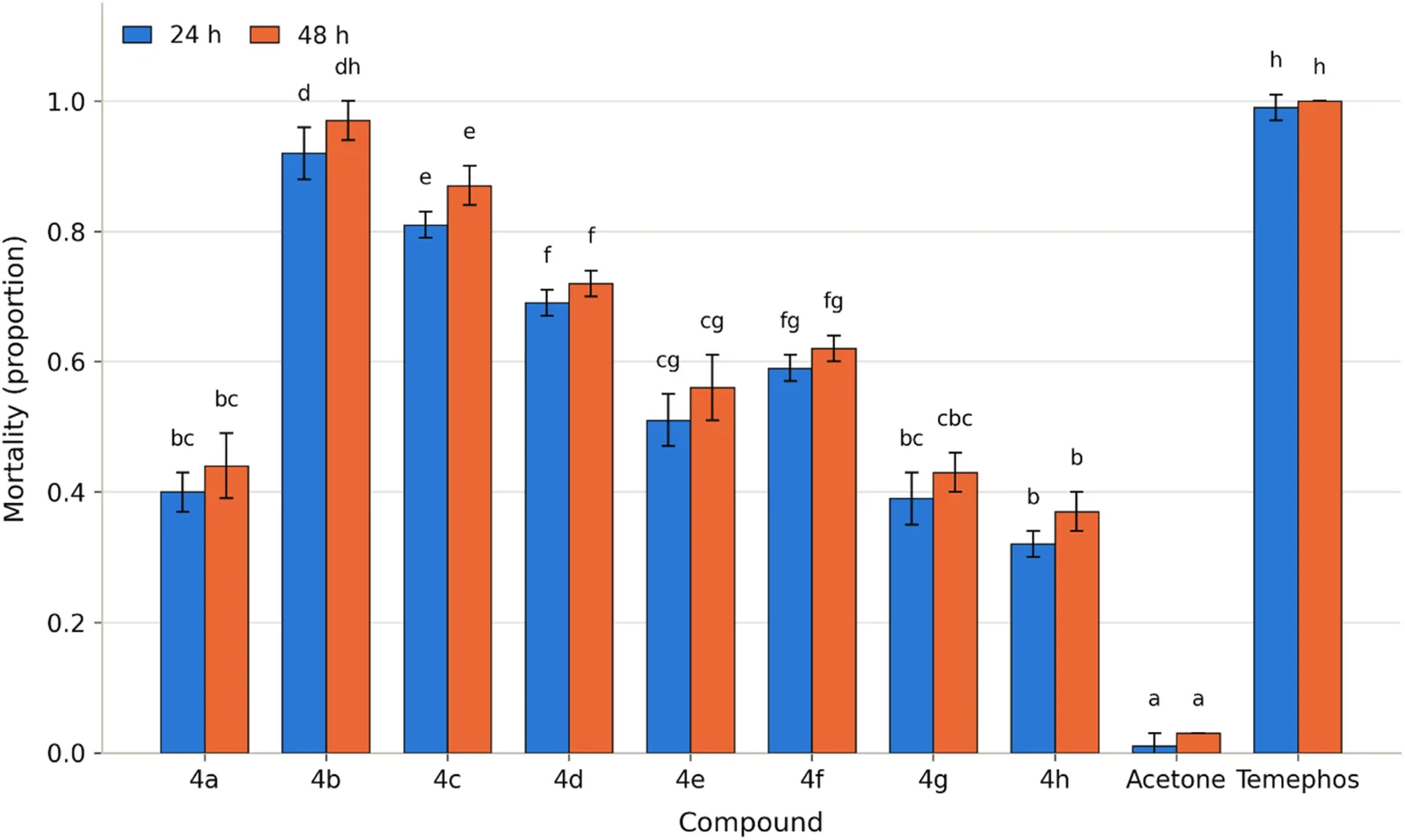
Larval mortality of *Anopheles arabiensis* from 24 h and 48 h of exposure to the synthesized 4-substituted-8-(trifluoromethyl)quinolines (4a–4h).

All synthesized compounds showed significantly higher larvicidal activity than the acetone control (*p* < 0.05) (Table 1 and Fig. 9). No significant effect of exposure duration (24 h *vs.* 48 h) was detected, nor were there significant treatment x time interactions. Pairwise comparisons using Tukey-adjusted post hoc indicated that compound 4b demonstrated the highest larvicidal activity, producing mortality rates of 92% and 97% after 24 h and 48 h, respectively, which were not significantly different from Temephos. Compound 4c also exhibited strong activity, with mortality rates reaching 81% and 87% after 24 h and 48 h, respectively. In contrast, compounds 4d and 4f showed moderate larvicidal effects, whereas compounds 4a, 4e, 4g, and 4h displayed comparatively lower activity. The rapid onset of mortality observed for compounds 4b and 4c suggests that their larvicidal action occurs within the first 24 h of exposure, with only marginal increases observed at 48 h.

**Fig. 10:**
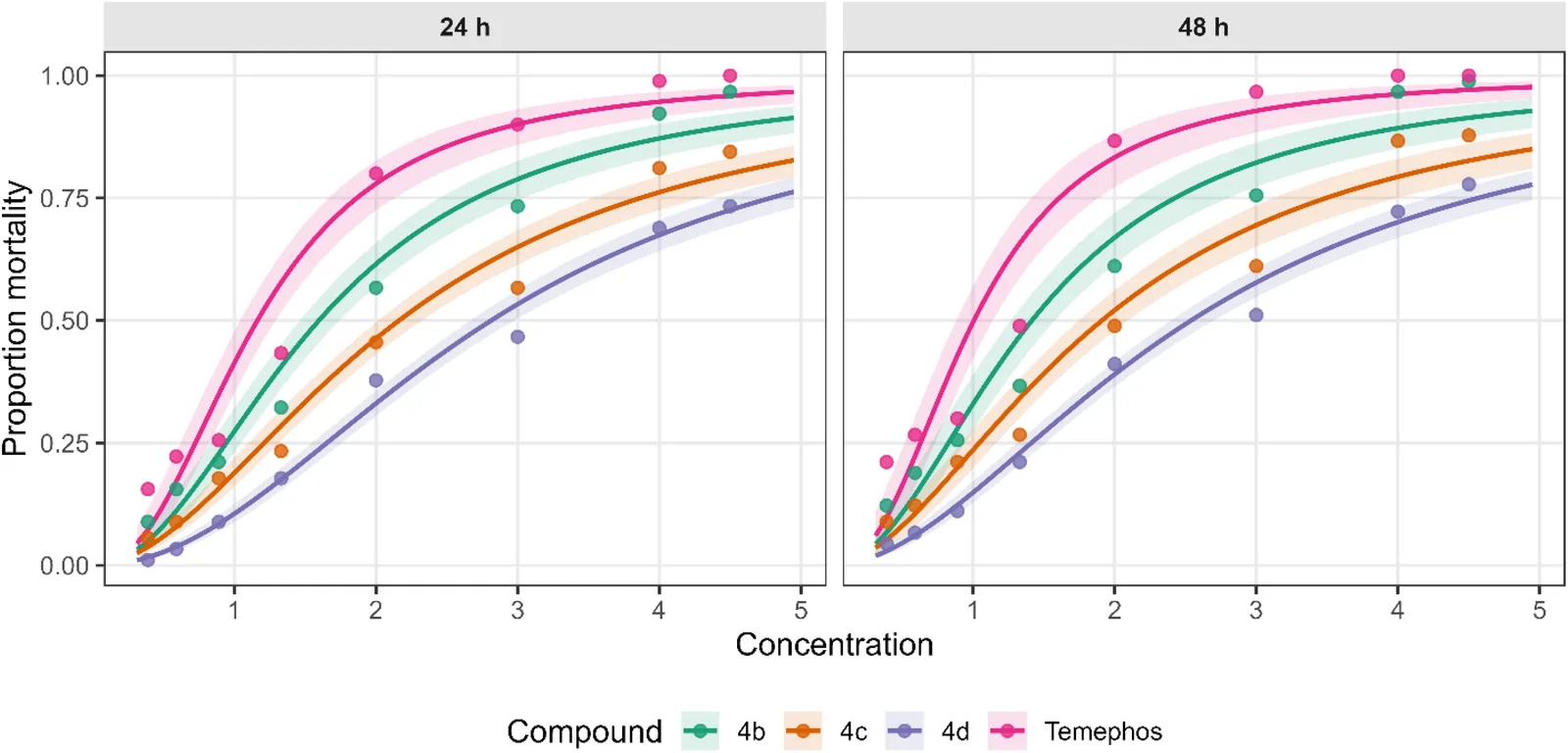
Dose–response curves for larval mortality of compounds 4b, 4c, 4d and Temephos at 24 and 48 h.

Dose–response curves for the most promising compounds 4b, 4c, 4d and the positive control Temephos showed a typical sigmoidal relationship between concentration and larval mortality at both 24 and 48 h (Table 6 and Fig. 10). Slopes did not differ significantly among compounds at either exposure time (quasi-F test, 24 h, *F*_3,88_ = 1.77, *p* = 0.158; 48h, *F*_3,88_ = 2.17, *p* = 0.097), indicating that the dose–response curves were statistically parallel and that comparisons based on LC_50_ are valid.

**Table 6:** Larval mortality dose–response parameters. Concentration expressed in µg mL^−1^. Compounds 4b, 4c, and 4d were compared against Temephos (positive control). *N* = 24 larvae per compound per exposure time (8 concentrations × 3 replicates; 30 larvae per replicate). Estimates and 95% confidence intervals (CI) were corrected for overdispersion using quasibinomial generalized linear models. Slopes of the four dose–response curves did not differ significantly within either exposure time (quasi-F test, 24 h, *p* = 0.158; 48 h *p* = 0.097), indicating parallel curves and validating direct LC_50_ comparisons

Compound	Time (h)	Slope ± SE	LC_50_ (95% CI)	LC90 (95% CI)	RR50 *vs.* Temephos (95% CI)
4b	24	2.09 ± 0.14	1.60 (1.46–1.74)	4.58 (3.91–5.36)	1.37 (1.20–1.57)^a^
4c	24	1.89 ± 0.14	2.16 (2.02–2.32)	6.92 (5.96–8.02)	1.86 (1.64–2.11)^a^
4d	24	2.07 ± 0.16	2.81 (2.66–2.97)	8.14 (7.21–9.20)	2.42 (2.15–2.73)^a^
Temephos	24	2.33 ± 0.16	1.16 (1.05–1.29)	2.99 (2.52–3.56)	Ref.
4b	48	2.04 ± 0.14	1.42 (1.27–1.58)	4.16 (3.43–5.03)	1.41 (1.19–1.66)^a^
4c	48	1.82 ± 0.13	1.91 (1.75–2.09)	6.39 (5.32–7.68)	1.90 (1.63–2.21)^a^
4d	48	1.87 ± 0.15	2.54 (2.39–2.69)	8.21 (7.20–9.37)	2.52 (2.19–2.90)^a^
Temephos	48	2.34 ± 0.16	1.01 (0.89–1.14)	2.57 (2.11–3.15)	Ref.

a RR significant (95% CI excludes 1).

All three synthesized compounds were statistically less toxic to larvae than the positive control Temephos at both 24 and 48 h, as reflected by resistance ratios greater than 1 with 95% CIs excluding unity (Table 6). The closest potency to Temephos was showed by compound 4b (RR_50_ = 1.37 at 24 h and 1.41 at 48 h), followed by 4c (RR_50_ = 1.86 and 1.90, respectively) and 4d (RR_50_ = 2.42 and 2.52 at 24 and 48 h, respectively). None of the tested compounds matched or exceeded the larvicidal potency of Temephos at either exposure time.

### 3.10. MTT cellular bioassay

The cytotoxic effects of the synthesized compounds were evaluated using the MTT bioassay on normal fibroblast cells. Most compounds demonstrated low cytotoxicity, with IC_50_ values exceeding 100 µM. Among the tested compounds, compounds 4a, 4b, 4c, and 4d demonstrated the most favorable safety profiles, with IC_50_ values greater than 200 µM (Table 7). However, compound 4f showed comparatively higher cytotoxicity, with an IC_50_ value of 79.75 ± 2.3 µM, although this value still indicates moderate toxicity relative to many biologically active heterocyclic compounds. The cytotoxicity assessment against normal human fibroblasts provides only a preliminary indication of mammalian safety. Future studies should include comprehensive toxicological evaluations in multiple cell lines and appropriate *in vivo* models to establish the safety profile of these compounds and support their potential larvicidal activity against *Anopheles arabiensis*.

**Table 7:** IC_50_ values (µM) of the synthesized compounds against a normal fibroblast cell line^a^

Compound No	IC_50_ (µM)
14a	>200
14b	>200
14c	>200
14d	>200
14e	157.33 ± 2.66
14f	79.75 ± 2.34
14g	173.13 ± 1.26
14h	114.43 ± 4.14

a Data presented as mean and standard deviation. IC_50_ values are the mean of triplicate of three independent experiments.

### 3.11. Molecular docking studies

**Fig. 11:**
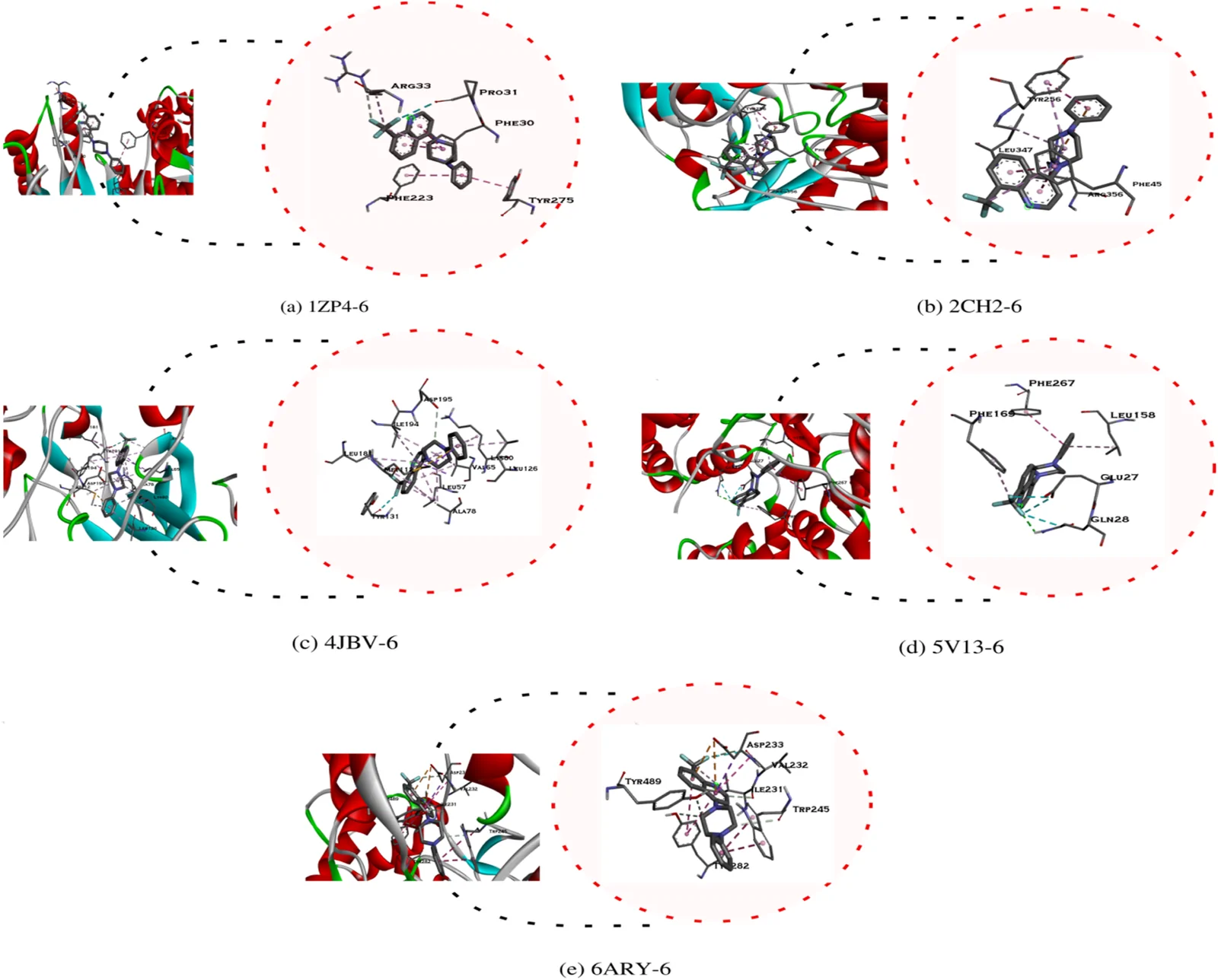
Molecular docking simulations of ligand–protein interactions for the compound 4b.

**Fig. 12:**
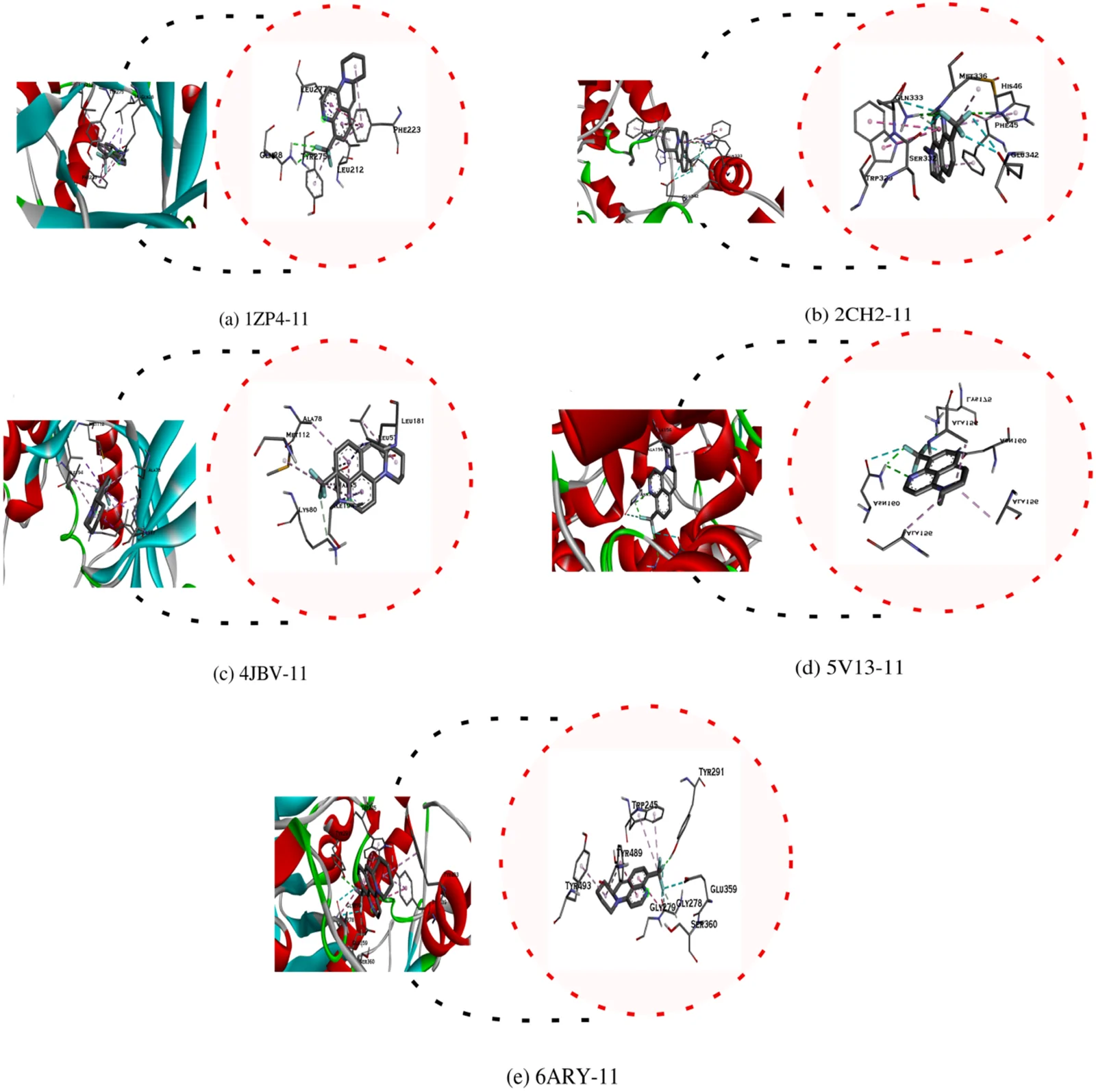
Molecular docking simulations of ligand–protein interactions for the compound 4g.

**Fig. 13:**
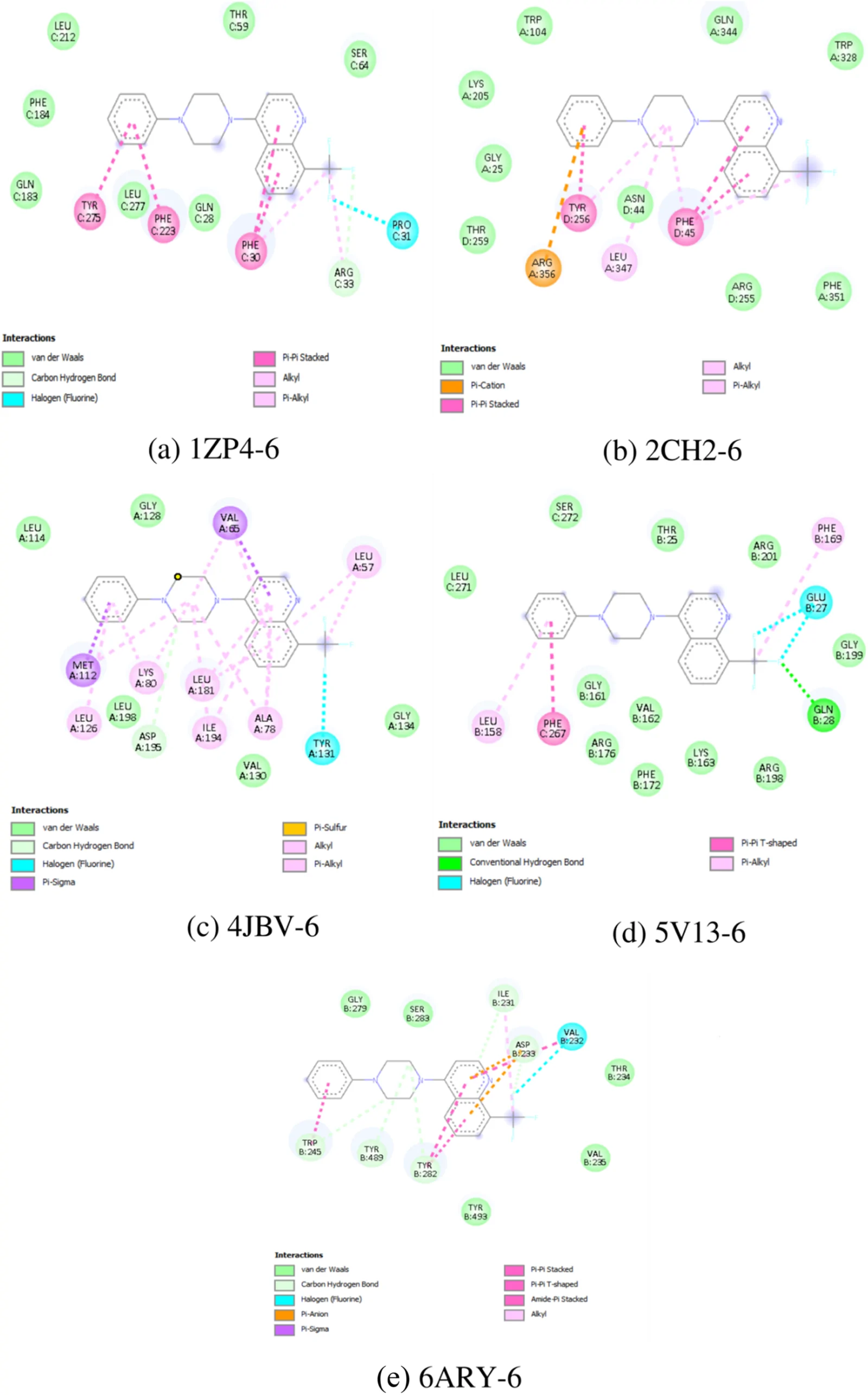
2D interactions for compound 4b with (a) PDB ID: 1ZP4-6, (b) PDB ID: 2CH2-6, (c) PDB ID: 4JBV-6, (d) PDB ID: 5V13-6, and (e) PDB ID: 6ARY-6.

**Fig. 14:**
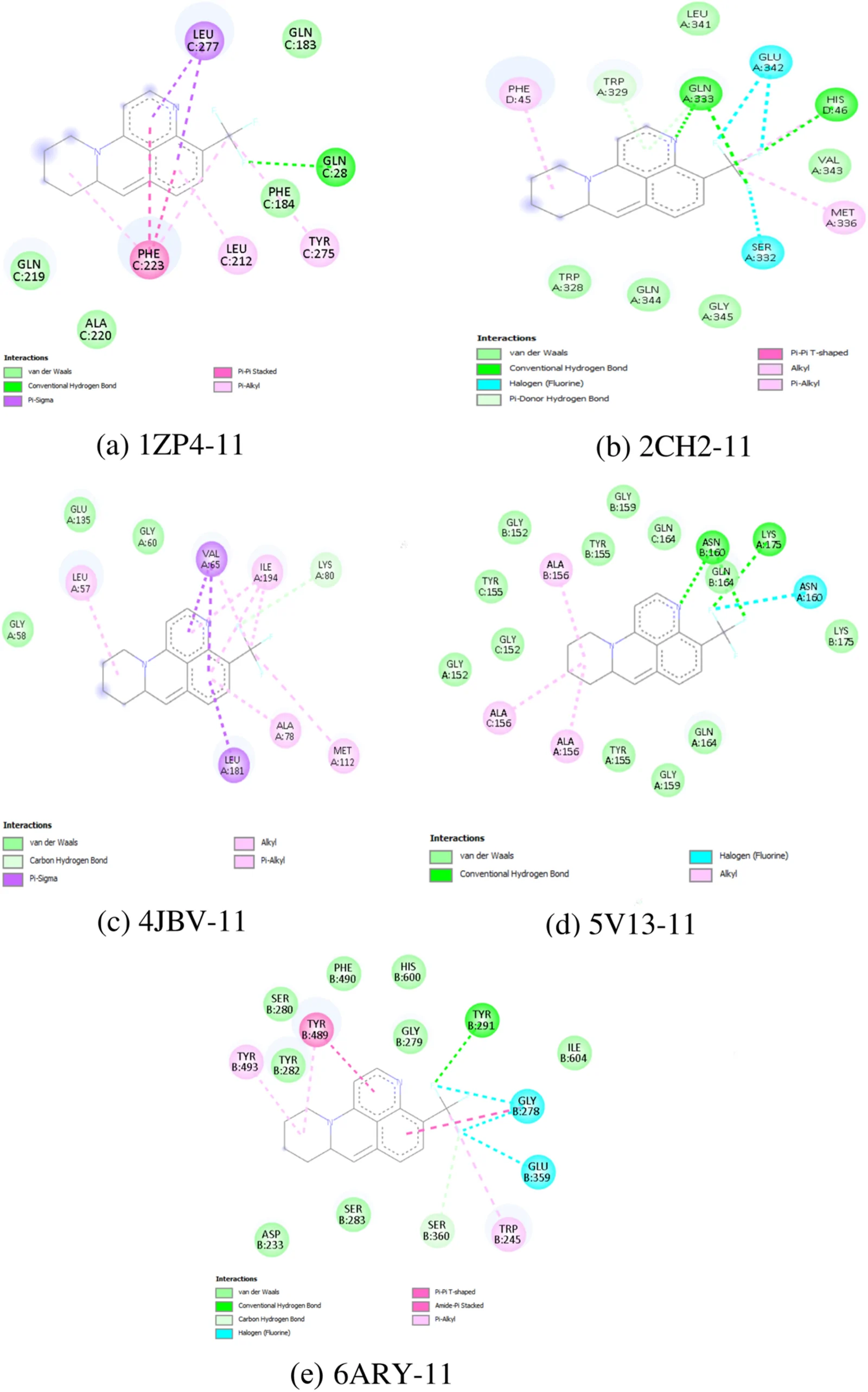
2D-interactions for compound 4g with (a) PDB ID: 1ZP4-11, (b) PDB ID: 2CH2-11, (c) PDB ID: 4JBV-11, (d) PDB ID: 5V13-11, and (e) PDB ID: 6ARY-11.

Since the designed title compounds 4a–4h show significant contribution towards larvicidal activities, molecular docking simulations were conducted to study their potential molecular targets and elucidate the probable molecular mechanism. Further, for the compound 4b and 4g it is found that the binding affinity against ligand-protein complexes shows that (a) PDB ID: 1ZP4, (b) PDB ID: 2CH2, (c) PDB ID: 4JBV, (d) PDB ID: 5V13, and (e) PDB ID: 6ARY are investigated towards their binding score against malaria vector as summarized in the Tables 8 and 9, respectively. All proteins showed significant binding scores and higher biological activity. The ligands with compound codes 4b and 4g are promising candidates with higher binding scores. Fig. 11 and 12 show the 3D-protein-ligand molecular docking simulations with protein-ligand complexes for compounds 4b and 4g. Fig. 13 and 14 show the 2D-protein-ligand interactions for compounds 4b and 4g, (a) PDB ID: 1ZP4, (b) PDB ID: 2CH2, (c) PDB ID: 4JBV, (d) PDB ID: 5V13, and (e) PDB ID: 6ARY complexes.

**Table 8:** Binding score, binding interactions and amino acid residue details for the ligand compound 4b against malaria vector

PDB:ID	Binding score (kcal mol^−1^)	Binding interactions	Amino acids
1ZP4	−9.0	Carbon hydrogen bond, halogen (fluorine), Pi–Pi stacked, alkyl, Pi–alkyl	ARG33, PRO31, PHE30, PHE223, TYR275
2CH2	−9.3	Pi-cation, Pi–Pi stacked, alkyl, Pi–alkyl	ARG356, PHE45, TYR256, LEU347
4JBV	−9.5	Carbon hydrogen bond, halogen (fluorine), Pi-sulphur, Pi-sigma, alkyl, Pi–alkyl	ASP195, TYR131, MET112, VAL65, LEU57, ILE194, ALA78, LYS80, LEU181, LEU126
5V13	−8.4	Conventional hydrogen bond, halogen (fluorine), Pi–alkyl, Pi–Pi T-shaped	GLN28, GLU27, PHE267, LEU158, PHE169
6ARY	−10.0	Conventional hydrogen bond, halogen (fluorine), Pi–Pi stacked, Pi-anion, Pi-sigma, Pi–Pi T-shaped	ILE231, TRP245, TYR282, TYR489, ASP233, VAL232

**Table 9:** Binding score, binding interactions and amino acid residue details for the ligand compound 4g against malaria vector

PDB:ID	Binding score (kcal mol^−1^)	Binding interactions	Amino acids
1ZP4	−9.1	Conventional hydrogen bond, halogen (fluorine), carbon hydrogen bond, Pi–alkyl	GLN28, LEU277, PHE223, LEU212, TYR275
2CH2	−8.1	Conventional hydrogen bond, halogen (fluorine), Pi-cation, Pi-donor hydrogen bond, Pi–Pi T-shaped, Pi–Pi stacked, alkyl	GLN333, HIS46, SER332, GLU342, TRP329, MET336, PHE45, HIS46
4JBV	−8.6	Carbon hydrogen bond, Pi-sigma, alkyl, Pi–alkyl	LYS80, VAL65, LEU181, MET112, ILE194, LEU57, ALA78
5V13	−9.6	Conventional hydrogen bond, hydrogen bond, halogen (fluorine), alkyl	LYS175, ASN160, ALA156
6ARY	−9.0	Conventional hydrogen bond, halogen (fluorine), Pi–Pi T-shaped, Pi–alkyl, amide-Pi stacked	TYR291, SER360, GLY278, GLU359, TYR489, TRP245

#### 3.11.1 Binding interaction analysis against active site 1ZP4

All the compounds 4a–4h showed binding scores ranging between 8.3 and 9.1 kcal mol^−1^, as shown in Table S6. The hydrogen-bond interactions were established between the ligands and the amino acid residues of LEU277, PHE233, TYR275, and PHE184. These hydrogen bonds help in stabilizing the ligands within the binding site, which further enhances their overall binding affinity with the receptor. These interactions might enhance the ligand stability and affinity for the binding site. Halogen bonding can be observed for all eight ligands 4a to 4h with GLN219, PRO31, ALA220, ARG197, and GLN28, which may lead to enhancing the binding affinity of the ligands. It is also observed that various hydrophobic interactions with active amino acid residues serve to fortify and stabilize the protein structure. π–π stacking interactions can also be observed for the ligand with PHE223 and PHE30, which helps in structural stabilization of the ligand-receptor complexes. Compound 4-(4-phenylpiperazin-1-yl)-8-(trifluoromethyl)quinoline 4b was found to be established with hydrogen-bonding interactions, halogen bonding, and π–π stacking, and also shows a good binding score of −9.0 kcal mol^−1^, signifying the compound to be the most biologically active molecule. Fig. S25 shows 2D ligand-protein interaction of 1ZP4-complexes.

#### 3.11.2 Binding interaction analysis against active site 2CH2

The binding score for the reported ligands 4a–4h ranged between −7.6 kcal mol^−1^ to −9.3 kcal mol^−1^ as shown in Table S7. The 2D ligand-protein interaction of 2CH2-complexes is shown in Fig. S26. The binding score for the ligand 4b is found to be −9.3 kcal mol^−1^, signifying strong electrostatic interactions with ARG356. Pi-cation, Pi–Pi stacked, alkyl, and Pi–alkyl interactions are observed. Hydrophobic interactions are observed with amino acid residues PHE45, TYR256, and LEU347, which are responsible for stabilizing the protein and enhancing the overall affinity of the protein. Test compounds 4a–4h also exhibit good binding affinity, having conventional hydrogen bonding and carbon-hydrogen bonding. π–π interactions are observed for all the reported nine ligands. It is observed that ligand 4b is formed by π–π interactions with the amino acid PHE 45 and the quinolone ring of the molecule.

#### 3.11.3 Binding interaction analysis against active site 4JBV

All the eight ligands found to exhibit carbon hydrogen bond, halogen (fluorine), π–sulfur, π–sigma, alkyl, π–alkyl interactions with the protein. For ligand 4b strong hydrogen bond interactions are observed between amino acid ASP195 and phenyl-piperazine group of the molecule enhancing the overall binding affinity and stabilizing the ligand. Hydrophobic interactions are observed between the amino acids VAL65 and phenyl-piperazine, ALA 78, LEU 181, LEU126, TYR 131, LEU57 and phenyl-piperazine and quinolone rings of the molecule. These interactions enhance the binding affinity and stabilize the protein. Because of these strong interactions ligand 4b is found to have a high binding score of −9.5 kcal mol^−1^. Table S8 shows the detailed binding score, interactions, and amino acids, whereas Fig. S27 Shows the 2D ligand-protein interaction of 4JBV complexes.

#### 3.11.4 Binding interaction analysis against active site 5V13

From compounds 4a–4h, compound 4g exhibits the highest binding score of −9.6 kcal mol^−1^, as shown in Table S9. This may be due to the hydrogen bonding between the amino acid residue ASN160 and the piperidine group of a molecule. The protein-ligand interactions show that hydrophobic interactions also exist between the residues ALA156 and the quinolone ring in a molecule. All other ligands exhibit conventional hydrogen bonding and hydrophobic interactions. Compound 4b has a binding score of −8.4 kcal mol^−1^, which is less compared to other ligands, when compared to other proteins reported. Fig. S28, shows the 2D ligand–protein interaction of 5V13-complexes.

#### 3.11.5 Binding interaction analysis against active site 6ARY

The designed ligands 4a–4h exhibited a binding score between −7.9 kcal mol^−1^ to −10.0 kcal mol^−1^ is shown in Table S10. The hydrogen-bond interactions were established between the ligands and the amino acid residues TYR282, PHE490, TRP245, TYR282, TYR489, ASP233 and SER283. These hydrogen bonds stabilize the ligands within the binding site and further enhance their overall affinity of the protein. Halogen bond is observed for all the eight ligands with VAL232, TYR489, TRP245, GLY278 and, TRP245, which may lead to enhanced binding affinity with the ligands. It is seen that various hydrophobic interactions with active amino acids residues may further stabilize the ligand-protein interactions. π–π stacking interactions are observed for TRP245, TRP441, TYR493 and TYR282, these may lead to enhance the binding affinity and stabilizing the ligand–receptor complex. Fig. S29 shows 2D-interactions for ligand-protein (6ARY-complexes).

### 3.12. *In silico* ADMET studies

**Fig. 15:**
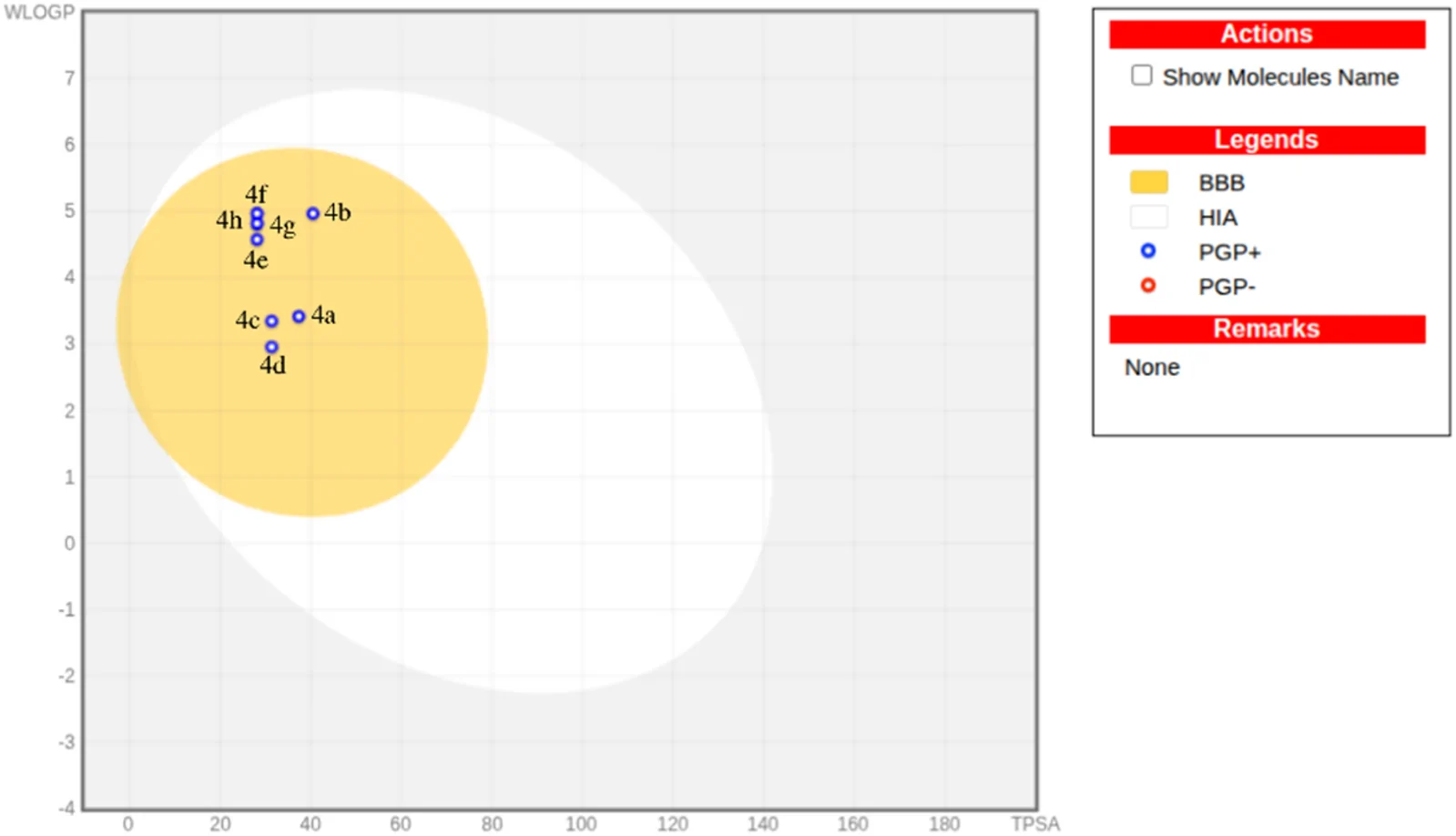
BOILED Egg models for the compounds 4a–4h.

The ADMET properties were employed using pkCSM and SWISS ADME online tools. The results pertaining to ADMET profile prediction are shown in Table 10. The human intestinal absorption value is greater than 90%, resulting in a good absorbance for all eight compounds 4a–4h. The molar refractivity values range between 72.5 to 106.21, which are in the standard range of 30 to 140 indicating that they have favorable molecular polarity. Lipophilicity is assessed using iLOGP and SILICOS-IT, and the values for all eight compounds were in the acceptable range. Values for water solubility, which is very crucial for absorption and distribution, are in the range −3.791 to −5.579 log mol L^−1^ for all the designed compounds (4a–4h), indicating they are completely soluble. All compounds show that the blood–brain barrier (BBB) permeability remains positive. Evaluation of all eight compounds (4a–4h) reveals that P450 (CYP) enzyme inhibition is observed to be negative. Drug-likeness rules indicate that all the designed compounds complied with Lipinski, Ghose, Egan, and Muegge scores, as well as Bioavailability Scores. Medicinal chemistry results are assessed using the PAINS and Brenk filters, which confirm that all compounds 4a–4h have no significant issues (Table S4). The total clearance values are within the acceptable range of 0.146 to 0.517 mL min^−1^ kg^−1^, indicating how quickly drugs are excreted from the human body. The BOILED-Egg model (Fig. 15) predicts that the reported compounds 4a–4h lie in the yellow zone of the BBB, predicting that all the compounds successfully cross the BBB. The blue spot indicated that compounds 4a–4h are likely to be excreted from P-glycoprotein (PGP+), whereas compound 4g is excreted from P-glycoprotein (PGP-).

**Table 10:** Tabular presentation of *in silico* ADMET parameters for all compounds 4a–4h under study

Model name	Units	Compounds codes							
		4a	4b	4c	4d	4e	4f	4g	4h
AMES toxicity	Yes/no	No	Yes	No	No	No	No	No	No
Max. Tolerated dose (human)	log mg kg^−1^ day^−1^	−0.25	−0.557	−0.298	−0.295	−0.295	−0.056	−0.125	0.004
hERG I inhibitor	Yes/no	No	No	No	No	No	No	No	No
hERG II inhibitor	Yes/no	No	Yes	No	No	No	No	No	No
Oral rat acute toxicity (LD_50_)	mol kg^−1^	2.563	2.335	2.903	2.851	2.605	2.627	2.715	2.588
Oral rat chronic toxicity (LOAEL)	log mg^−1^ kg^−1^ bw per day	1.333	1.244	1.298	1.388	1.047	1.053	1.007	0.963
Hepatotoxicity	Yes/no	Yes	No	Yes	Yes	Yes	Yes	Yes	Yes
Skin sensitisation	Yes/no	No	No	No	No	No	No	No	No
*T.Pyriformis* toxicity	log µg L^−1^	1.763	1.448	1.629	1.594	2.171	1.798	1.733	1.955
Minnow toxicity	log mM	1.496	0.485	2.051	2.216	1.156	0.139	0.266	−0.042

### 3.13. Toxicity studies

Various toxicity properties of the designed compounds 4a–4h were predicted in *silico* using the pkCSM tool and the SWISS ADME tool, as detailed in Table 10. A broad spectrum of toxicities is covered, including cardiotoxicity, hepatotoxicity, sensitization, nephrotoxicity, acute toxicity, and carcinogenicity.

Potential carcinogenicity predictions are very crucial in ensuring medication for public safety. Mutagenicity was assessed using the Ames test, with all compounds predicted to be Ames-negative except compound 4b Predicting cardiotoxicity, hERG channel blockage plays a vital role. The designed compounds 4a–4h, except compound 4b, avoid hERG inhibition, hence reducing the risk to the cardiovascular system. In analyzing the severity of acute toxicity, quantitative measures like lethal dose (LD_50_) are employed. The LD_50_ values for the compounds 4a–4h are in the range of 2.0 to 5.0 mol kg^−1^, indicating a safe profile. The designed compounds show that the presence of hepatotoxicity can be avoided by administering the dosage levels, modifying structures, and using some protective agents. None of the eight synthesized compounds exhibits skin sensitization. The Minnow toxicity values for all eight compounds are within the safe values.

### 3.14. Structure activity relationship [SAR]

Trifluoromethyl is a strong electron-withdrawing group, so it exerts an inductive effect and reduces the basicity of the quinoline ring. As a result, hydrophobicity increases. Because CF_3_ is a bulky group, it shields the 8th position and alters the binding orientation. 8-Trifluoromethylquinoline is a weaker base than quinoline (p*K*_a_ of the conjugate acid ≈ 3.5–4.2 and p*K*_a_ of the conjugate acid ≈ 4.9).

Strong electron-donor amines (pyrrolidine, piperidine, alkyl-substituted piperidines) generally have a larger effect than weaker donors such as piperazine, *N*-methylpiperazine, *N*-ethylpiperazine, *N*-phenylpiperazine, and morpholine.

Replacing the aminoalkyl side chain with a diethylamino group or with heterocyclic rings such as piperidine, pyrrolidine, or morpholine in chloroquine derivatives substantially increases antimalarial activity against chloroquine-resistant strains.^75^

**Fig. 16:**
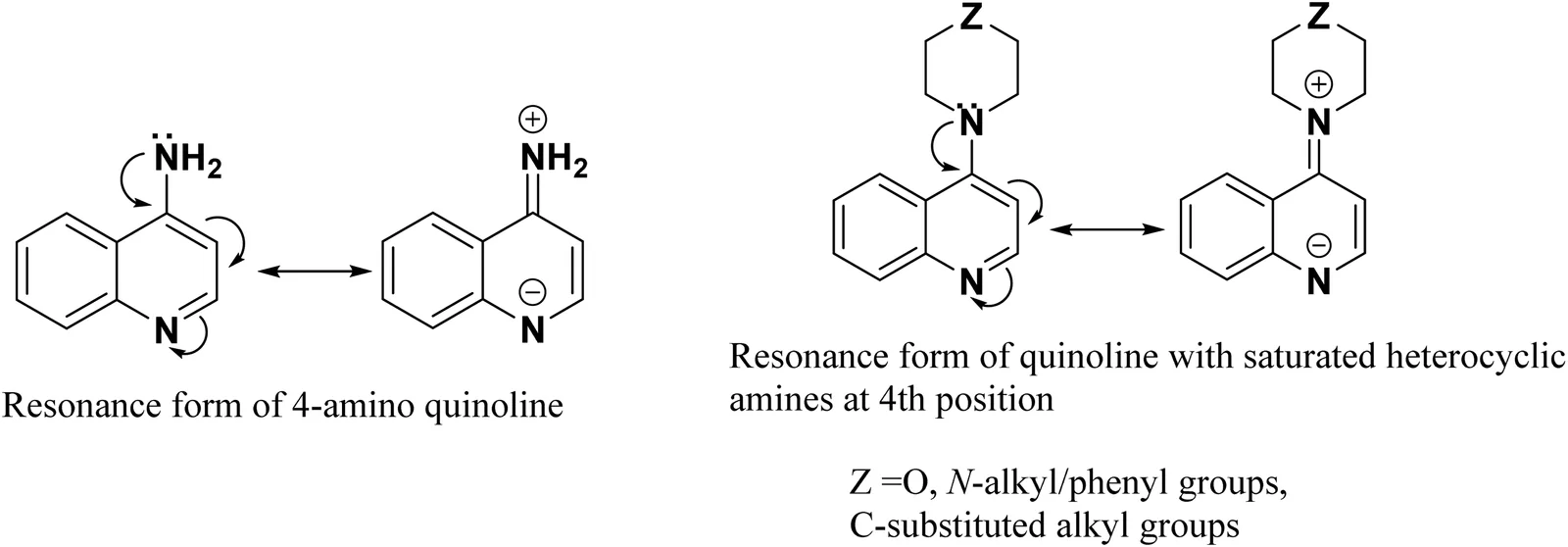
Resonance forms of 4-aminoquinolines.

It is apparent from the literature that the amino group at the C4 position of 4-aminoquinolines resonates favorably through delocalization of its lone pair. The introduction of nitrogen-containing saturated heterocyclic ring systems is expected to influence this delocalization (Fig. 16) and, therefore, the biological activity of the molecules.^76^ Compound 4b exhibited the highest larval mortality because it contains a phenyl ring attached to the piperazine ring at the N4 position, where the phenyl ring stabilizes the hydrogen-bonding interactions formed by the two nitrogens.

Although the synthesized quinoline derivatives exhibited significant larvicidal activity, the exact molecular mechanism responsible for larval mortality remains to be experimentally established. The molecular docking studies in this work were intended to identify potential biological targets rather than to demonstrate a definitive mechanism of action. The favorable binding affinities observed for the most active compounds suggest that these derivatives may interfere with proteins essential for larval survival and development. Given the broad biological activities of quinoline derivatives and the multifactorial nature of insecticidal action, the observed larvicidal activity likely results from disruption of one or more critical biochemical pathways. Further target-specific biochemical studies and molecular investigations are required to validate these computational predictions and elucidate the precise mechanism of action.

## 4. Conclusion

A novel series of 8-trifluoromethyl quinoline derivatives bearing substituted saturated amines (4a–4h) was synthesized through the reaction of 4-chloro-8-trifluoromethyl quinoline with various saturated amines, and characterized by ^1^H and ^13^C-NMR and mass spectrometry. All compounds showed larvicidal activity compared to the acetone negative control, with compound 4b being the most promising, resulting in close to 100% mortality. The compounds had favorable safety profiles when tested against normal fibroblasts. The lack of a significant time-exposure effect indicates that mortality patterns were consistent across both time points (*i.e.*, not increasing during that time frame). Quantitative dose–response analysis corroborated these observations, with compound 4b exhibiting the lowest LC_50_ values among the synthesized compounds at both 24 h (1.60 µg mL^−1^) and 48 h (1.42 µg mL^−1^), approaching the potency of the reference larvicide Temephos (RR_50_ = 1.37 and 1.41, respectively), whereas 4c and 4d required markedly higher concentrations to achieve comparable mortality (RR_50_ = 1.86–2.52) confirming 4b as the most promising compound of the series. Furthermore, the close agreement between LC_50_ estimates at 24 and 48 h for all compounds, together with the parallel slopes obtained across dose–response curves is consistent with the absence of significant time-exposure effect on larvicide activity. The SCXRD revealed that the compound crystallized in a monoclinic lattice system, with the space group *P*2_1_/*n*. In the compound, molecules are stabilized by intra-C–H⋯O), and short intermolecular (C–H⋯N and C–H⋯F) interactions, and it was explored by Hirshfeld surfaces mapped on different properties. A two-dimensional fingerprint plot reveals that H⋯H (33.2%), H⋯O (6.1%), H⋯C (12.4%), and H⋯N (5.8%), interactions make the most significant contributions to the crystal packing. DFT studies show that the optimized geometry and geometrical parameters confirm that the experimental and theoretical values agree with the reported compound 4a. HOMO–LUMO analysis confirms that compound 4b is more stable than compound 4a. MEP studies show that the compound 4b exhibits a strong electron rich region across fluorine atom compared to compound 4a. *In silico* (molecular docking simulations) studies are carried out against all the designed eight compounds 4a–4h towards the malarial vector *Anopheles arabiensis* against five proteins (PDB ID: (a) 1ZP4, (b) 2CH2, (c) 4JBV, (d) 5V13, and (e) 6ARY). Compound 4b showed a higher binding score compared to the other ligands against all five proteins, with the highest binding affinity observed for protein 6ARY (−10.0 kcal mol^−1^) compared to the other four protein complexes. The higher binding affinity stabilizes the ligand-receptor complexes and enhances biological activity against all proteins. ADMET results show satisfactory toxicity properties. Further, these findings identify compound 4b as a promising lead for further development and optimisation as a potential larvicidal agent for mosquito vector control.

## Author contributions

Conceptualization, S. K.; B. N. L.; L. A. D.; S. N. C.; A. V. M.; R. M. G.; A. K. A.; N. P.; R. J.; K. N. V.; and J. P. D.; methodology, S. K.; B. N. L.; L. A. D.; K. K.; S. N. C.; A. V. M.; R. M. G.; A. K. A.; N. P.; P. K. D; R. J.; K. N. V.; and J. P. D.; software, S. K.; B. N. L.; L. A. D.; S. N. C.; A. V. M.; R. M. G.; A. K. A.; N. P.; P. K. D; R. J.; K. N. V.; and J. P. D.; validation, S. K.; B. N. L.; L. A. D.; S. N. C.; A. V. M.; R. M. G.; A. K. A.; N. P.; P. K. D; R. J.; K. N. V.; and J. P. D.; formal analysis, S. K.; B. N. L.; L. A. D.; K. K.; S. N. C.; A. V. M.; R. M. G.; A. K. A.; N. P.; P. K. D; K. N. V.; and J. P. D.; investigation, S. K.; B. N. L.; L. A. D.; S. N. C.; A. V. M.; R. M. G.; A. K. A.; N. P.; P. K. D; R. J.; K. N. V.; and J. P. D.; resources, S. K.; B. N. L.; L. A. D.; K. K.; S. N. C.; A. V. M.; R. M. G.; A. K. A.; N. P.; P. K. D; R. J.; K. N. V.; and J. P. D.; data curation, S. K.; B. N. L.; N. P.; P. K. D; K. N. V.; and J. P. D.; writing – original draft preparation, S. K.; B. N. L.; L. A. D.; K. K.; S. N. C.; A. V. M.; R. M. G.; A. K. A.; N. P.; P. K. D; R. J.; K. N. V.; and J. P. D.; writing – review and editing, S. K.; B. N. L.; L. A. D.; K. K.; S. N. C.; A. V. M.; R. M. G.; A. K. A.; N. P.; P. K. D; R. J.; K. N. V.; and J. P. D.; visualization, B. N. L.; N. P.; P. K. D; K. N. V.; and J. P. D.; supervision, K. N. V.; and J. P. D.; project administration, K. N. V.; and J. P. D.; funding acquisition, K. N. V.; and J. P. D.

## Conflicts of interest

The authors declare no conflicts of interest.

## Supplementary Material

RA-016-D6RA05130H-s001

RA-016-D6RA05130H-s002

## Data Availability

CCDC 2202316 contains the supplementary crystallographic data for this paper.^77^ Supplementary information (SI): copies of ^1^H-NMR, ^13^C-NMR, and mass spectra of the title compounds 4a–h are available in the SI. See DOI: https://doi.org/10.1039/d6ra05130h.
