# Supplementary material for: Synthesis, characterization, crystallography insights, and larvicidal activity of 4-substituted-8-(trifluoromethyl)quinoline derivatives against Anopheles arabiensis
Source: RSC Adv. 2026 Aug 27;16(42):50867–87. doi: 10.1039/d6ra05130h (PMC13520969; doi:10.1039/d6ra05130h)
Supplement: Supplementary file 1 [file RA-016-D6RA05130H-s001.pdf]

Supplementary Information (SI) for RSC Advances.
This journal is © The Royal Society of Chemistry 2026

Synthesis, Characterization, Crystallography Insights, and Larvicidal Activity of 4-Substituted-8-(Trifluoromethyl)quinoline Derivatives against *Anopheles arabiensis*

Sukumar Kotyan[1], Banavase N. Lakshminarayana[2*], Lina A. Dahabiyeh[3], Karthik Kumara[4], Shankaranahalli N. Chandana[5], Arya Viswanath M[5], Raquel M. Gleiser[6], Abdulaziz Khalid Al Mouslem[7], Nefisath Pandikatte[8], Pran Kishore Deb[9], Ravikumar Jimmidi[10], Katharigatta N. Venugopala[7,11*], Jagadeesh Prasad Dasappa[1*]

[1] Department of Chemistry, Mangalore University, Mangalagangotri 574199, Karnataka, India; sukumarkotyan8793@gmail.com (S.K.); jprasad2003@gmail.com (J.P.D.)

[2] Research Center, Department of Physics, Adichunchanagiri Institute of Technology, Affiliated to Visvesvaraya Technological University, Jyothinagara, Chikkamagaluru 577102, Karnataka, India; bnlphysics@gmail.com (B.N.L.)

[3] Department of Pharmaceutical Sciences, School of Pharmacy, The University of Jordan, Amman, Jordan L.Dahabiyeh@ju.edu.jo (L.A.D.)

[4]Department of Physics & BSN Centre for Nano-Materials and Displays, B.M.S College of Engineering, Bull Temple Road, Bangalore, 560019, India; kk.phy21@gmail.com (K.K.)

[5]Department of Physics, Rajeev Institute of Technology, Hassan 573201, Karnataka, India; snc.rit@gmail.com

[6]CREAN-IMBIV (UNC-CONICET), Av. Valparaiso s.n., and FCEFyN, Universidad Nacional de Cordoba, Av. V. Sarsfield 299, Cordoba 5000, Argentina; raquel.gleiser@unc.edu.ar (R.M.G.)

[7] Department of Pharmaceutical Sciences, College of Clinical Pharmacy, King Faisal University, Al-Ahsa 31982, Saudi Arabia; aalmoslem@kfu.edu.sa (A.K.A.)

[8] Department of PG Studies and Research in Chemistry, Sri Dharmasthala Manjunatheshwara

College (Autonomous), Ujire 574240, Karnataka, India; nafeesath@sdmcujire.in (N.P.)

[9] Department of Pharmaceutical Sciences and Technology, Birla Institute of Technology (BIT),

Mesra 835215, Jharkhand, India; prankishoredeb@bitmesra.ac.in

[10] Center for Drug Discovery, Department of Pathology & Immunology, Baylor College of Medicine, Houston, TX 77030, USA; RAVIKUMAR.JIMMIDI@bcm.edu (R.J.)

[11] Department of Biotechnology and Food Science, Faculty of Applied Sciences, Durban University of Technology, Durban 4001, South Africa

---

* Correspondence: bnlphysics@gmail.com (B.N.L.); kvenugopala@kfu.edu.sa (K.N.V.); jprasad2003@gmail.com (J.P.D.)

TABLE OF CONTENTS

Sl No	Description	Page No.
1.	**Figure S1.** ^{1}H- NMR spectrum of 4-(8-(trifluoromethyl)quinolin-4-yl) morpholine (**4a**).	4
2.	**Figure S2.** ^{13}C-NMR spectrum of 4-(8-(trifluoromethyl)quinolin-4-yl) morpholine (**4a**).	5
3.	**Figure S3.** Mass spectrum of 4-(8-(trifluoromethyl)quinolin-4-yl) morpholine (**4a**).	6
4.	**Figure S4.** ^{1}H-NMR spectrum of 4-(4-phenylpiperazin-1-yl)-8-(trifluoromethyl) quinoline (**4b**).	7
5.	**Figure S5.** ^{13}C-NMR spectrum of 4-(4-phenylpiperazin-1-yl)-8-(trifluoromethyl) quinoline (**4b**).	8
6.	**Figure S6.** Mass spectrum of 4-(4-phenylpiperazin-1-yl)-8-(trifluoromethyl) quinoline (**4b**).	9
7.	**Figure S7.** ^{1}H- NMR spectrum of 4-(4-ethylpiperazin-1-yl)-8-(trifluoromethyl) quinoline (**4c**).	10
8.	**Figure S8.** ^{13}C-NMR spectrum of 4-(4-ethylpiperazin-1-yl)-8-(trifluoromethyl) quinoline (**4c**).	11
9.	**Figure S9.** Mass spectrum of 4-(4-ethylpiperazin-1-yl)-8-(trifluoromethyl) quinoline (**4c**).	12
10.	**Figure S10.** ^{1}H- NMR spectrum of 4-(4-methylpiperazin-1-yl)-8-(trifluoromethyl) quinoline (**4d**).	13
11.	**Figure S11.** ^{13}C-NMR spectrum of 4-(4-methylpiperazin-1-yl)-8-(trifluoromethyl) quinoline (**4d**).	14
12.	**Figure S12.** Mass spectrum of 4-(4-methylpiperazin-1-yl)-8-(trifluoromethyl) quinoline (**4d**).	15
13.	**Figure S13.** ^{1}H-NMR spectrum of 4-(piperidin-1-yl)-8-(trifluoromethyl) quinoline (**4e**).	16
14.	**Figure S14.** ^{13}C -NMR spectrum of 4-(piperidin-1-yl)-8-(trifluoromethyl) quinoline (**4e**).	17
15.	**Figure S15.** Mass spectrum of 4-(piperidin-1-yl)-8-(trifluoromethyl) quinoline (**4e**).	18
16.	**Figure S16.** ^{1}H-NMR spectrum of 4-(4-methylpiperidin-1-yl)-8-(trifluoromethyl) quinoline (**4f**).	19
17.	**Figure S17.** ^{13}C- NMR spectrum of 4-(4-methylpiperidin-1-yl)-8-(trifluoromethyl) quinoline (**4f**).	20
18.	**Figure S18.** Mass spectrum of 4-(4-methylpiperidin-1-yl)-8-(trifluoromethyl) quinoline (**4f**).	21
19.	**Figure S19.** ^{1}H- NMR spectrum of 4-(2-methylpiperidin-1-yl)-8-(trifluoromethyl)quinoline (**4g**).	22
20.	**Figure S20.** ^{13}C- NMR spectrum of 4-(2-methylpiperidin-1-yl)-8-(trifluoromethyl)quinoline (**4g**).	23
21.	**Figure S21.** Mass spectrum of 4-(2-methylpiperidin-1-yl)-8-(trifluoromethyl) quinoline (**4g**).	24

22.	**Figure S22.** ^{1}H-NMR spectrum of 4-(3,5-dimethylpiperidin-1-yl)-8-(trifluoromethyl) quinoline (**4h**).	25
23.	**Figure S23.** ^{13}C -NMR spectrum of 4-(3,5-dimethylpiperidin-1-yl)-8-(trifluoromethyl)quinoline (**4h**).	26
24.	**Figure S24.** Mass spectrum of 4-(3,5-dimethylpiperidin-1-yl)-8-(trifluoromethyl) quinoline (**4h**).	27
25.	**Table S1.** Bond lengths for compound **4a**	28
26.	**Table S2.** Bond angles for compound **4a**	29
27.	**Table S3.** Torsion angle for the compound **4a**	30
28.	**Table S4.** Tabular presentation of in silico ADME parameters for all compounds (**4a-h**) under study**.**	31-32
29.	**Table S5.** Grid Box	33
30.	**Table S6.** Molecular docking results for protein PDB ID: 1ZP4 against the malaria vector.	34
31.	**Table S7.** Molecular docking results for protein PDB ID: 2CH2 against the malaria vector.	35
32.	**Table S8.** Molecular docking results for protein PDB ID: 4JBV against the malaria vector.	36
33.	**Table S9.** Molecular docking results for protein PDB ID: 5V13 against the malaria vector.	37
34.	**Table S10.** Molecular docking PDB ID: 6ARY against the malaria vector.	38
35.	**Figure S25.** 2D ligand-protein interaction of 1ZP4-complexes	39
36.	**Figure S26.** 2D ligand-protein interaction of 2CH2-complexes	40
37.	**Figure S27.** 2D ligand-protein interaction of 4JBV complexes	41
38.	**Figure S28.** 2D ligand-protein interaction of 5V13-complexes	42
39.	**Figure S29.** Figure 2D ligand-protein interaction of 6ARY-complexes	43
40.	**Figure S30.** 2D fingerprint plot of compound **4a**.	44
41.	**Table S11.** Molecular interaction energies (k J/mol) in compound **4a.**	45
42.	**Figure S31.** Graphical visualization of the (a) coulomb energy term (Eele), (b) dispersion energy term (Edis) and, (c) total energy term (Etot) of compound 4a.	46

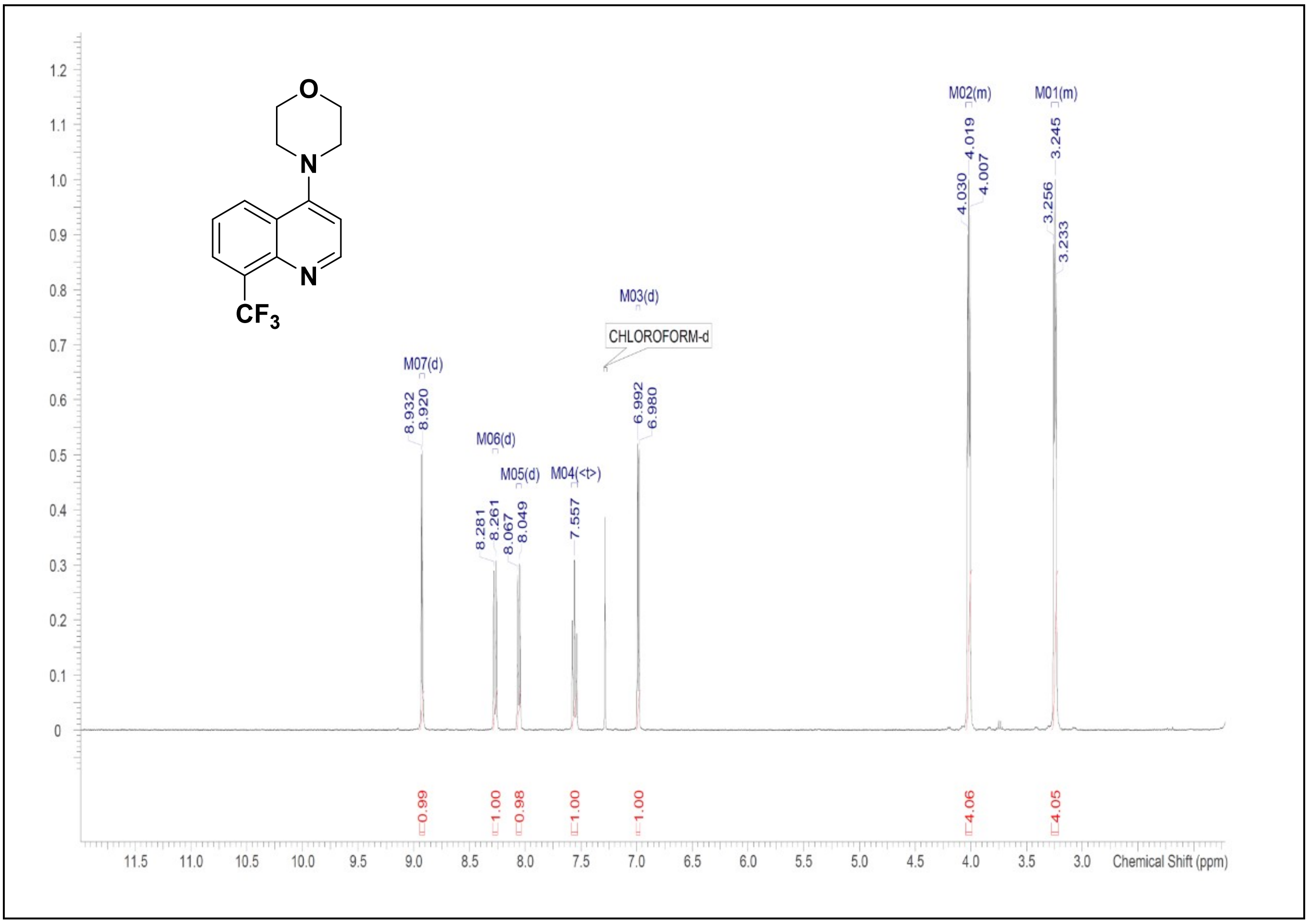

Figure S1. ^{1}H- NMR spectrum of 4-(8-(trifluoromethyl)quinolin-4-yl) morpholine (**4a**).

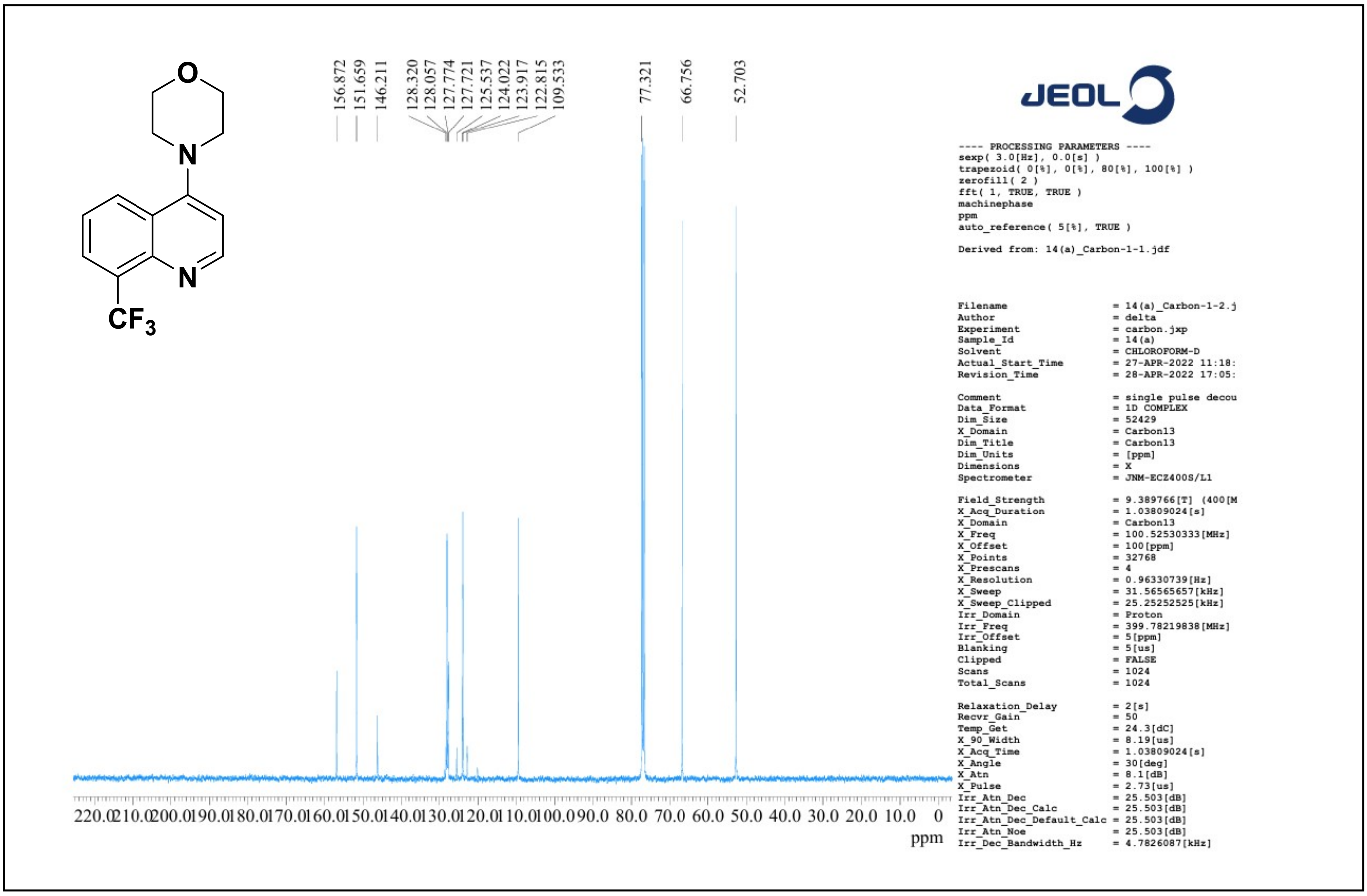

Figure S2. ^{13}C-NMR spectrum of 4-(8-(trifluoromethyl)quinolin-4-yl) morpholine (**4a**).

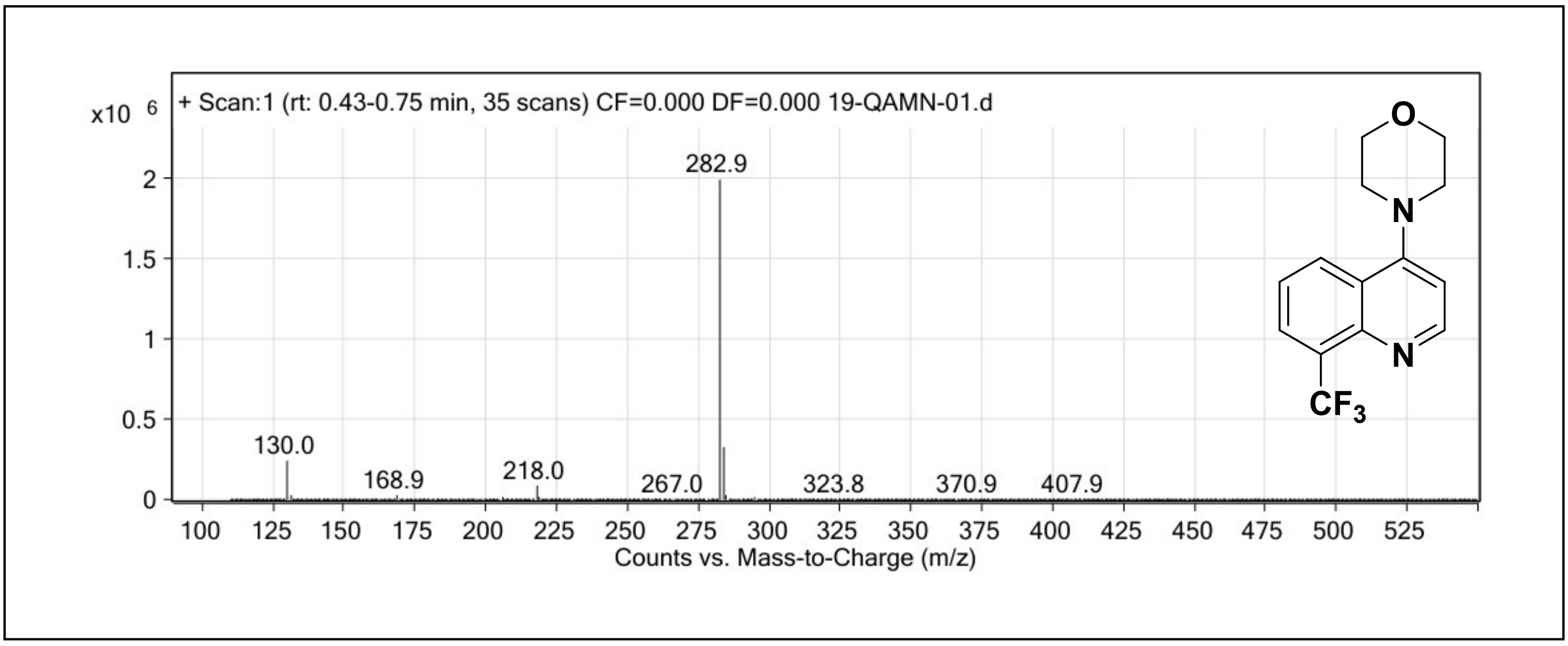

Figure S3. Mass spectrum of 4-(8-(trifluoromethyl)quinolin-4-yl) morpholine (**4a**).

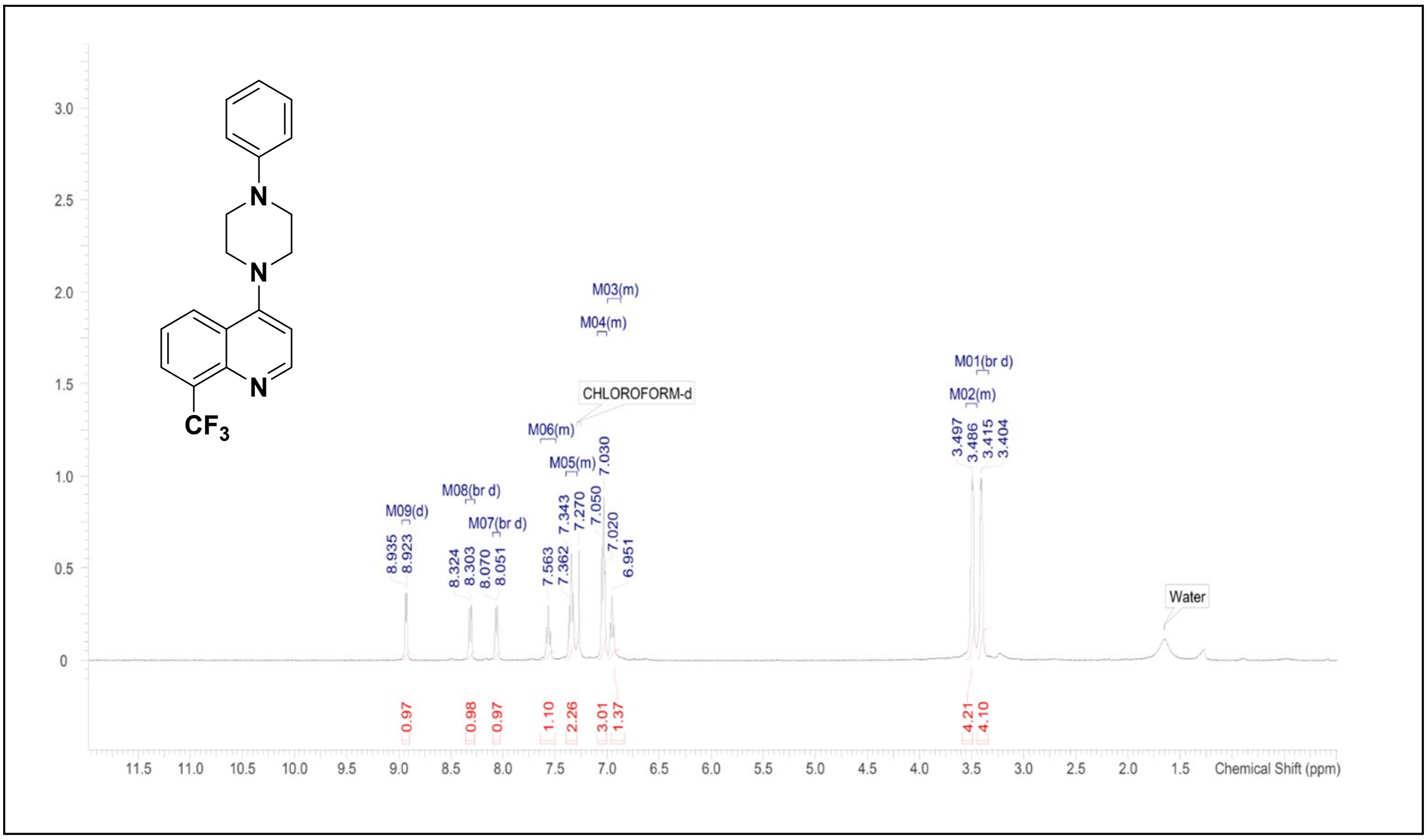

Figure S4. ^{1}H-NMR spectrum of 4-(4-phenylpiperazin-1-yl)-8-(trifluoromethyl) quinoline (**4b**).

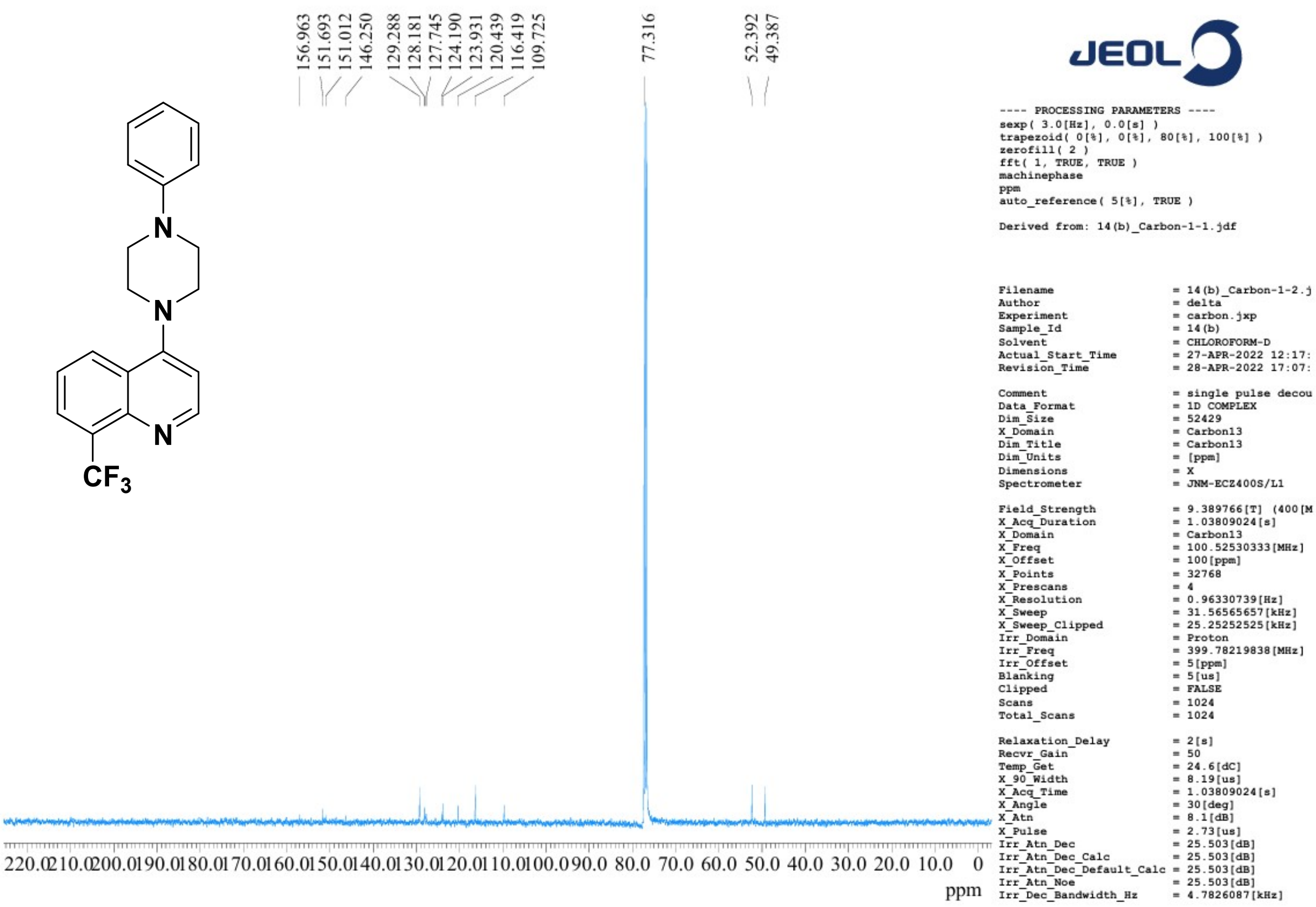

Figure S5. ^{13}C-NMR spectrum of 4-(4-phenylpiperazin-1-yl)-8-(trifluoromethyl) quinoline (**4b**).

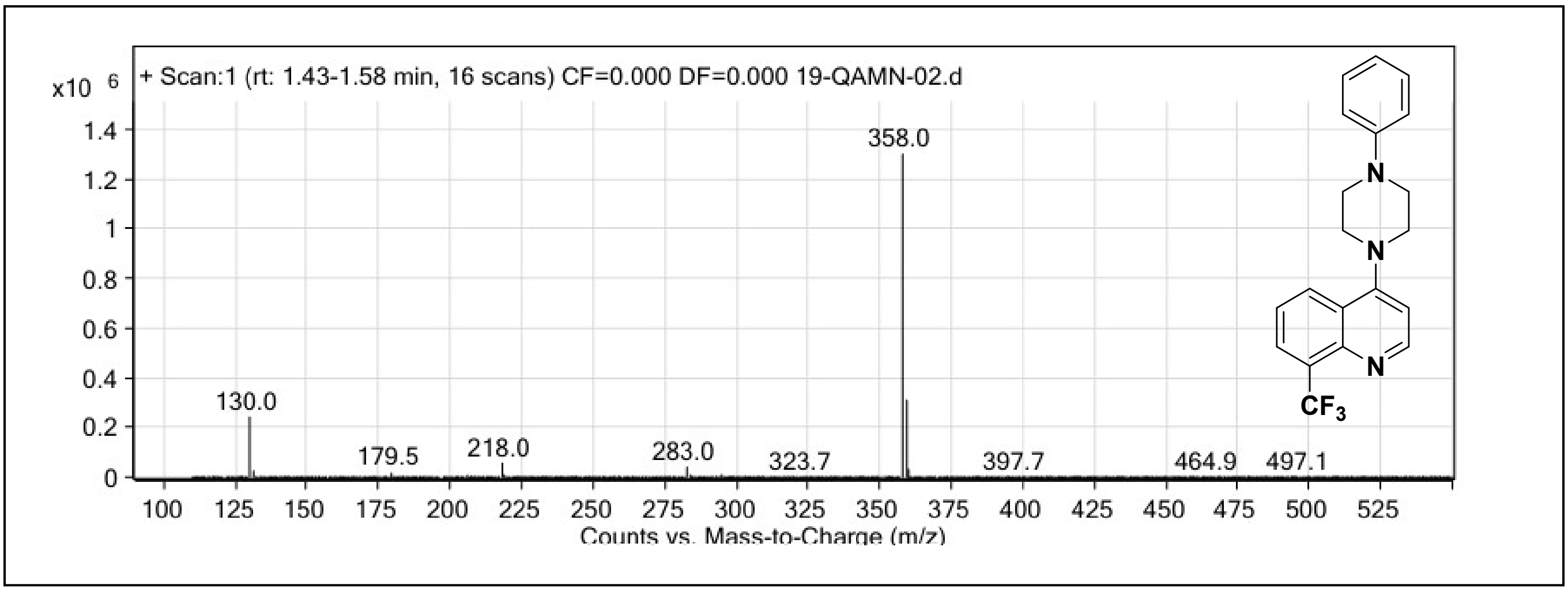

Figure S6. Mass spectrum of 4-(4-phenylpiperazin-1-yl)-8-(trifluoromethyl) quinoline (**4b**).

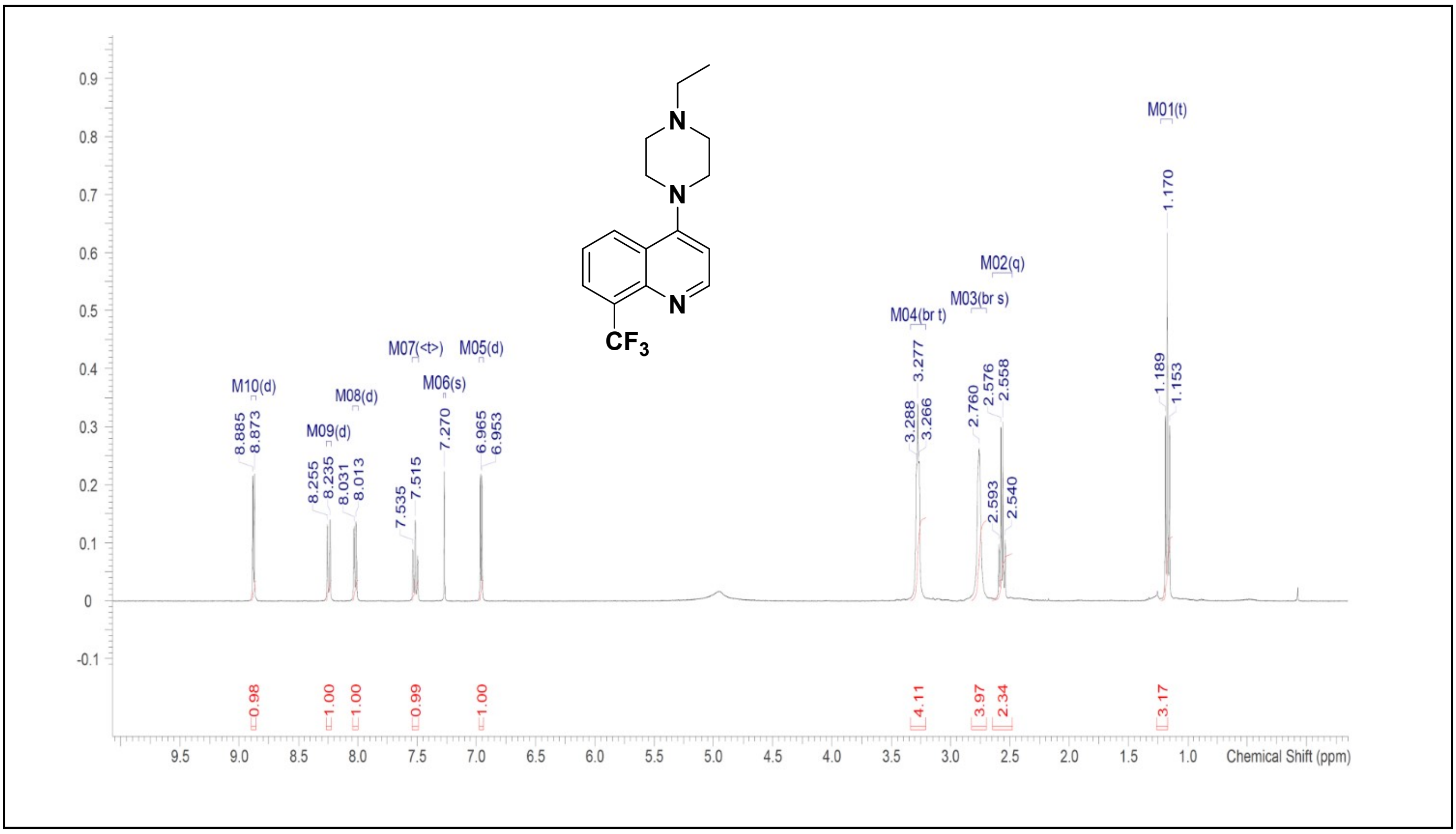

Figure S7. ^{1}H- NMR spectrum of 4-(4-ethylpiperazin-1-yl)-8-(trifluoromethyl) quinoline (**4c**).

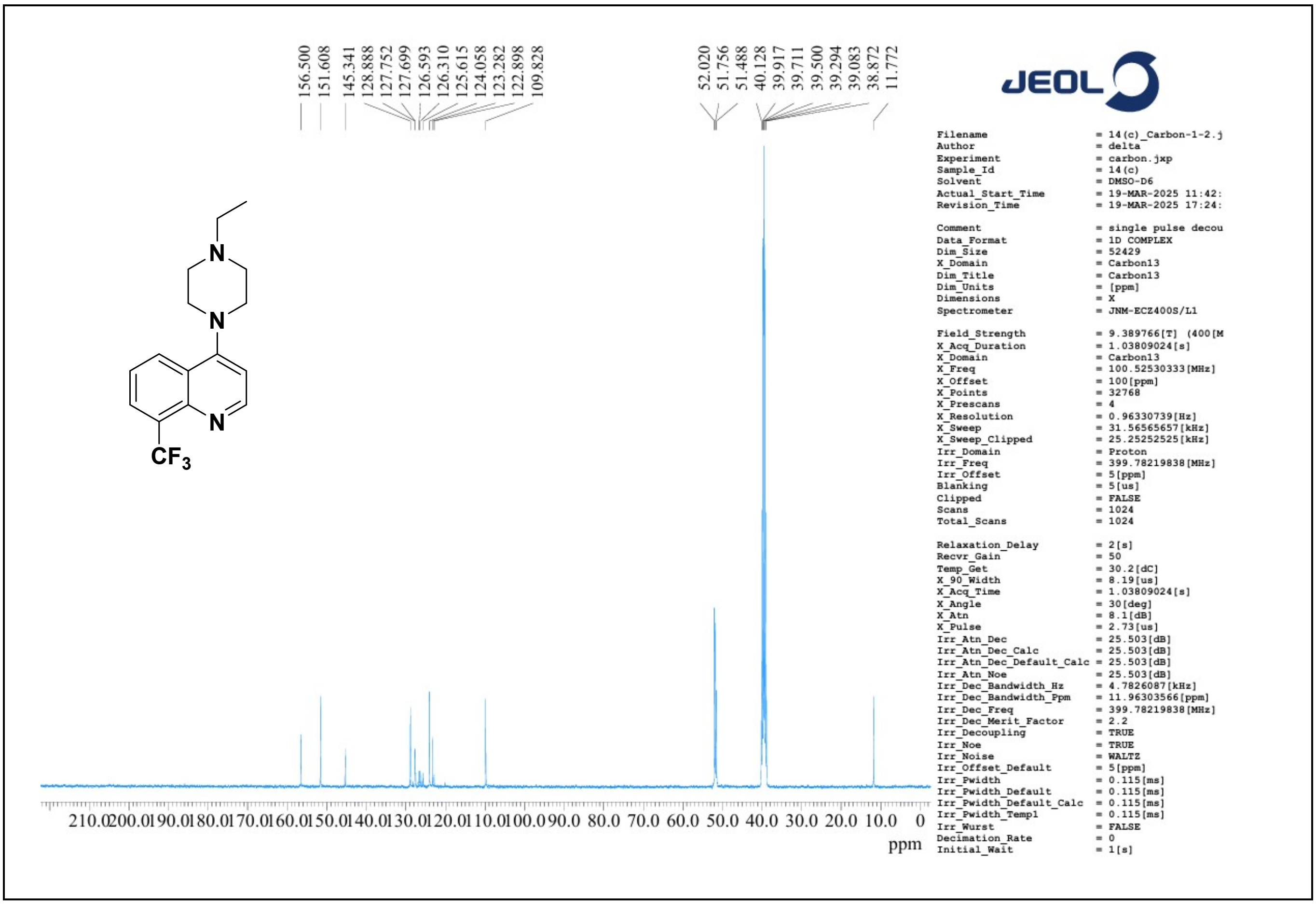

Figure S8. ^{13}C-NMR spectrum of 4-(4-ethylpiperazin-1-yl)-8-(trifluoromethyl) quinoline (**4c**).

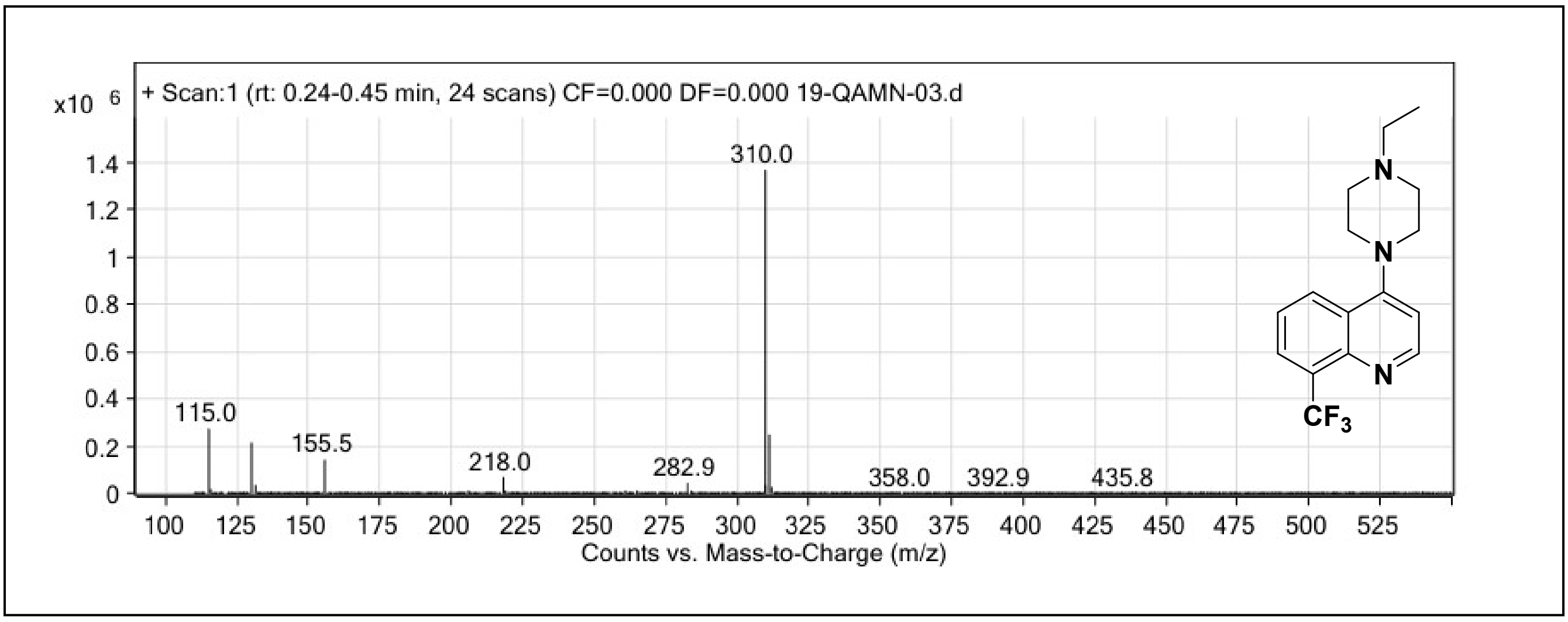

Figure S9. Mass spectrum of 4-(4-ethylpiperazin-1-yl)-8-(trifluoromethyl) quinoline (**4c**).

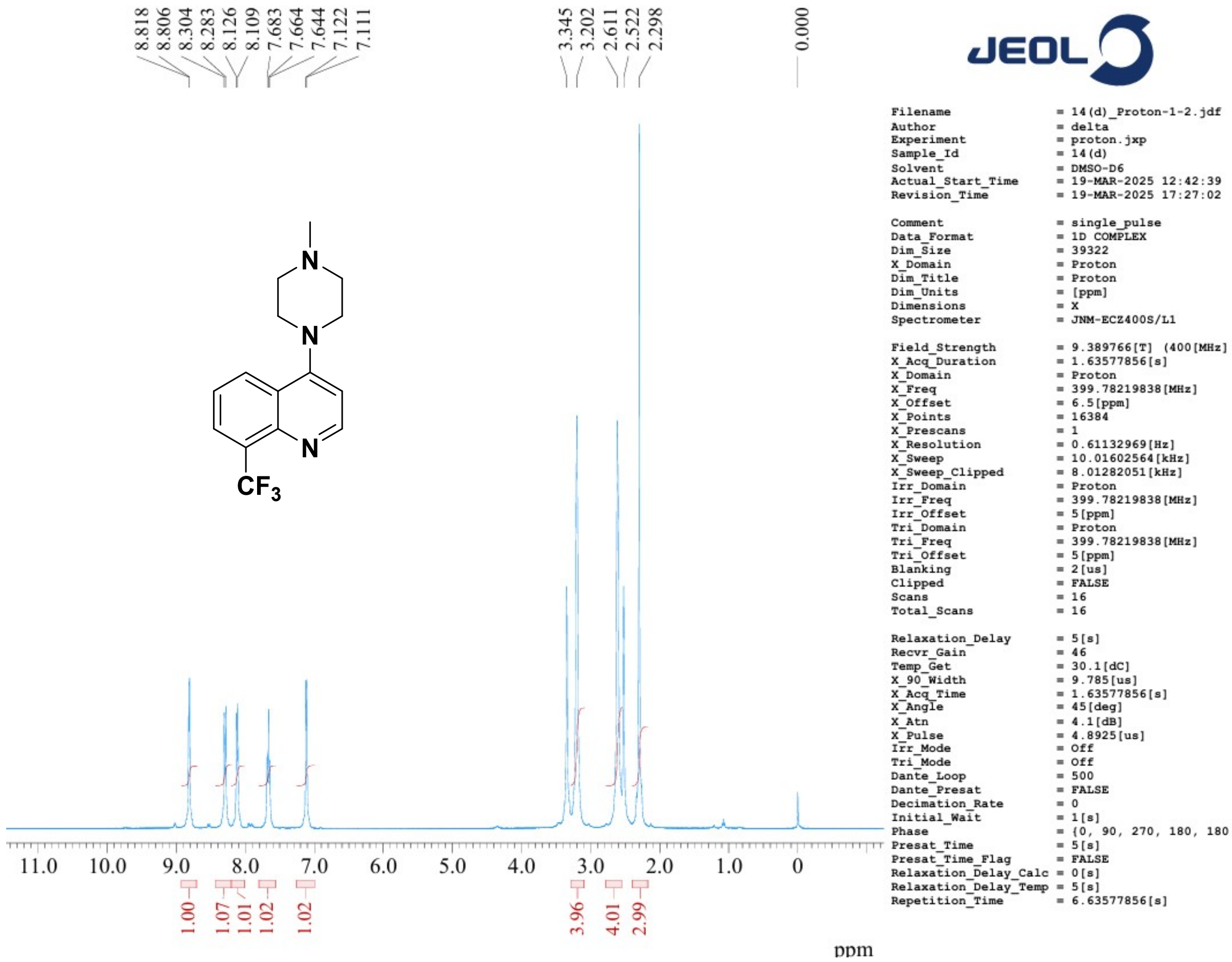

Figure S10. ^{1}H- NMR spectrum of 4-(4-methylpiperazin-1-yl)-8-(trifluoromethyl) quinoline (**4d**).

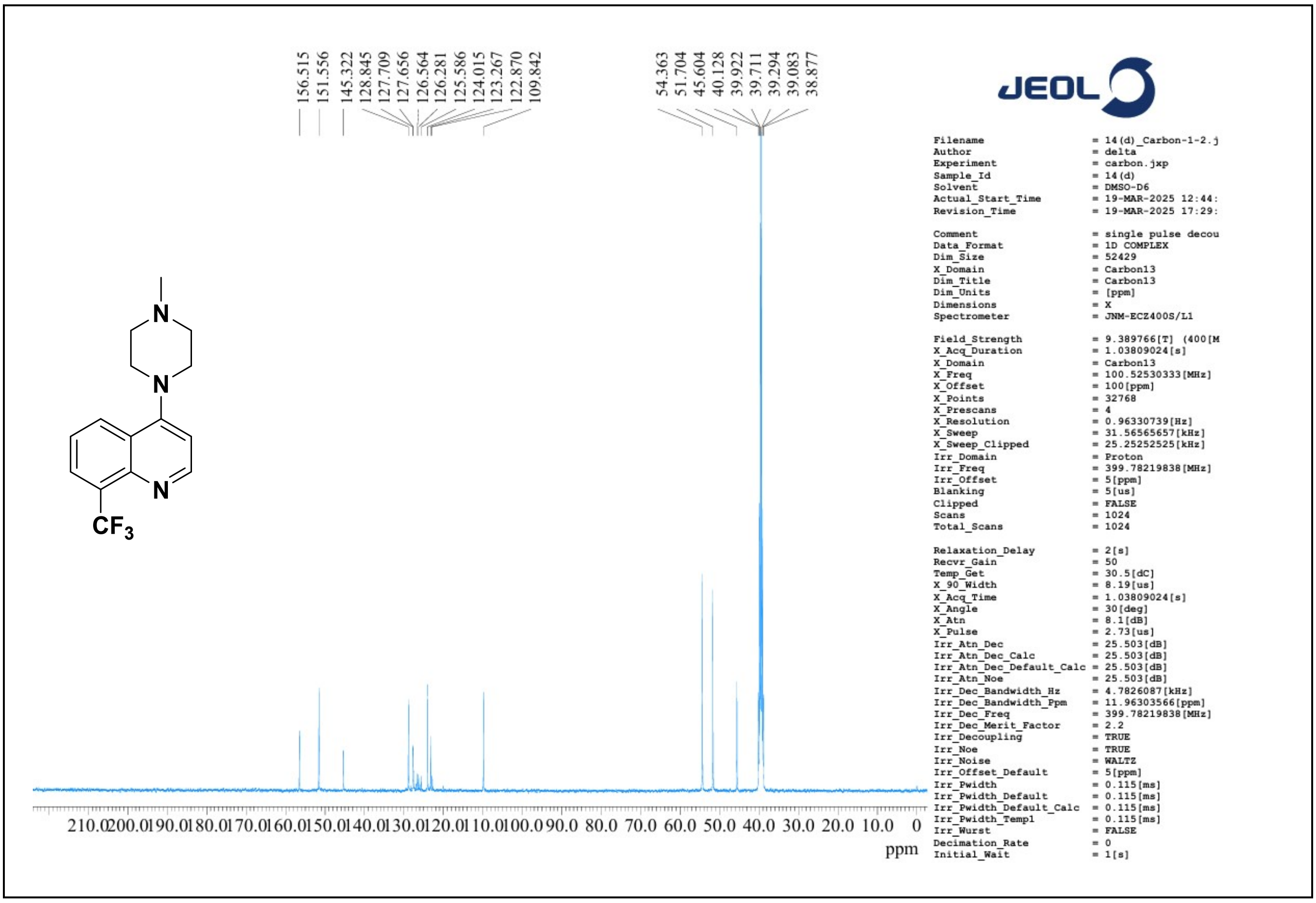

Figure S11. ^{13}C-NMR spectrum of 4-(4-methylpiperazin-1-yl)-8-(trifluoromethyl) quinoline (**4d**).

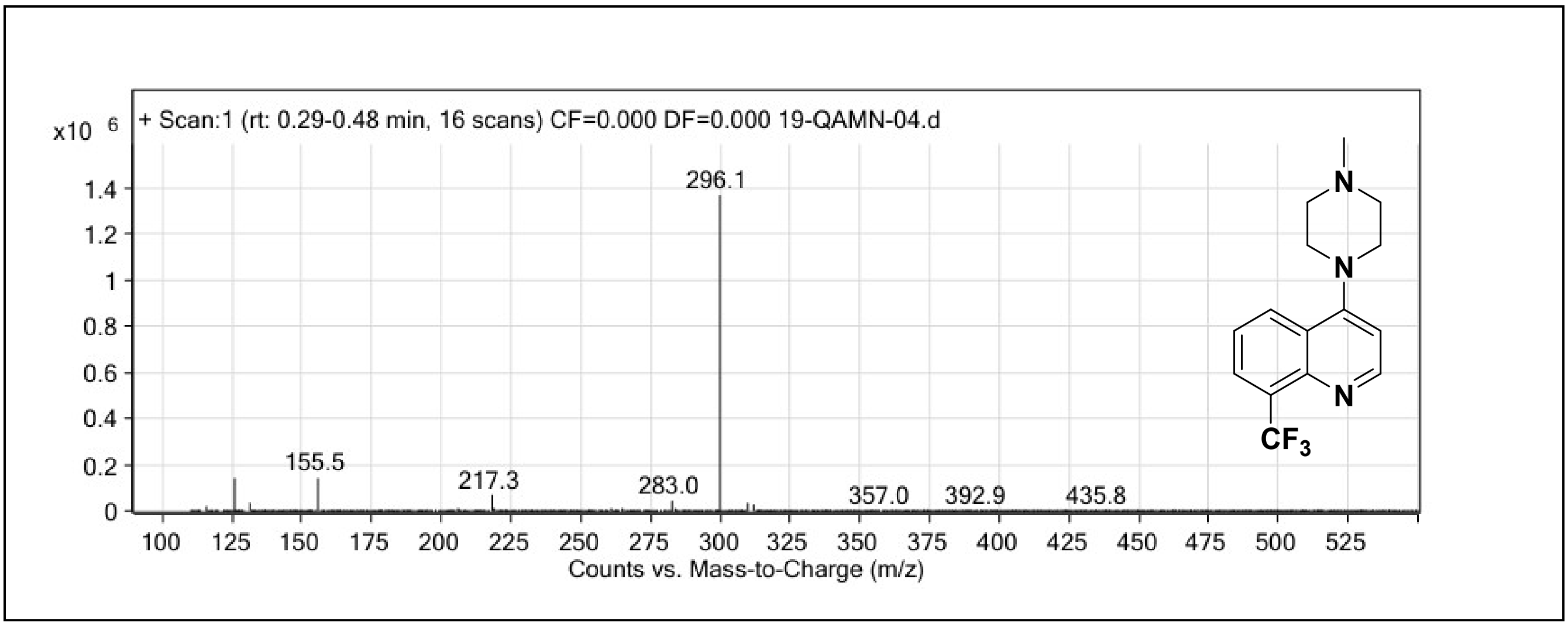

Figure S12. Mass spectrum of 4-(4-methylpiperazin-1-yl)-8-(trifluoromethyl) quinoline (**4d**).

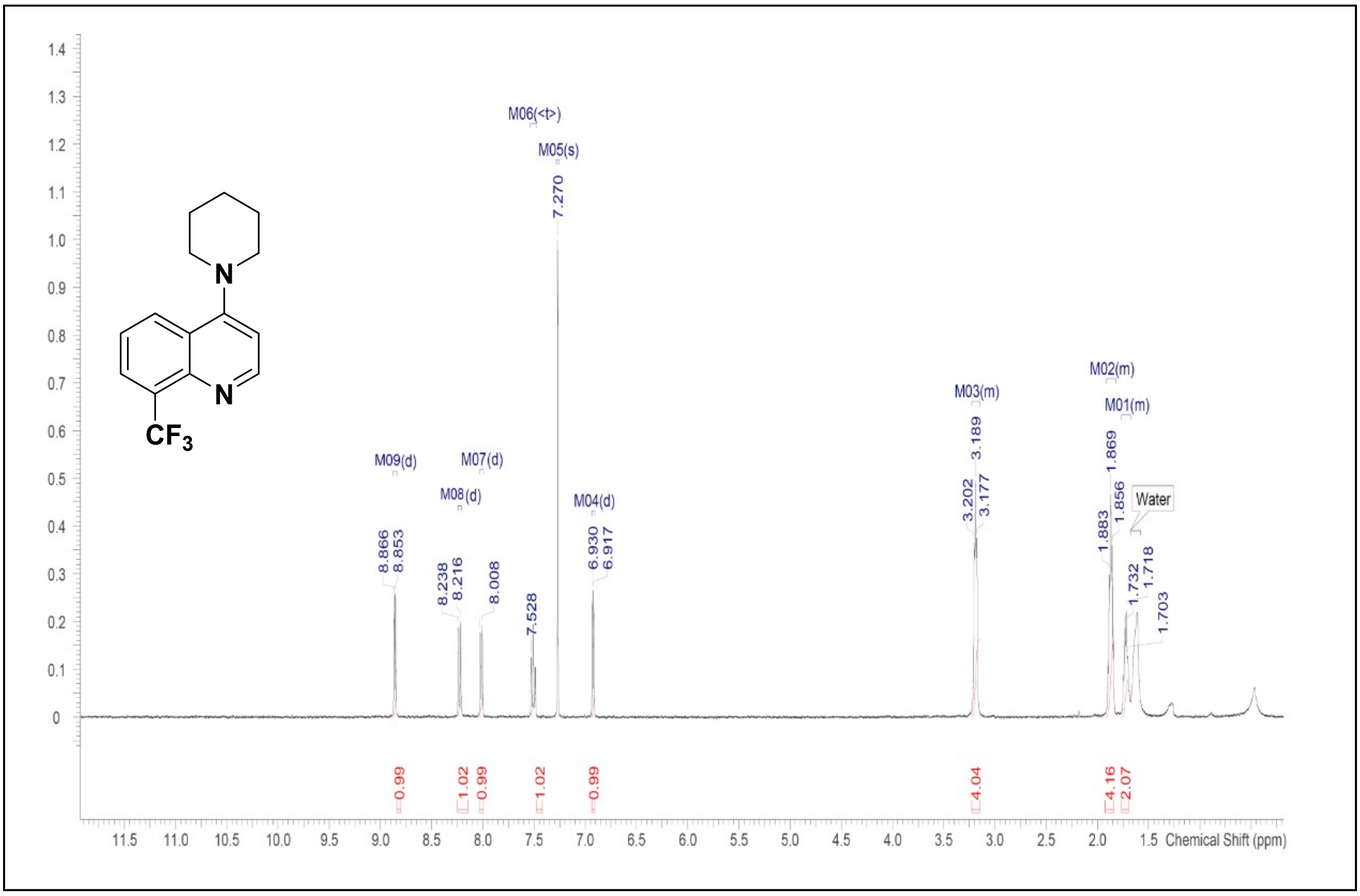

Figure S13. ^{1}H-NMR spectrum of 4-(piperidin-1-yl)-8-(trifluoromethyl) quinoline (**4e**).

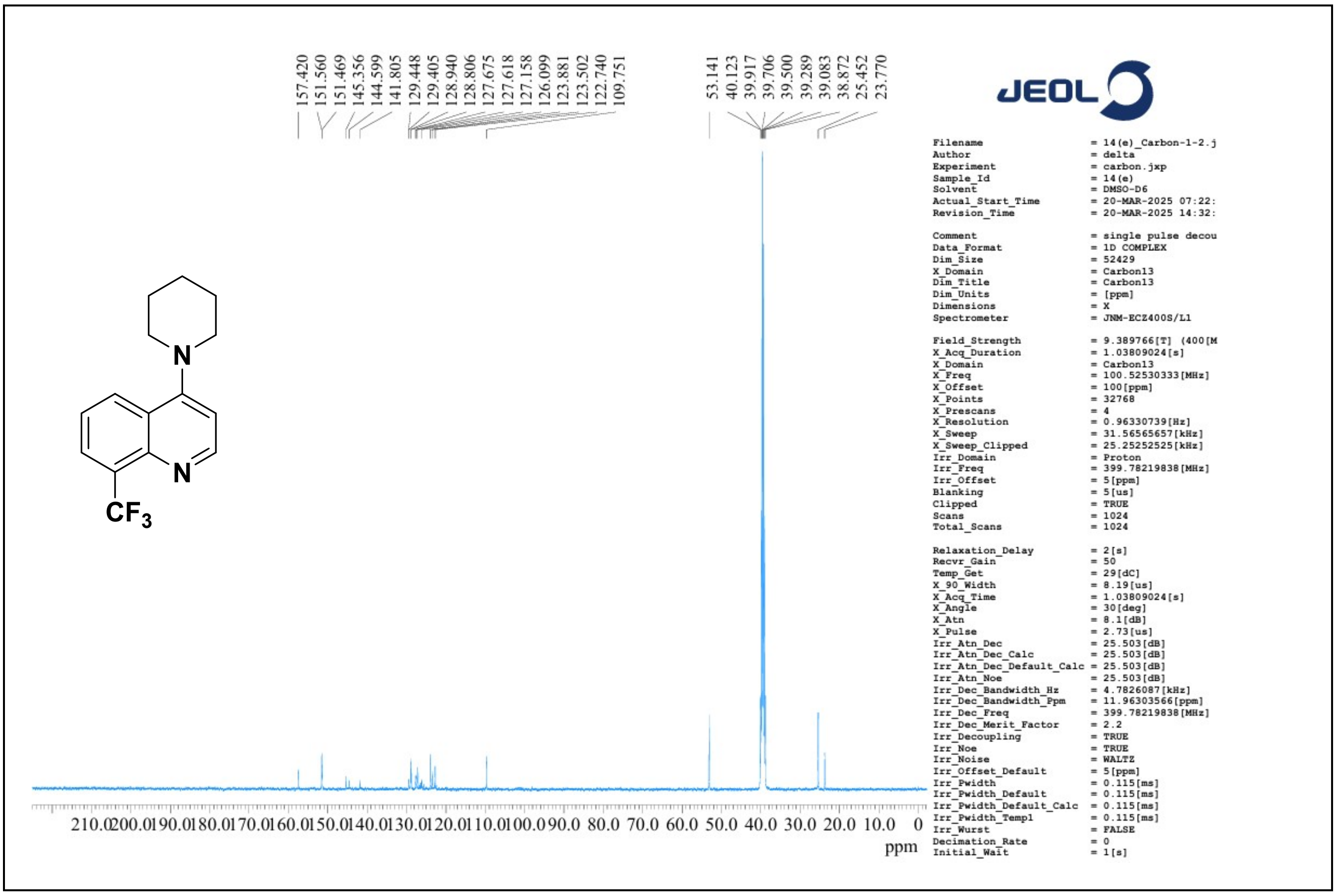

Figure S14. ^{13}C -NMR spectrum of 4-(piperidin-1-yl)-8-(trifluoromethyl) quinoline (**4e**).

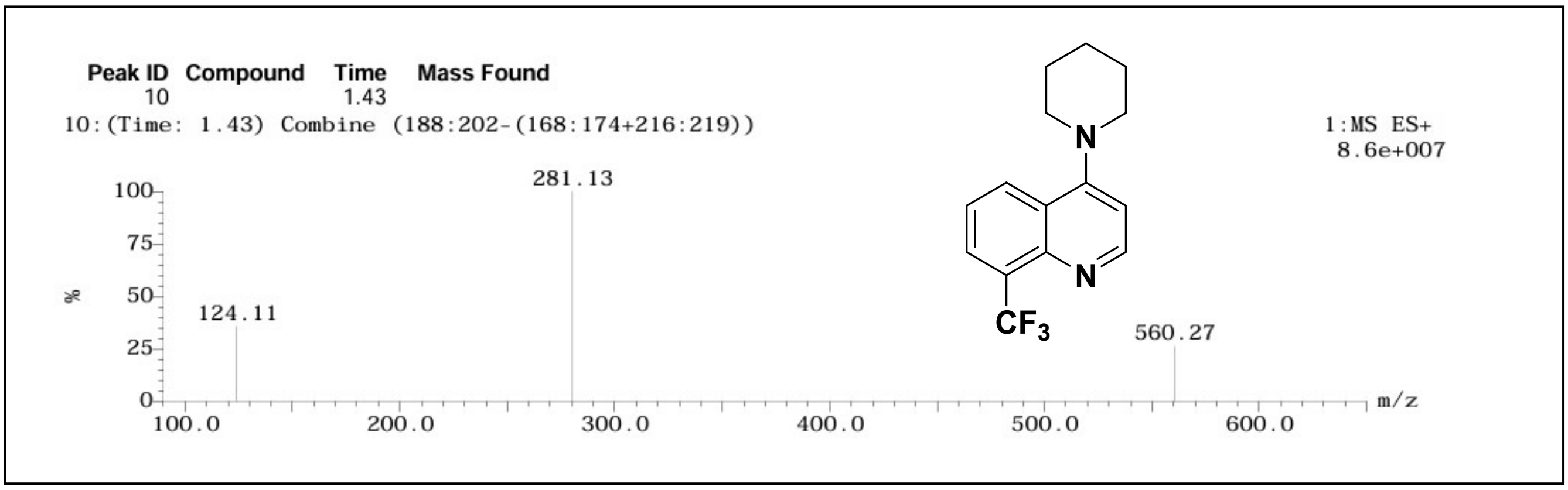

Figure S15. Mass spectrum of 4-(piperidin-1-yl)-8-(trifluoromethyl) quinoline (**4e**).

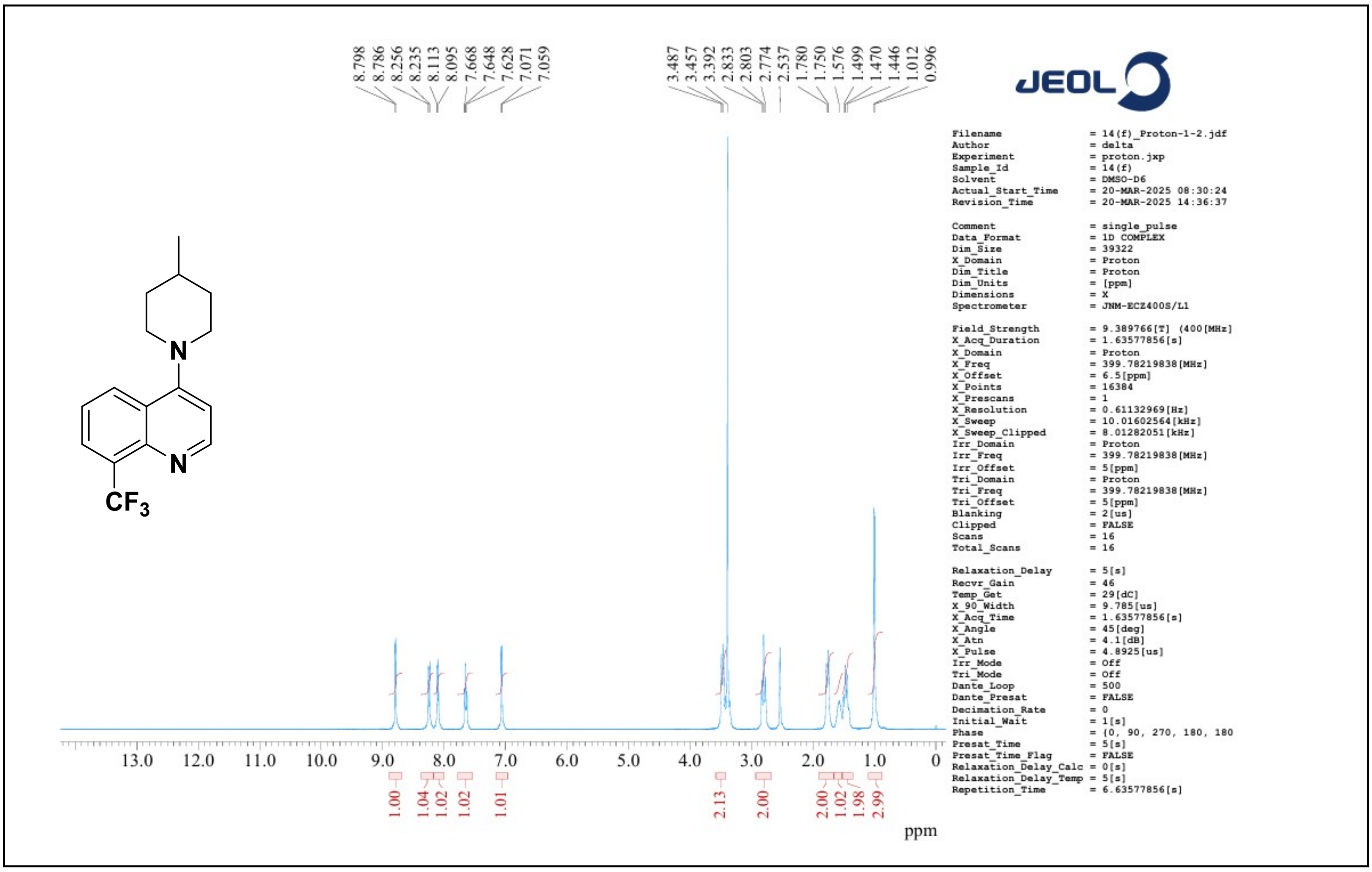

Figure S16. ^{1}H-NMR spectrum of 4-(4-methylpiperidin-1-yl)-8-(trifluoromethyl) quinoline (**4f**).

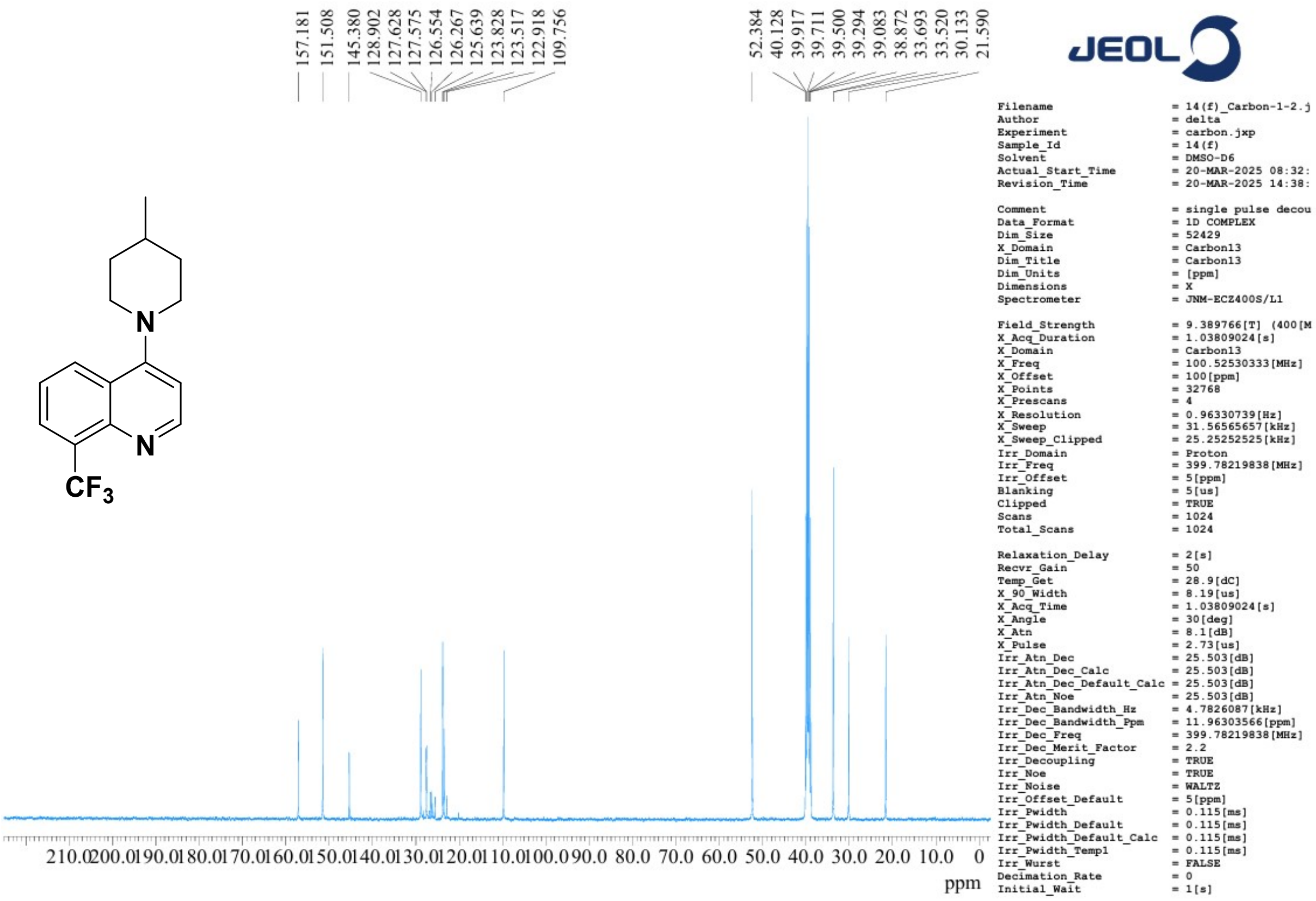

Figure S17. ^{13}C- NMR spectrum of 4-(4-methylpiperidin-1-yl)-8-(trifluoromethyl) quinoline (**4f**).

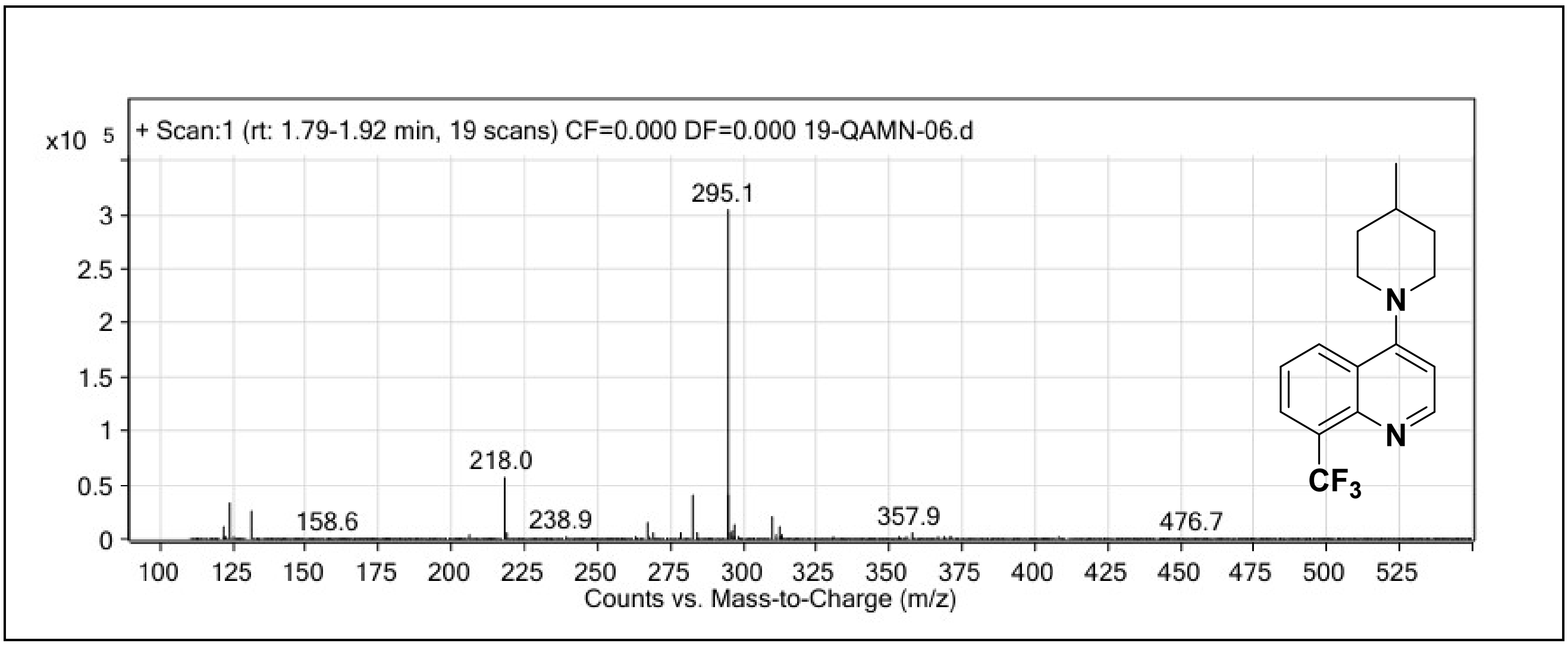

Figure S18. Mass spectrum of 4-(4-methylpiperidin-1-yl)-8-(trifluoromethyl) quinoline (**4f**).

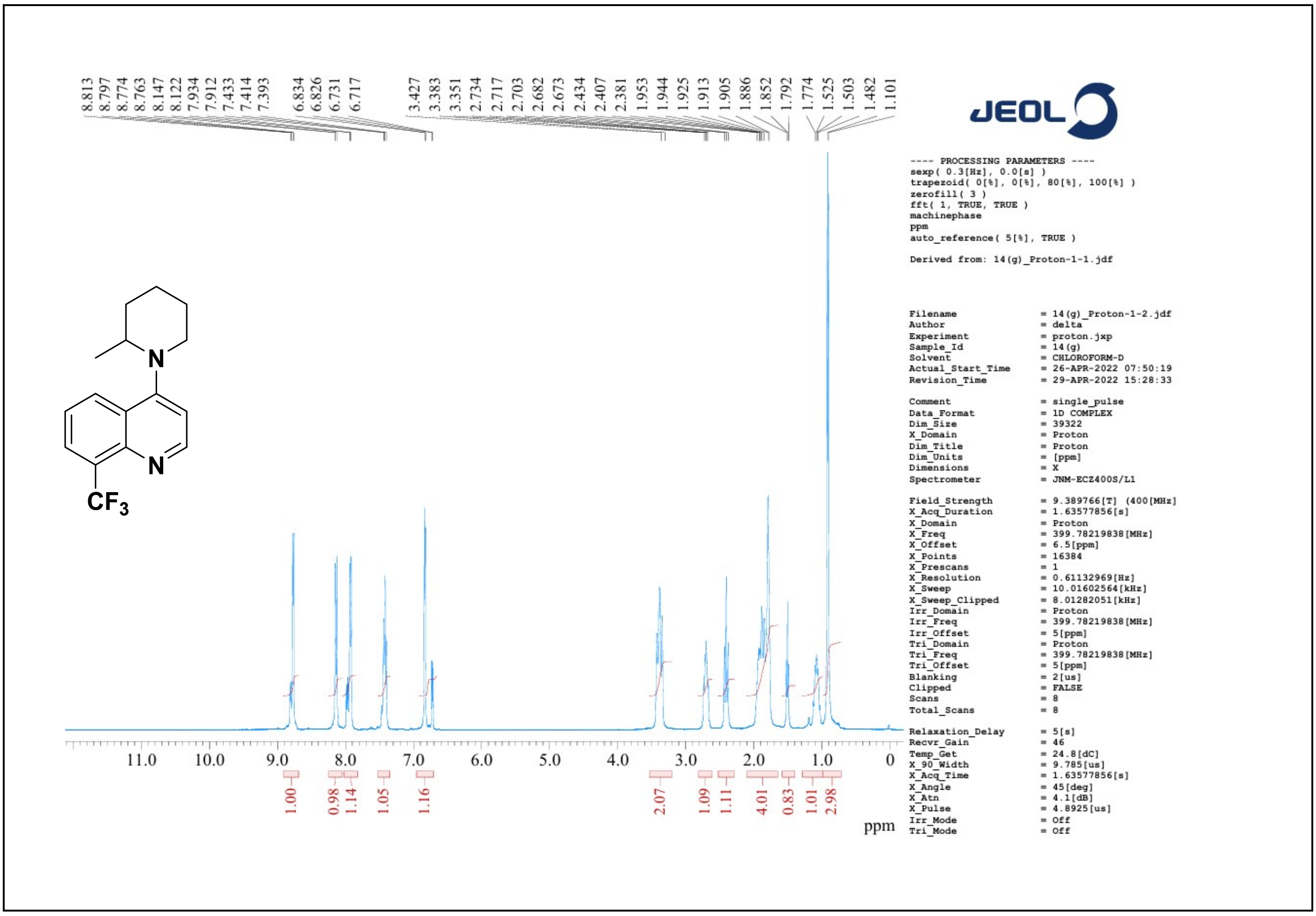

Figure S19. ^{1}H- NMR spectrum of 4-(2-methylpiperidin-1-yl)-8-(trifluoromethyl)quinoline (**4g**).

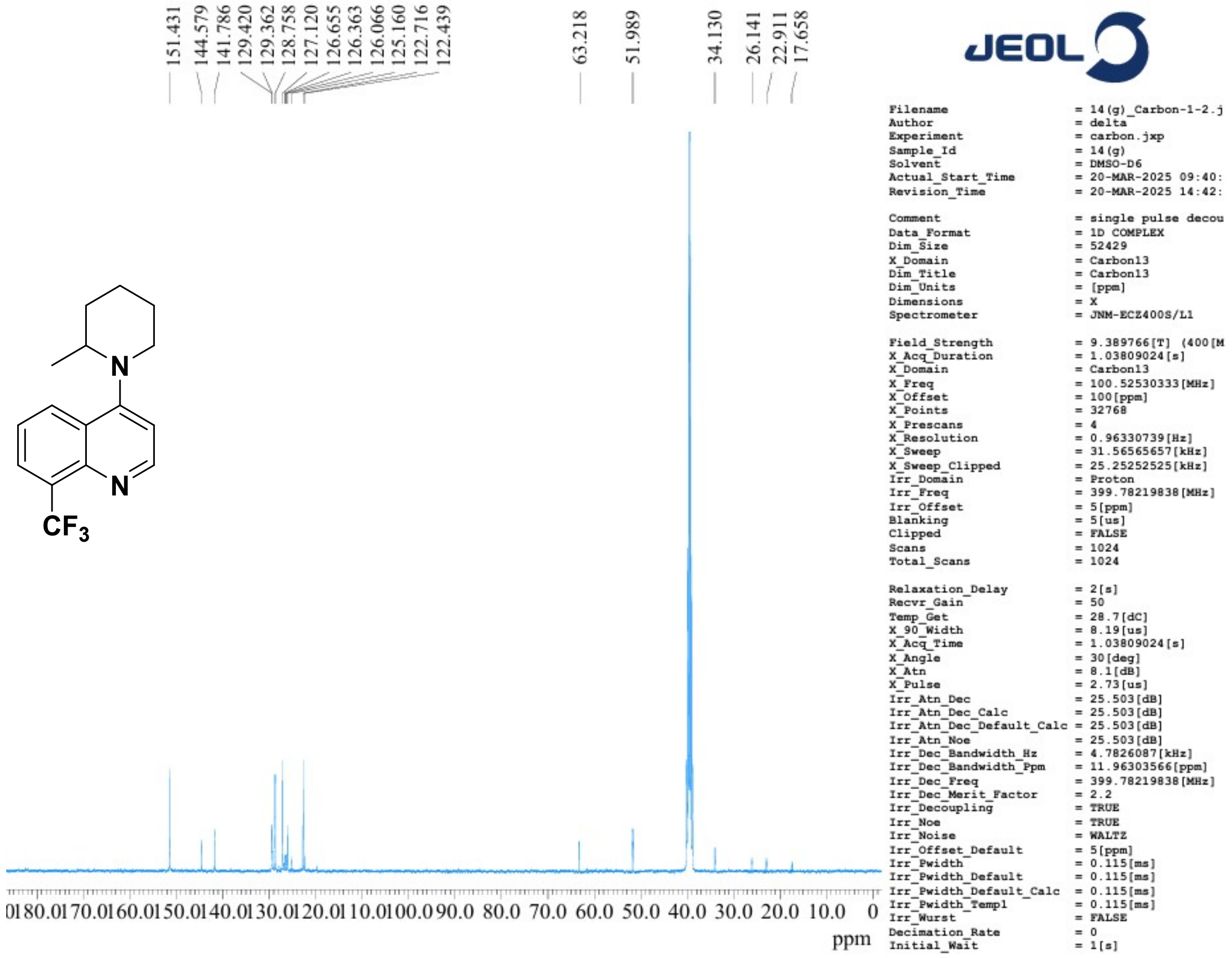

Figure S20. ^{13}C- NMR spectrum of 4-(2-methylpiperidin-1-yl)-8-(trifluoromethyl)quinoline (**4g**).

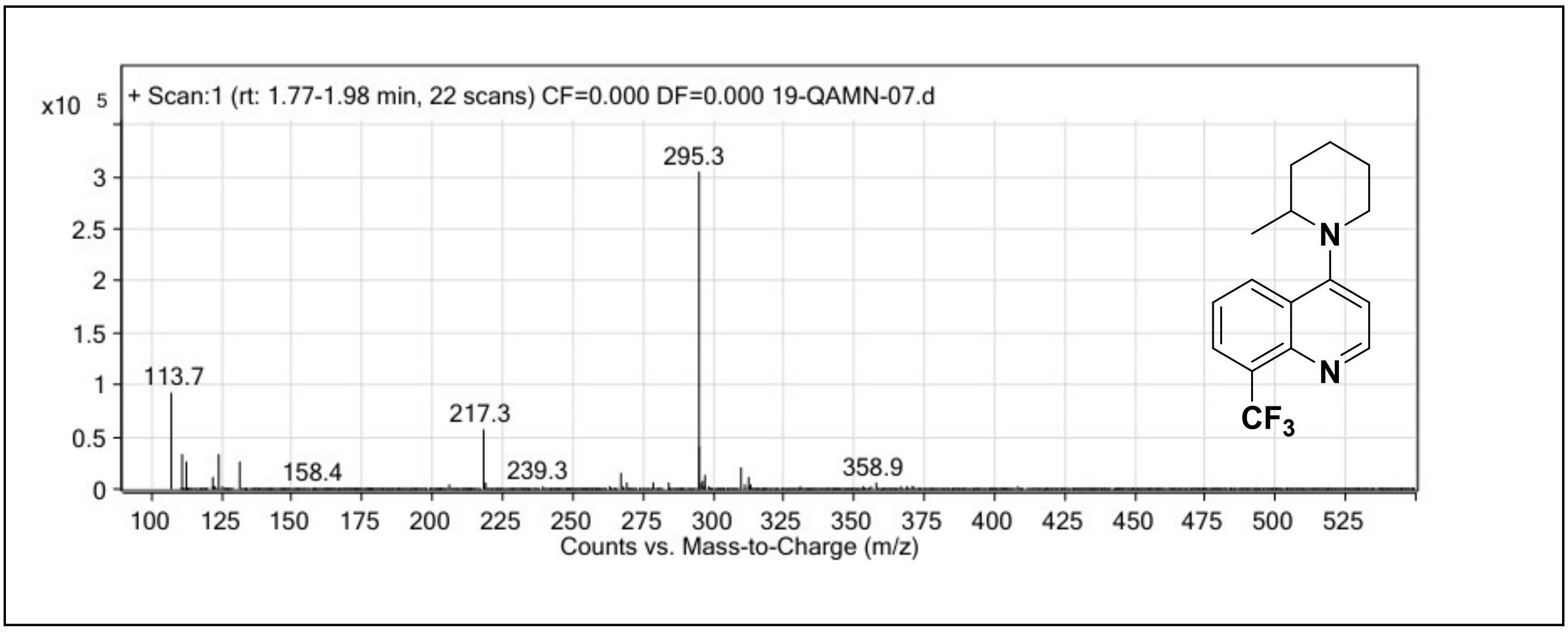

Figure S21. Mass spectrum of 4-(2-methylpiperidin-1-yl)-8-(trifluoromethyl) quinoline (**4g**).

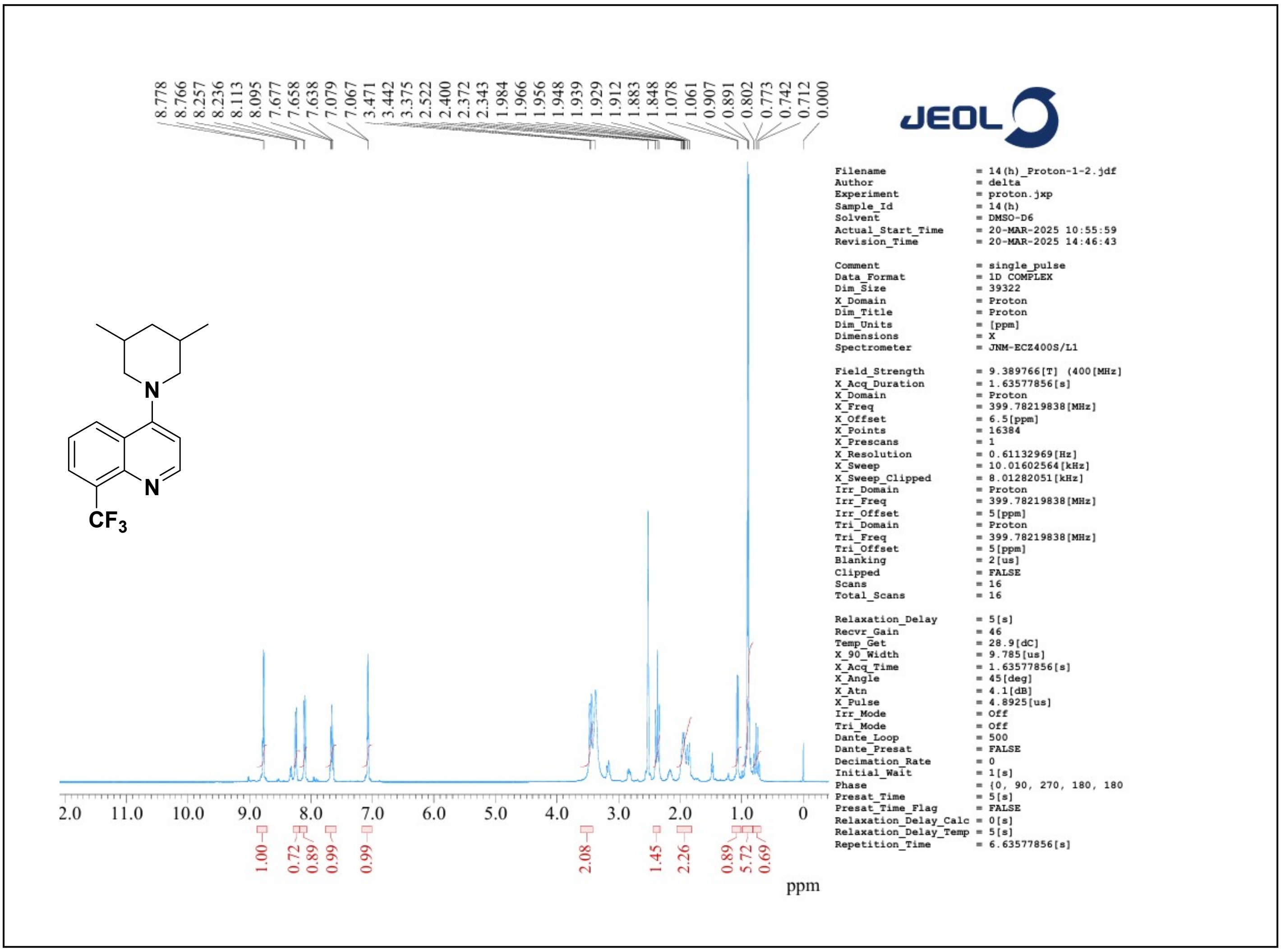

Figure S22. ^{1}H-NMR spectrum of 4-(3,5-dimethylpiperidin-1-yl)-8-(trifluoromethyl) quinoline (**4h**).

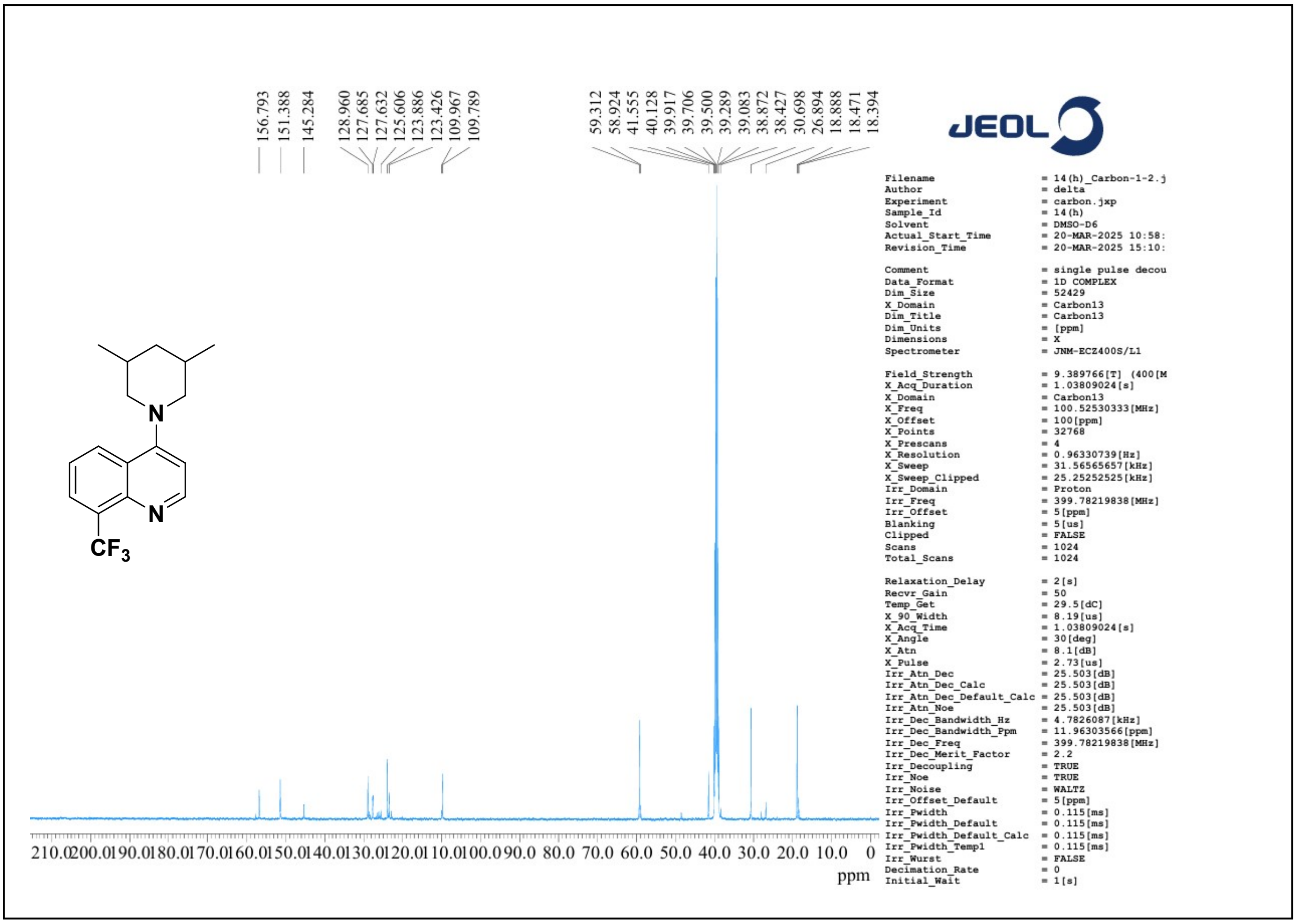

Figure S23. ^{13}C-NMR spectrum of 4-(3,5-dimethylpiperidin-1-yl)-8-(trifluoromethyl)quinoline (**4h**).

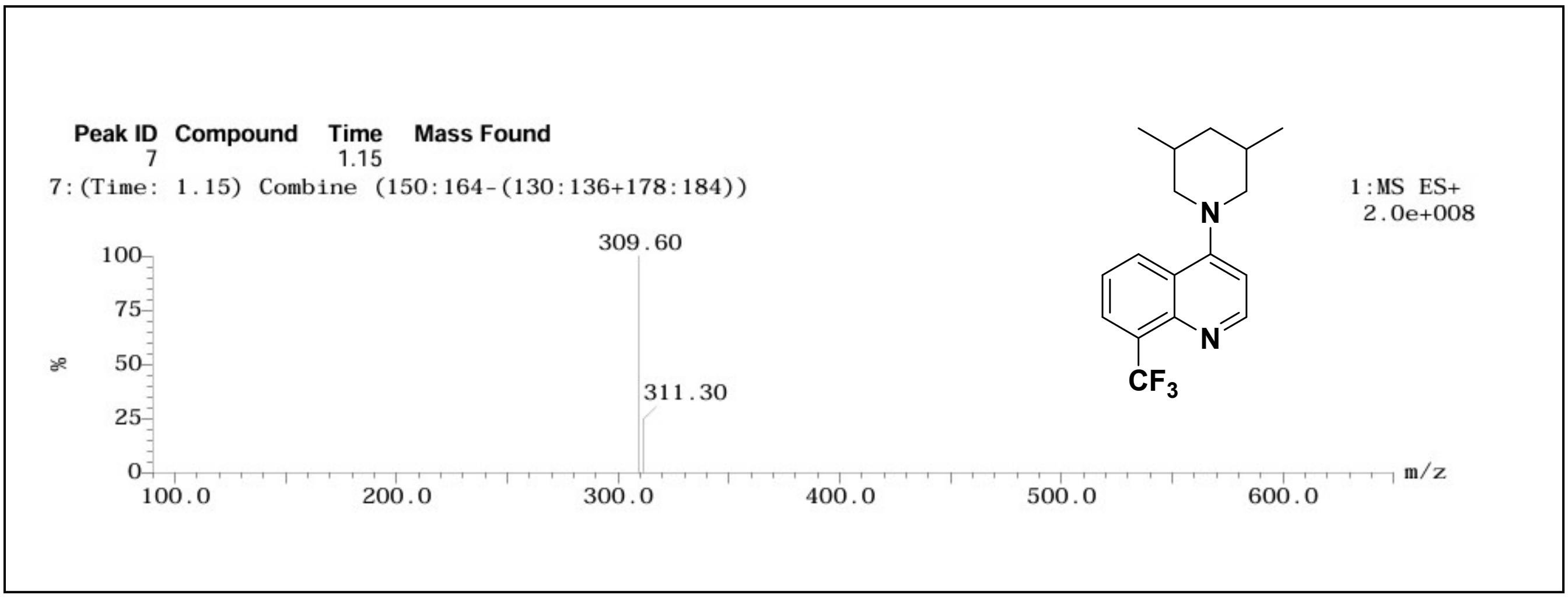

Figure S24. Mass spectrum of 4-(3,5-dimethylpiperidin-1-yl)-8-(trifluoromethyl) quinoline (**4h**).

Table S1. Bond lengths for compound **4a** (Å).

Atom	Length	Atom	Length
F1-C10	1.336(3)	C8-C9	1.372(3)
F2-C10	1.333(2)	C9-C10	1.492(3)
F3-C10	1.341(3)	C11-C12	1.510(3)
O1-C12	1.418(3)	C13-C14	1.504(3)
O1-C13	1.418(3)	C4-H4	0.93
N1-C1	1.366(2)	C5-H5	0.93
N1-C5	1.315(2)	C6-H6	0.93
N2-C3	1.406(2)	C7-H7	0.93
N2-C11	1.462(3)	C8-H8	0.93
N2-C14	1.464(3)	C4-C5	1.392(3)
C1-C2	1.418(3)	C6-C7	1.351(3)
C1-C9	1.422(2)	C7-C8	1.408(3)
C2-C3	1.437(2)	C3-C4	1.366(3)
C2-C6	1.415(2)		

Table S2. Bond angles for compound **4a** (°).

Atom	Angle	Atom	Angle
C12-O1-C13	109.74(2)	N2-C11-C12	110.01(2)
C1-N1-C5	116.05(2)	O1-C12-C11	110.5(2)
C3-N2-C11	116.33(1)	O1-C13-C14	111.33(2)
C3-N2-C14	116.41(2)	N2-C14-C13	109.78(2)
C11-N2-C14	109.46(2)	C3-C4-H4	120
N1-C1-C2	123.37(1)	C5-C4-H4	120
N1-C1-C9	117.98(2)	N1-C5-H5	117
C2-C1-C9	118.62(2)	C4-C5-H5	117
C1-C2-C3	117.63(1)	C2-C6-H6	119
C1-C2-C6	118.87(2)	C7-C6-H6	119
C3-C2-C6	123.40(2)	C6-C7-H7	120
N2-C3-C2	119.54(2)	C8-C7-H7	120
N2-C3-C4	123.19(2)	C7-C8-H8	120
C2-C3-C4	117.21(2)	C9-C8-H8	120
C3-C4-C5	120.10(2)	C1-C9-C8	120.35(2)
N1-C5-C4	125.29(2)	C1-C9-C10	119.63(2)
C2-C6-C7	121.38(2)	C8-C9-C10	120.02(2)
C6-C7-C8	120.18(2)	F1-C10-F2	106.51(2)
C7-C8-C9	120.47(2)	F1-C10-F3	105.71(2)
F2-C10-F3	105.23(2)	F1-C10-C9	113.85(2)
F2-C10-C9	113.46(2)	C14-C13-H13A	109
F3-C10-C9	111.41(2)	C14-C13-H13B	109

Table S3. Torsion angle for the compound **4a** (°).

Atom	Bond angle (°)	Atom	Bond angle(°)	Atom	Bond angle(°)
C12-O1-C13-C14	60.0(2)	N1-C1-C2-C3	2.7(2)	N2-C3-C4-C5	-178.16(17)
C13-O1-C12-C11	-60.0(2)	N1-C1-C9-C8	176.28(17)	C3-C4-C5-N1	0.7(3)
C5-N1-C1-C9	-175.19(16)	C9-C1-C2-C3	-179.69(15)	C2-C6-C7-C8	0.6(3)
C1-N1-C5-C4	-4.3(3)	N1-C1-C2-C6	-173.65(16)	C6-C7-C8-C9	2.1(3)
C5-N1-C1-C2	2.5(2)	C2-C1-C9-C10	179.23(16)	C7-C8-C9-C1	-1.6(3)
C14-N2-C3-C2	163.45(16)	C2-C1-C9-C8	-1.5(3)	C7-C8-C9-C10	177.73(18)
C3-N2-C11-C12	168.83(18)	N1-C1-C9-C10	-3.0(2)	C1-C9-C10-F2	-59.0(2)
C14-N2-C11-C12	-56.7(2)	C6-C2-C3-N2	-7.2(3)	C8-C9-C10-F2	121.7(2)
C14-N2-C3-C4	-13.6(2)	C3-C2-C6-C7	-179.70(17)	C1-C9-C10-F3	-177.51(16)
C3-N2-C14-C13	-169.51(16)	C1-C2-C6-C7	-3.6(3)	C1-C9-C10-F1	63.1(2)
C11-N2-C14-C13	56.0(2)	C6-C2-C3-C4	170.02(17)	C8-C9-C10-F3	3.2(3)
C11-N2-C3-C4	117.7(2)	C1-C2-C3-C4	-6.1(2)	C8-C9-C10-F1	-116.2(2)
C11-N2-C3-C2	-65.2(2)	C1-C2-C3-N2	176.61(15)	N2-C11-C12-O1	59.0(2)
C9-C1-C2-C6	4.0(2)	C2-C3-C4-C5	4.7(3)	O1-C13-C14-N2	-58.2(2)

Table S4. Tabular presentation of in silico ADME parameters for all compounds (**4a-h**) under study

Properties		Units	Compounds Code							
			**4a**	**4b**	**4c**	**4d**	**4e**	**4f**	**4g**	**4h**
**Physiochemical parameters**	Formula		$C_{14}H_{13}F_3N_20$	$C_{20}H_{18}F_3N_3$	$C_{16}H_{18}F_3N_3$	$C_{15}H_{16}FN_3$	$C_{15}H_{15}F_3N_2$	$C_{16}H_{17}F_3N_2$	$C_{16}H_{17}F_3N_2$	$C_{17}H_{19}F_3N_2$
	Molecular weight	g/mol	282.26	357.359	309.335	295.308	280.293	294.32	294.32	308.47
	Molar refractivity		72.5	106.21	87.84	83.04	76.22	81.03	81	81.03
	TPSA	Å^2	37.38	40.51	31.39	31.39	28.15	28.15	16.13	28.15
	LogP		3.092	4.5802	3.3955	3.0054	4.239	4.4899	4.6324	4.739
**Lipophilicity**	iLOGP		-1.04	-9.77	-0.34	-0.56	-0.59	-0.38	3.01	-0.33
	SILICOS-IT		3.35	4.04	3.32	2.95	3.97	4.05	4.07	4.05
**Water Solubility**	log mol/L		-4.299	-5.712	-4.038	-3.791	-5.117	-5.364	-5.357	-5.579
**Pharmacokinetics**	Intestinal absorption (human)	%	96.577	95.257	93.393	93.812	94.762	94.3	93.975	94.03
	VDss (human)	L/kg	0.075	0.304	1.115	1.02	0.371	0.556	0.573	0.606
	Fraction unbound (human)	%	0.269	0.046	0.323	0.343	0.224	0.178	0.191	0.138
	P-glycoprotein substrate	Yes/No	No	No	Yes	Yes	Yes	Yes	Yes	Yes
	BBB permeant	Yes/No	Yes	Yes	Yes	Yes	Yes	Yes	Yes	Yes
	Log K_p (skin permeability)	cm/s	-2.453	-2.465	-2.619	-2.59	-2.069	-2.325	-2.334	-2.33
	CYP1A2	Yes/No	Yes	Yes	Yes	No	Yes	Yes	Yes	Yes
	CYP2D6	Yes/No	Yes	No	Yes	Yes	Yes	Yes	Yes	Yes
	P-glycoprotein I inhibitor	Yes/No	No	Yes	NO	No	No	No	No	No
	P-glycoprotein II inhibitor	Yes/No	No	Yes	No	No	No	No	No	No
**Drug-likeness Rules**	Lipinski (Pfizer)	Yes/No	Yes	Yes	Yes	Yes	Yes	Yes	Yes	Yes
	Ghose (Amgen)	Yes/No	Yes	Yes	Yes	Yes	Yes	Yes	Yes	Yes
	Egan (Pharmacia)	Yes/No	Yes	Yes	Yes	Yes	Yes	Yes	Yes	Yes

	Muege (Bayer)	Yes/No	Yes	Yes	Yes	Yes	Yes	Yes	Yes	Yes
	Bioavailibility Score		0.55	0.55	0.55	0.55	0.55	0.55	0.55	0.55
**Medicinal Chemistry**	PAINS	alert	0	0	0	0	0	0	0	0
	Brenk	alert	0	0	0	0	0	0	0	0
	Synthetic accessibility	1: very easy; 10: very difficult	2.65	3.38	2.86	2.76	2.42	2.97	2.45	2.98
**Excretion**	Total Clearance	ml/min/kg	0.179	0.305	0.517	0.47	0.17	0.141	0.18	0.118
	Renal OCT2 substrate	Yes/No	No	Yes	No	No	No	No	No	No

Table S5: Grid box size.

	1ZP4	6ARY	2CH2	4JBV	5V13
X	9.423	-84.874	-17.517	13.345	235.247
Y	4.593	51.825	39.759	15.331	-4.963
Z	13.362	-10.890	-41.610	63.030	362.550

Table S6. Molecular docking results for protein PDB ID: 1ZP4 against the malaria vector.

Compound code	Binding Score (kcal/mol)	Binding Interactions	Amino acids
**5**	-8.3	Halogen (Fluorine), Pi-Sigma Pi-Pi Stacked, Alkyl, Pi-Alkyl	GLN219, LEU277, PHE223, LEU212,
**6**	-9.0	Carbon Hydrogen Bond, Halogen (Fluorine), Pi-Pi Stacked, Alkyl, Pi-Alkyl	ARG33,PRO31, PHE30, PHE223, TYR275,
**7**	-8.3	Carbon Hydrogen Bond; Halogen (Fluorine), Pi-Sigma, Pi-Pi Stacked Alkyl	ALA220, GLN219, LEU277, PHE223, LEU212
**8**	-8.3	Halogen (Fluorine), Pi-Pi Stacked Alkyl, Pi-Alkyl	GLN219, LEU277, PHE223, LEU212, LEU277
**9**	-8.7	Conventional Hydrogen Bond;Halogen (Fluorine), Carbon Hydrogen Bond, Alkyl, Pi-Alkyl	ARG197, ARG193, ALA162,
**10**	-8.8	Halogen (Fluorine), Pi-Pi Stacked Alkyl, Pi-Alkyl	GLN219, LEU277, PHE223, LEU212, LEU277, PHE30
**11**	-9.1	Conventional Hydrogen Bond; Halogen (Fluorine), Carbon Hydrogen Bond, Pi-Alkyl	GLN28, LEU277, PHE223, LEU212, TYR275
**12**	-8.7	Pi-Pi Sigma, Pi-Pi Stacked, Alkyl Pi-Alkyl	LEU277, PHE223, LEU212, PHE184, TYR275
**13**	-8.3	Conventional Hydrogen Bond; Halogen (Fluorine), Pi-Pi Sigma, Pi-Pi Stacked, Alkyl, Pi-Alkyl	ALA220, GLN219, LEU277, PHE223, LEU212,

Table S7. Molecular docking results for protein PDB ID: 2CH2 against the malaria vector.

Compound code	Binding Score (kcal/mol)	Binding Interactions	Amino acids
**5**	-7.6	Conventional Hydrogen Bond; Halogen (Fluorine), Carbon Hydrogen Bond, Halogen (Fluorine), Pi-Cation, Pi-Donor Hydrogen Bond, Pi-Pi Stacked, Alkyl	LYS205,ASN44,PRO24.GLY25TYR256,LEU347
**6**	-9.3	Pi-Cation, Pi-Pi Stacked, Alkyl, Pi-Alkyl	ARG356, PHE45, TYR256,LEU347
**7**	-8.0	Conventional Hydrogen Bond; Halogen (Fluorine), Carbon Hydrogen Bond, Halogen (Fluorine), Pi-Cation, Pi-Donor Hydrogen Bond, Pi-Sigma, Pi-Pi Stacked, Alkyl	LYS205, ASN44,SER43,PRO24, GLY25,ARG356, PHE45, LEU347
**8**	-7.8	Conventional Hydrogen bond; Halogen (Fluorine), Carbon Hydrogen Bond, Halogen (Fluorine), Pi-Cation, Pi-Donor Hydrogen Bond, Pi-Pi Stacked, Alkyl	LYS205,ASN44,SER43, PRO24 GLY25, RG356, TYR256, EU347, PHE45
**9**	-8.0	Conventional Hydrogen bond; Halogen (Fluorine), Carbon Hydrogen Bond, Halogen (Fluorine), Pi-Cation, Pi-Donor Hydrogen Bond, Pi-Pi Stacked, Alkyl	LYS205, ASN44, PRO24, GLY25, ARG356, YR256, LEU347
**10**	-8.0	Carbon Hydrogen Bond, Halogen (Fluorine), Pi-Pi Stacked, Alkyl, Pi-Sigma, Pi-Alkyl	GLN344, YS254, TYR256, PHE45, LEU347, PHE45
**11**	-8.1	Conventional Hydrogen bond; Halogen (Fluorine), Pi-Cation, Pi-Donor Hydrogen Bond, Pi-Pi T-shaped, Pi-Pi Stacked, Alkyl	GLN333, HIS46, SER332, LU342, TRP329, ET336, PHE45, HIS46
**12**	-8.8	Conventional Hydrogen bond; Halogen (Fluorine), Pi-Cation, Pi-Donor Hydrogen Bond, Pi-Pi Stacked, Alkyl, Pi-Alkyl	LYS205, ASN44,PRO24,GLY25, ARG356, ASN44, TYR256, LEU347, PHE45,
**13**	-8.8	Conventional Hydrogen bond; Halogen (Fluorine), Pi-Cation, Pi-Donor Hydrogen Bond, Pi-Pi Stacked, Alkyl, Pi-Alkyl	LYS205, ASN44, PRO24, GLY25, ARG356, ASN44, TYR256, LEU347, PHE45

Table S8. Molecular docking results for protein PDB ID: 4JBV against the malaria vector.

Compound code	Binding Score (kcal/mol)	Binding Interactions	Amino acids
**5**	**-7.7**	Carbon Hydrogen Bond, Pi-Sigma, Alkyl, Pi-Alkyl	LEU57, GLU135, LYS80, VAL65, MET112, ILE194, ALA78, LEU181
**6**	**-9.5**	Carbon Hydrogen Bond, Halogen (Fluorine), Pi-Sulphur Pi-Sigma, Alkyl, Pi-Alkyl	ASP195, TYR131, MET112, VAL65, LEU57, ILE194, ALA78 , LYS80, LEU181, LEU126
**7**	**-7.9**	Carbon Hydrogen Bond, Pi-Sigma, Alkyl, Pi-Alkyl	LEU57, GLU135, LYS80, VAL65, MET112, ILE194, ALA78, LEU181,
**8**	**-7.8**	Carbon Hydrogen Bond, Pi-Sigma, Alkyl, Pi-Alkyl	LEU57, GLU135, LYS80, VAL65, MET112, ILE194, ALA78, LEU181,
**9**	**-7.9**	Carbon Hydrogen Bond, Pi-Sigma, Alkyl, Pi-Alkyl	GLU135, LYS80, VAL65, MET112, ILE194, ALA78, LEU181,
**10**	**-8.5**	Carbon Hydrogen Bond, Halogen (Fluorine), Pi-Sulphur Pi-Sigma, Alkyl, Pi-Alkyl	GLU129, TYR131, VAL65, MET112, LEU57, LEU181, LYS80, ILE194, ALA78,
**11**	**-8.6**	Carbon Hydrogen Bond, Pi-Sigma, Alkyl, Pi-Alkyl	LYS80, VAL65, LEU181, MET112, ILE194, LEU57, ALA78,
**12**	**-8.4**	Carbon Hydrogen Bond, Pi-Sigma, Alkyl, Pi-Alkyl	LYS80, VAL65, MET112, ILE194, LEU181, ALA78
**13**	**-8.2**	Carbon Hydrogen Bond, Pi-Sigma, Alkyl, Pi-Alkyl	GLU135, LYS80, VAL65, ILE194, LEU181, LEU57, ALA78,

Table S9. Molecular docking results for protein PDB ID: 5V13 against the malaria vector.

Compound code	Binding Score (kcal/mol)	Binding Interactions	Amino acids
**5**	**-8.4**	Conventional Hydrogen bond; Halogen (Fluorine), Alkyl	LYS175,GLN164, TYR155, ALA156
**6**	**-8.4**	Conventional Hydrogen bond; Halogen (Fluorine), Pi-Alkyl, Pi-Pi T-Shaped	GLN28, GLU27, PHE267, EU158, PHE169
**7**	**-8.4**	Conventional Hydrogen bond; Halogen (Fluorine), Alkyl	LYS175, SN160, GLN164, YR155, ALA156
**8**	**-7.5**	Conventional Hydrogen bond; Halogen (Fluorine), P-Sigma, Alkyl	GLN28, GLU27, CYS195, RG198, ARG201, GLU205, CYS195,
**9**	**-8.5**	Conventional Hydrogen bond; Halogen (Fluorine), P-Cation, Pi-Pi T-Shaped, Amide-Pi Stacked, Alkyl, Pi-Alkyl	HIS96, LYS157, ASN286, ASN153, TYR148, ARG149,
**10**	**-8.2**	Pi-Alkyl, Pi-Pi T-Shaped, Pi-Sigma, Alkyl, Pi-Alkyl	TRP53, VAL51, TYR33, ALA281, PRO55, VA65, LEU37,
**11**	**-9.6**	Conventional Hydrogen Bond, hydrogen bond, Halogen (Fluorine), Alkyl	LYS175, ASN160, ALA156,
**12**	**-8.5**	Carbon Hydrogen bond, Pi-Sigma, Alkyl, Pi-Alkyl	AG198, ARG201
**13**	**-8.6**	Conventional Hydrogen Bond; Halogen (Fluorine), Pi-Cation, Amide-Pi Stacked, Alkyl	HIS96, LYS157,ASN153, ASN286, TYR148, ARG149

Table S10. Molecular docking PDB ID: 6ARY against the malaria vector.

Compound code	Binding Score (kcal/mol)	Binding Interactions	Amino acids
**5**	**-8.3**	Conventional Hydrogen Bond; Halogen (Fluorine), Pi-Pi Stacked, Pi-Alkyl	TYR282, PHE490, TRP441, TYR489
**6**	**-10.0**	Conventional Hydrogen Bond; Halogen (Fluorine), Pi-Pi Stacked, Pi-Anion, Pi-Sigma, Pi-Pi T-shaped	ILE231, TRP245, TYR282, TYR489, ASP233, VAL232
**7**	**-7.9**	Conventional Hydrogen Bond; Halogen (Fluorine), Pi-Pi Stacked, Pi-Alkyl, Amide-Pi Stacked	TYR493, PHE490, TYR489, TYR494, PHE490,
**8**	**-8.0**	Conventional Hydrogen Bond; Halogen (Fluorine), Pi-Pi Stacked, Pi-Alkyl, Amide-Pi Stacked	TYR282, GLY442, PHE490, TRP441, TYR494, TYR489
**9**	**-8.6**	Conventional Hydrogen Bond; Halogen (Fluorine), Pi-Pi Stacked, Pi-Alkyl, Amide-Pi Stacked	TYR282, PHE490, TRP441, TYR493, TYR489
**10**	**-8.1**	Conventional Hydrogen Bond; Halogen (Fluorine), Pi-Pi Stacked, Pi-Anion, Pi-Sigma, Pi-Pi T-shaped, Amide-Pi Stacked	SER283. TRP245, ASP233, TYR282, VAL232,
**11**	**-9.0**	Conventional Hydrogen Bond; Halogen (Fluorine), Pi-Pi T-Shaped, Pi-Alkyl, Amide-Pi Stacked	TYR291, SER360, GLY278,GLU359, TYR489, TRP245
**12**	**-8.4**	Conventional Hydrogen Bond; Halogen (Fluorine), Pi-Pi Stacked, Pi-Anion, Pi-Alkyl, Alkyl, Pi-Pi T-shaped, Amide-Pi Stacked	TYR489, ASP233, TRP245, TYR282, VAL232, ILE231, TRP441
**13**	**-8.4**	Conventional Hydrogen Bond; Halogen (Fluorine), Pi-Pi Stacked, Pi-Alkyl, Alkyl, Amide-Pi Stacked	TYR282, PHE490, TRP441, TYR493, TYR494, LEU444, TYR489

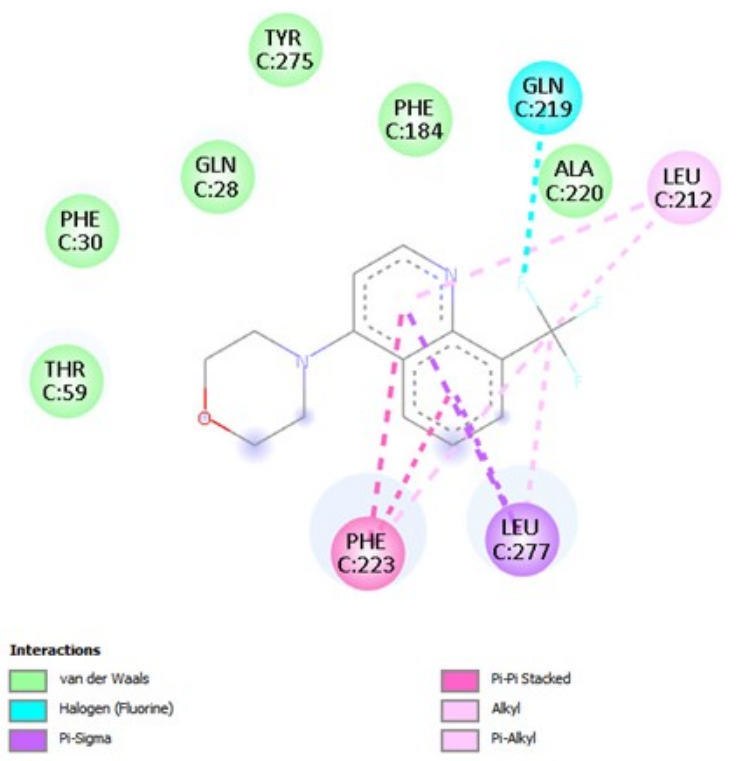

(**a**) 1ZP4-5 complex

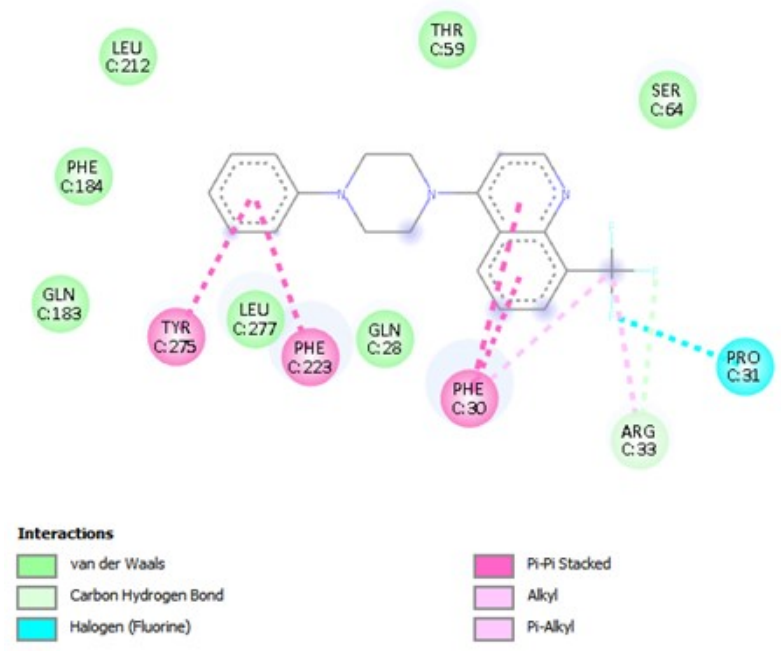

(**b**) 1ZP4-6 complex

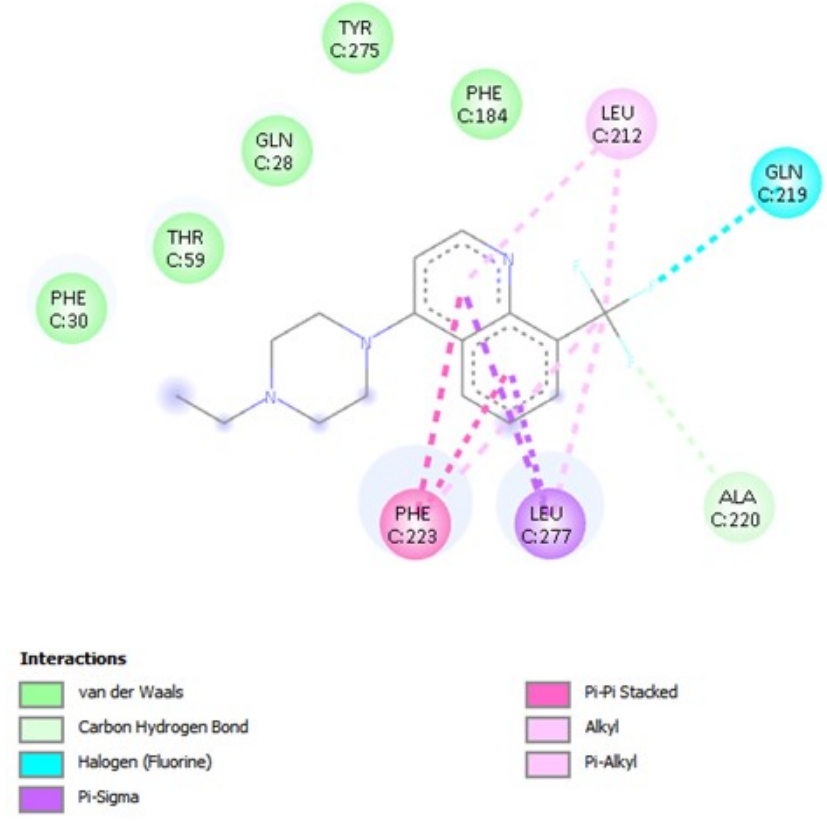

(**c**) 1ZP4-7 complex

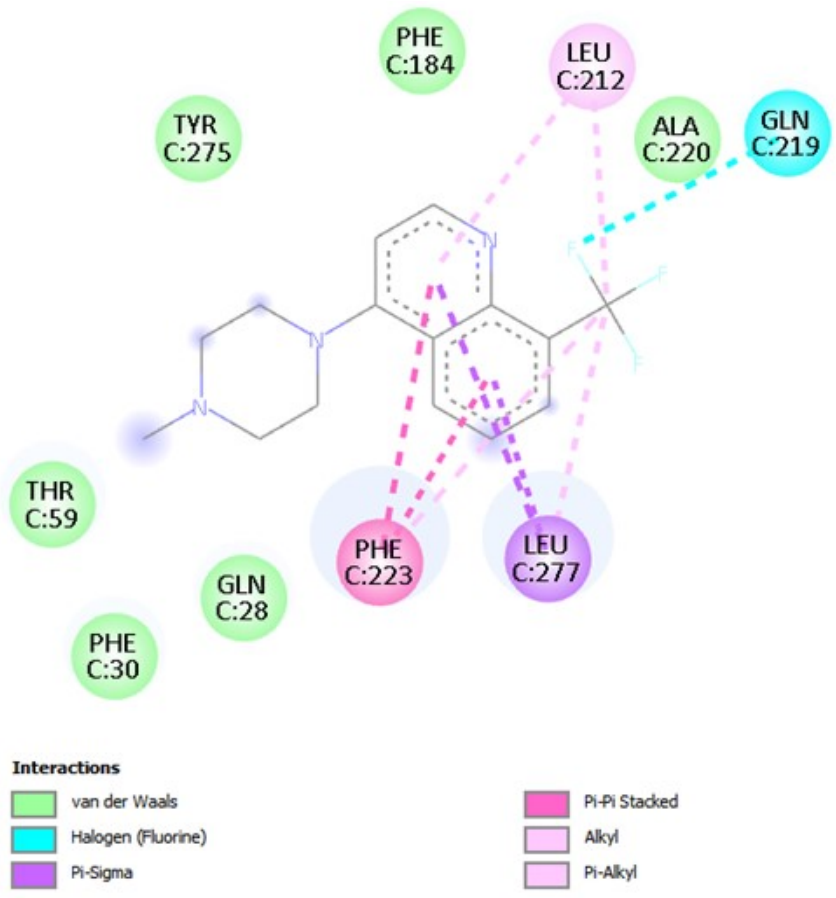

(**d**) 1ZP4-8 complex

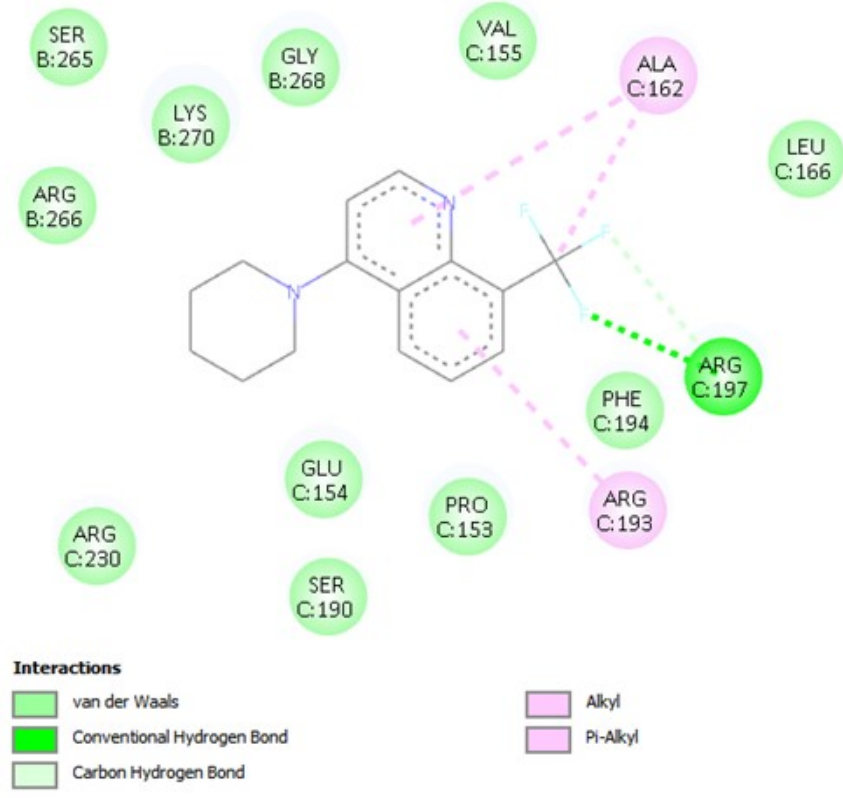

(**e**) 1ZP4-9 complex

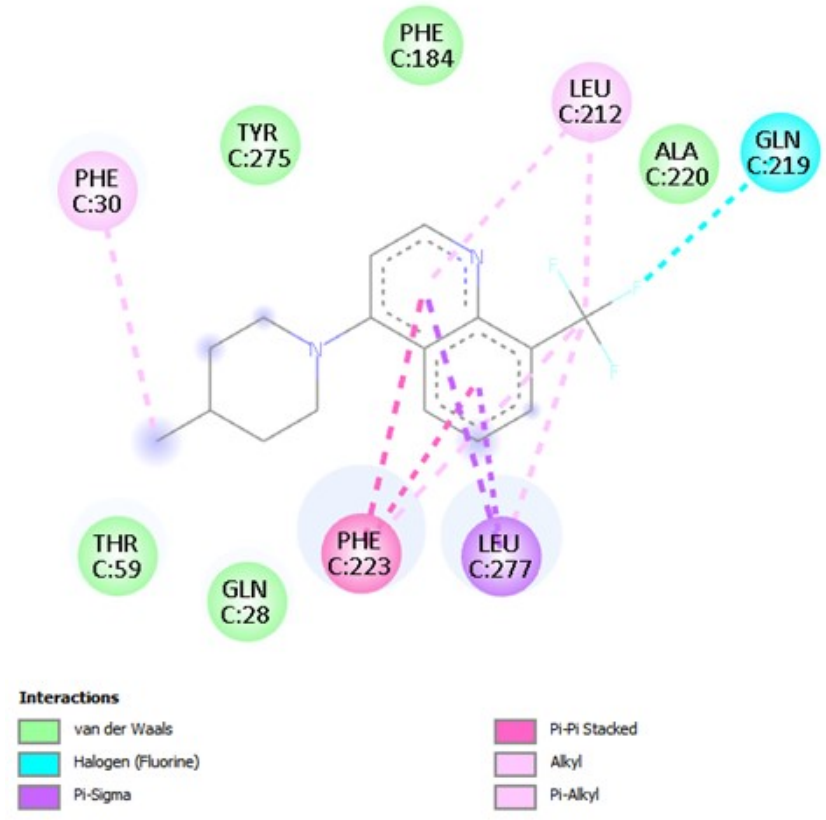

(**f**) 1ZP4-10 complex

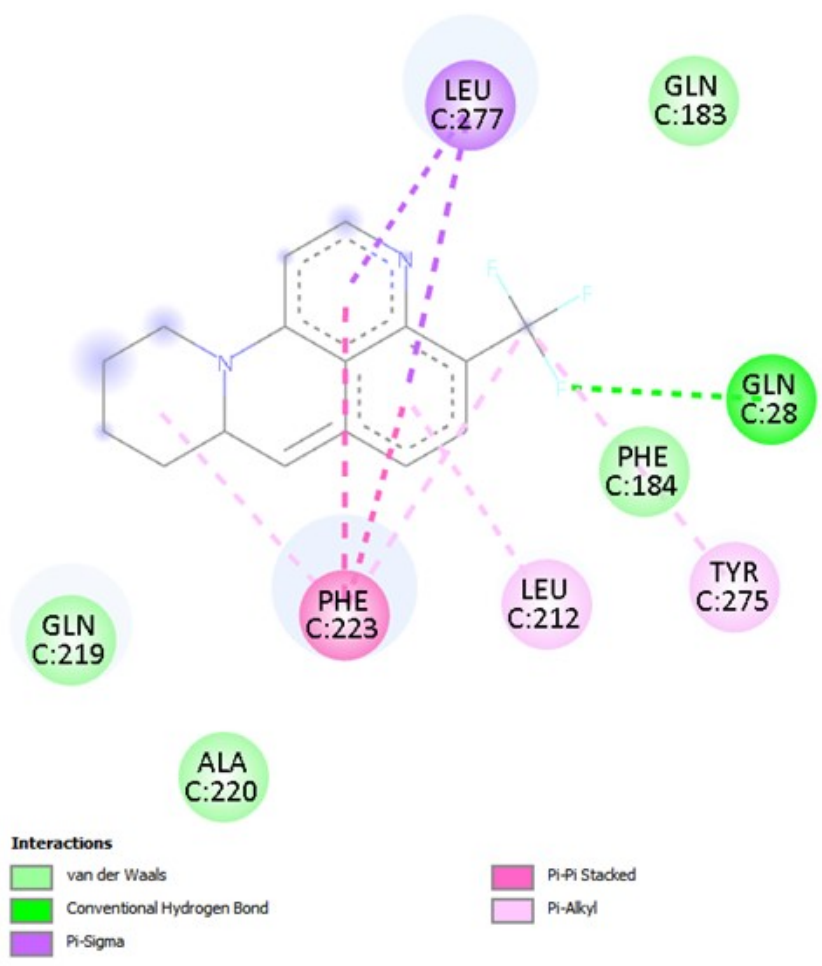

(**g**) 1ZP4-11 complex

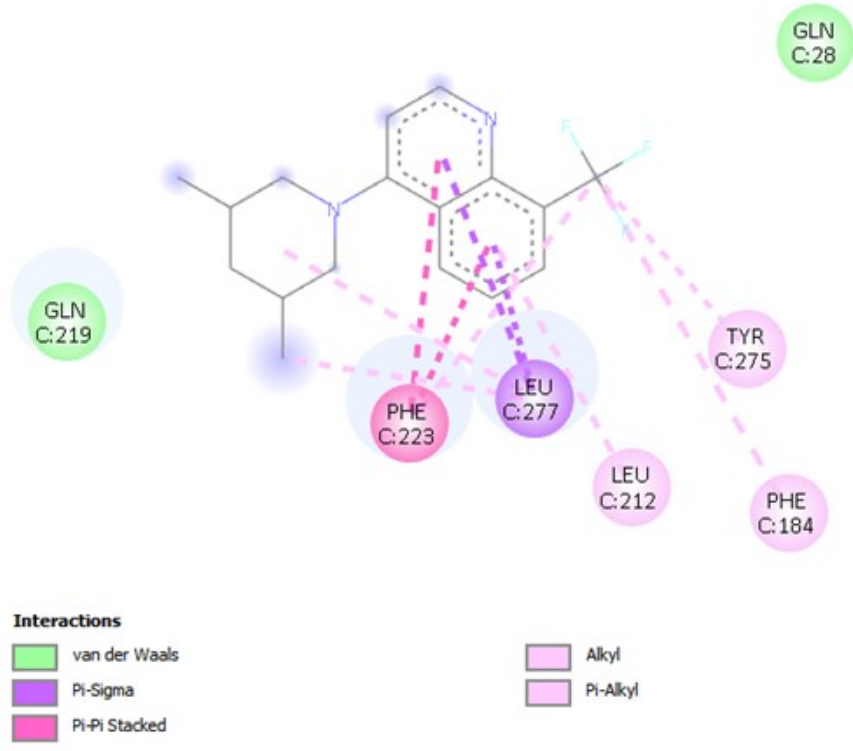

(**h**) 1ZP4-12 complex

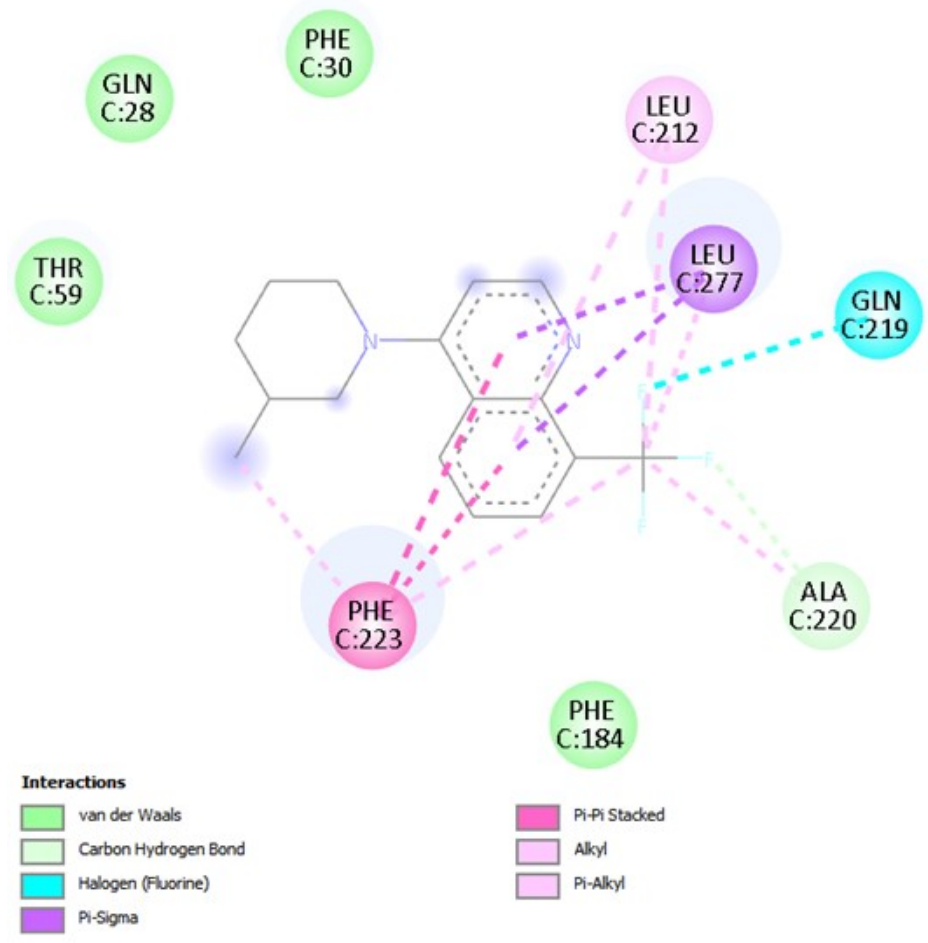

(i) 1ZP4-7 complex

Figure S25. 2D ligand-protein interaction of 1ZP4-complexes.

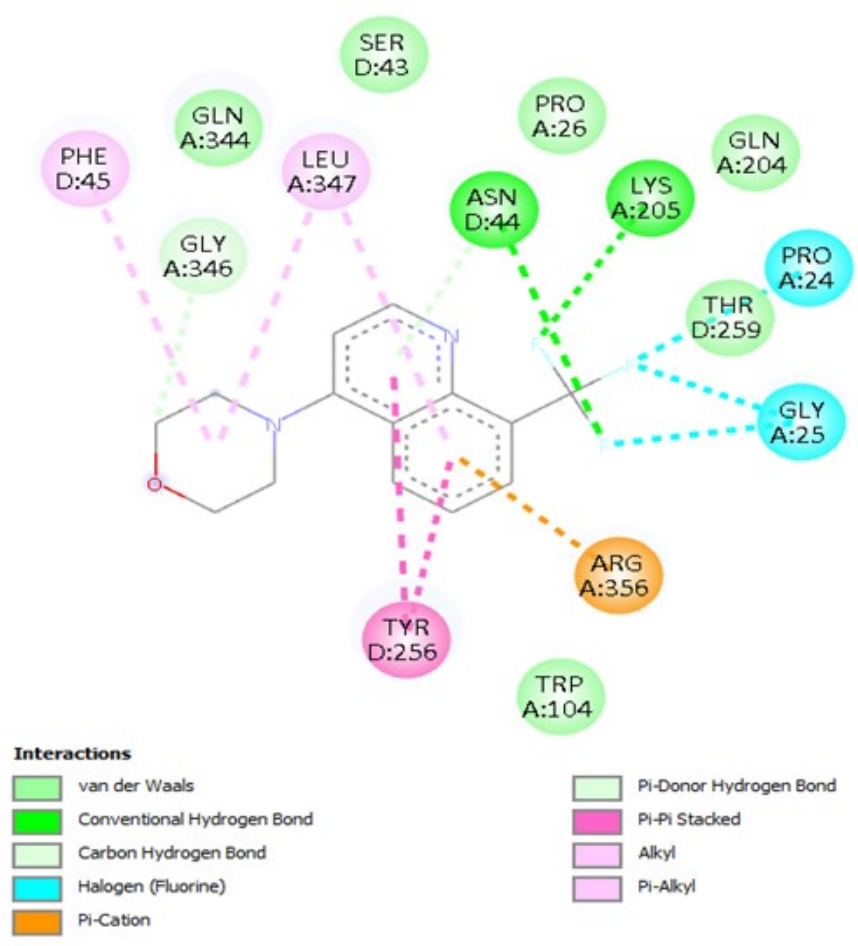

(a) 2CH2-5

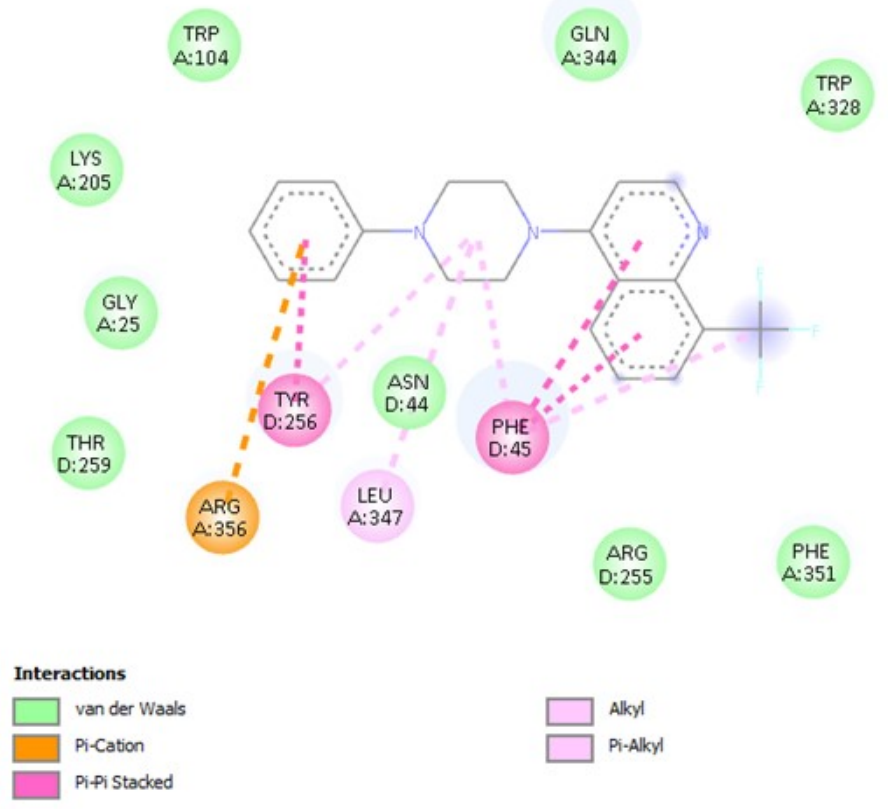

(b) 2CH2-6

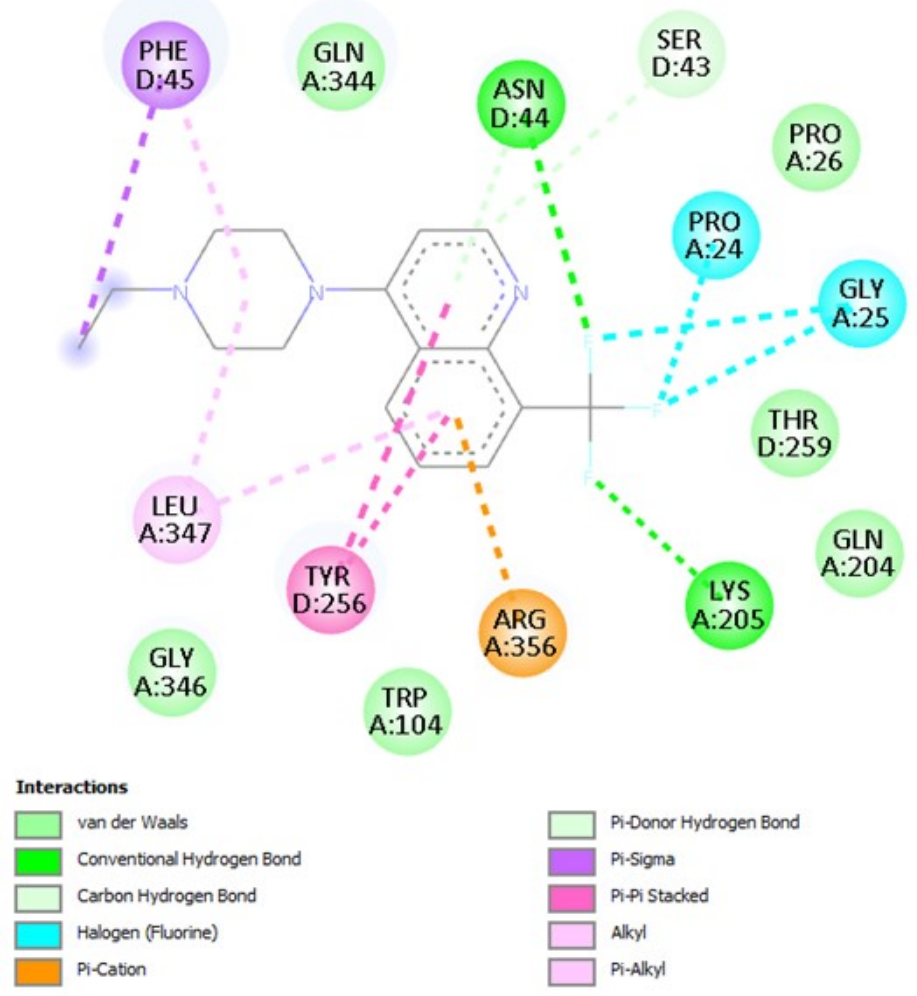

(c) 2CH2-7

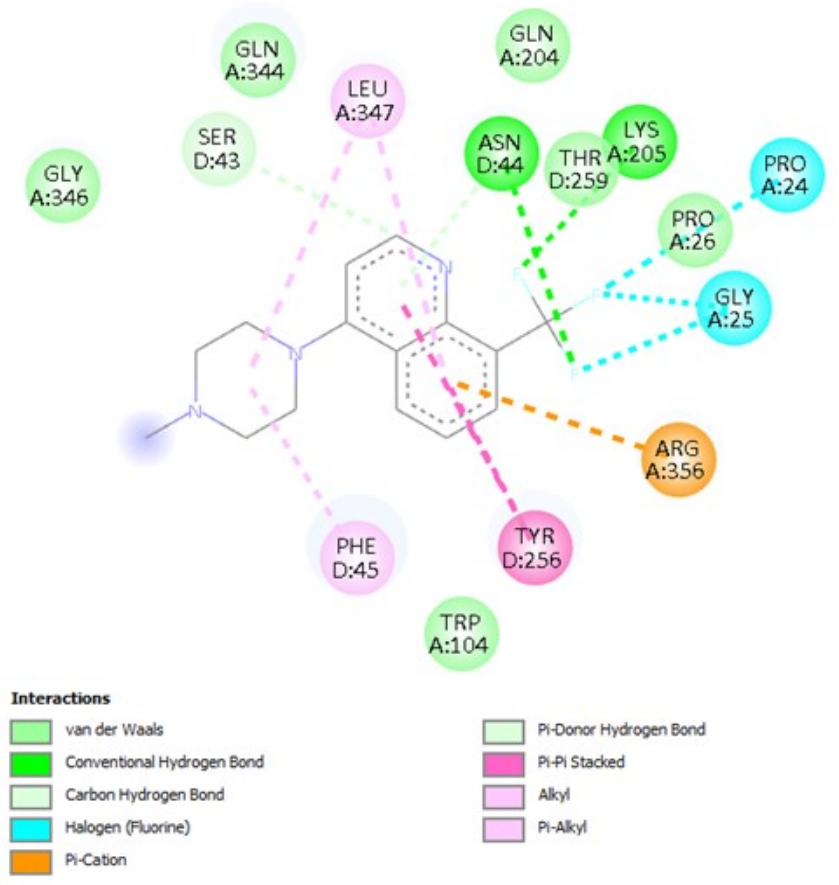

(d) 2CH2-8

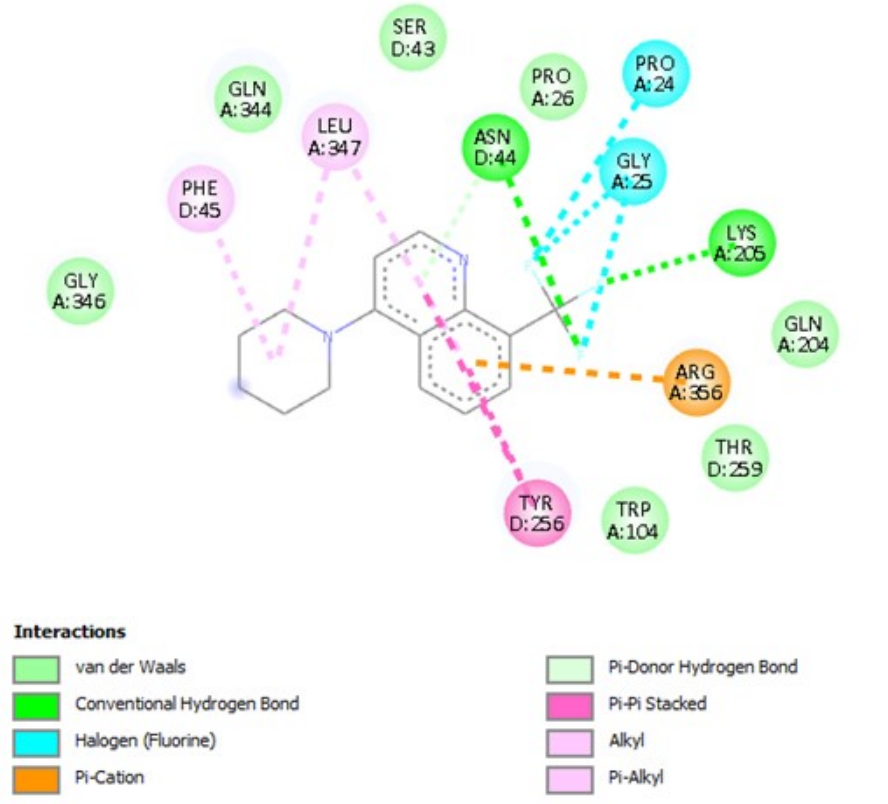

(e) 2CH2-9

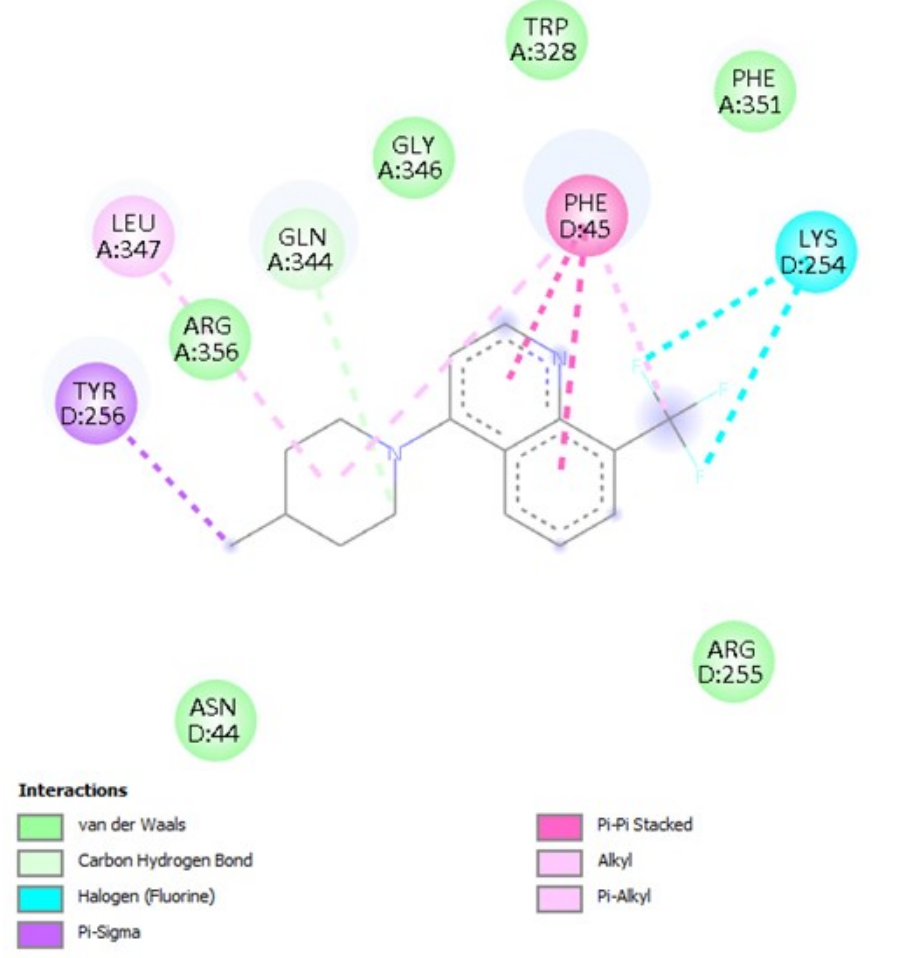

(f) 2CH2-10

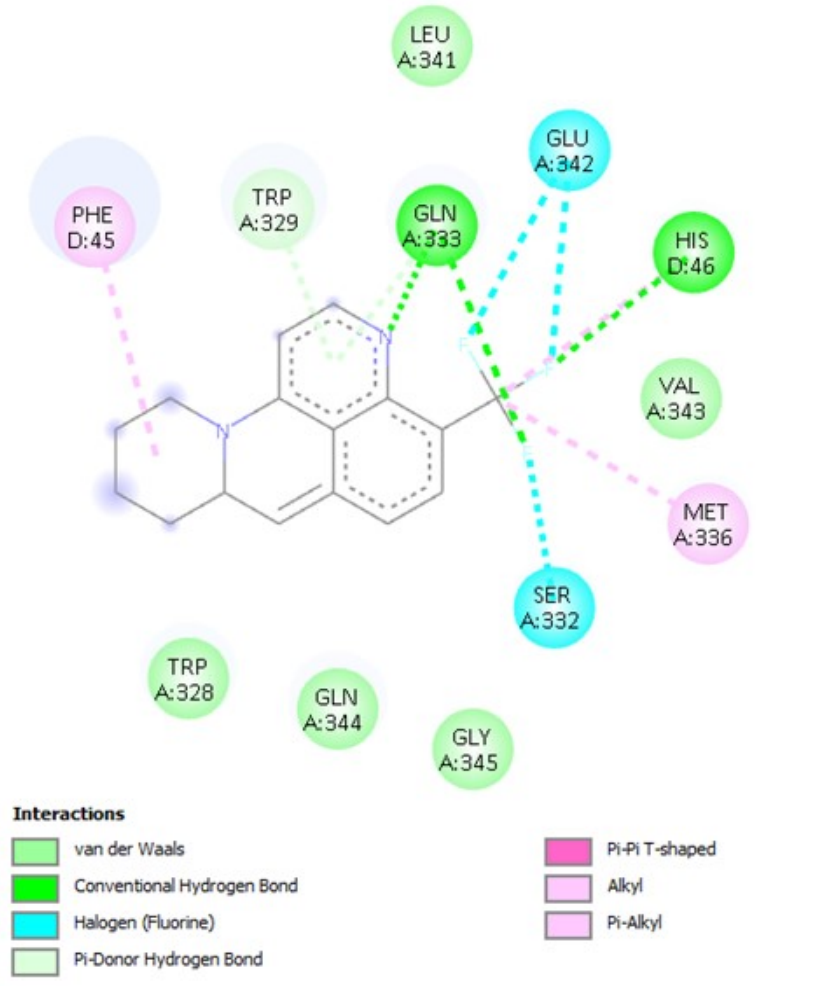

(g) 2CH2-11

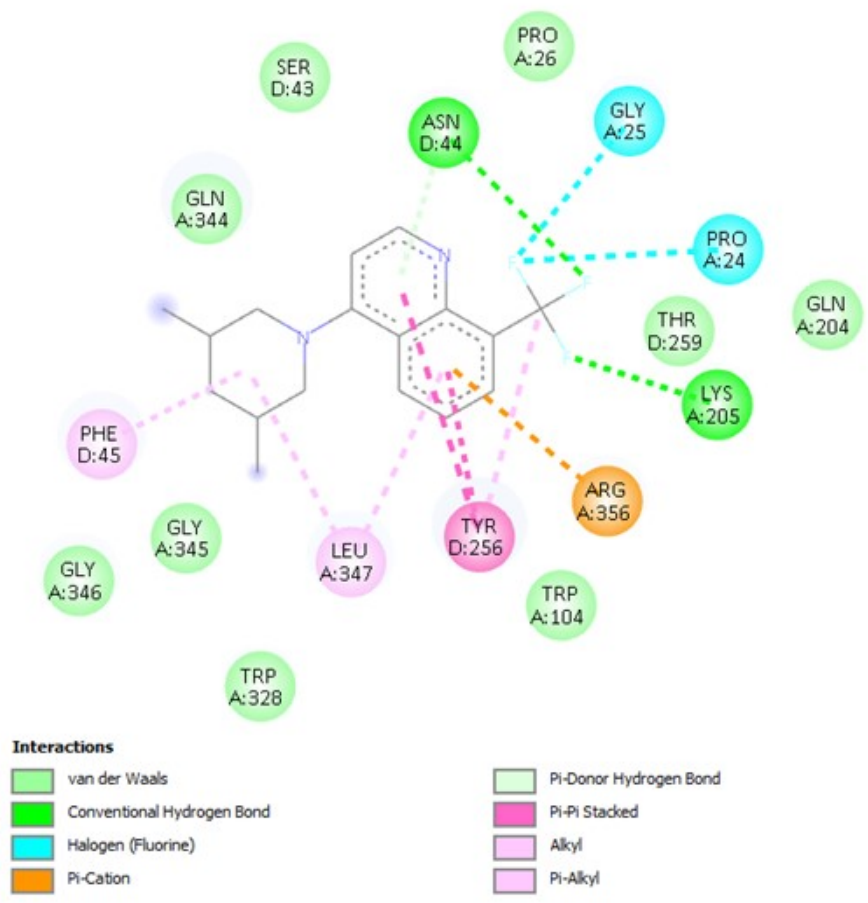

(h) 2CH2-12

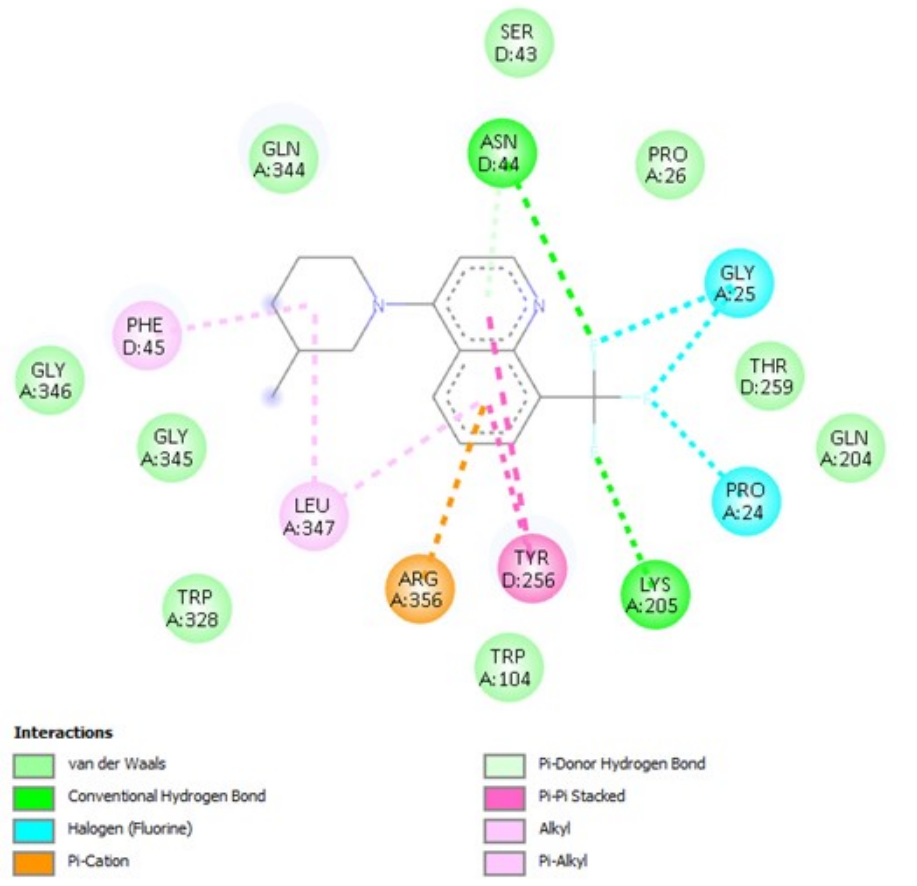

(i) 2CH2-13

Figure S26. 2D ligand-protein interaction of 2CH2-complexes.

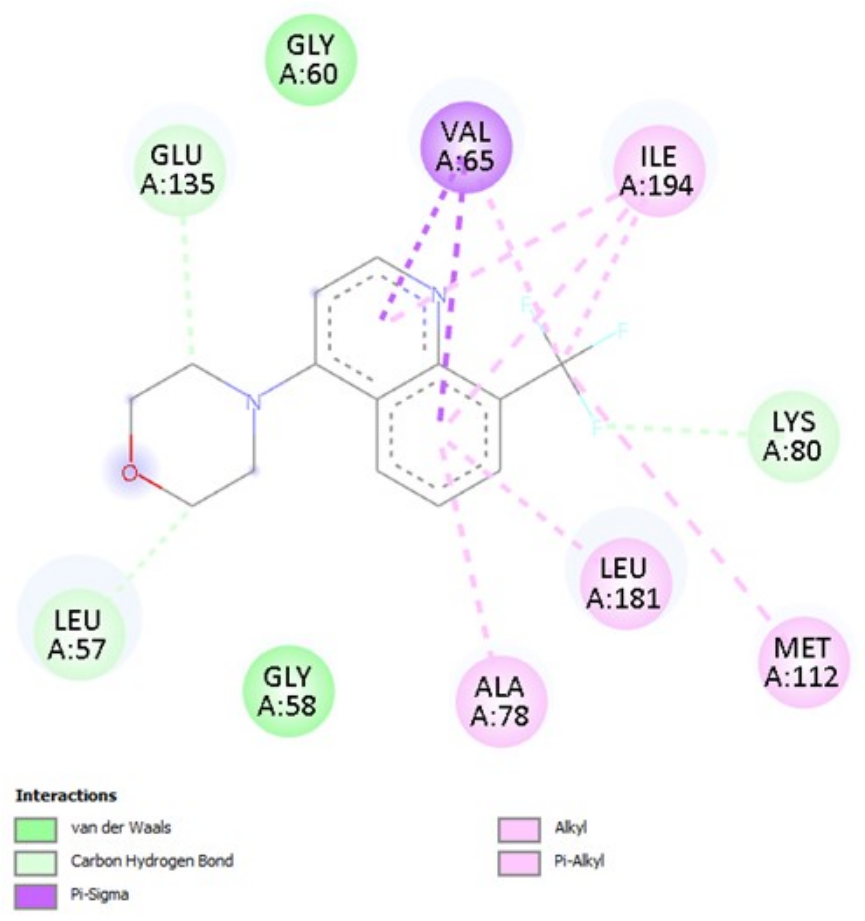

(a) 4JBV-5

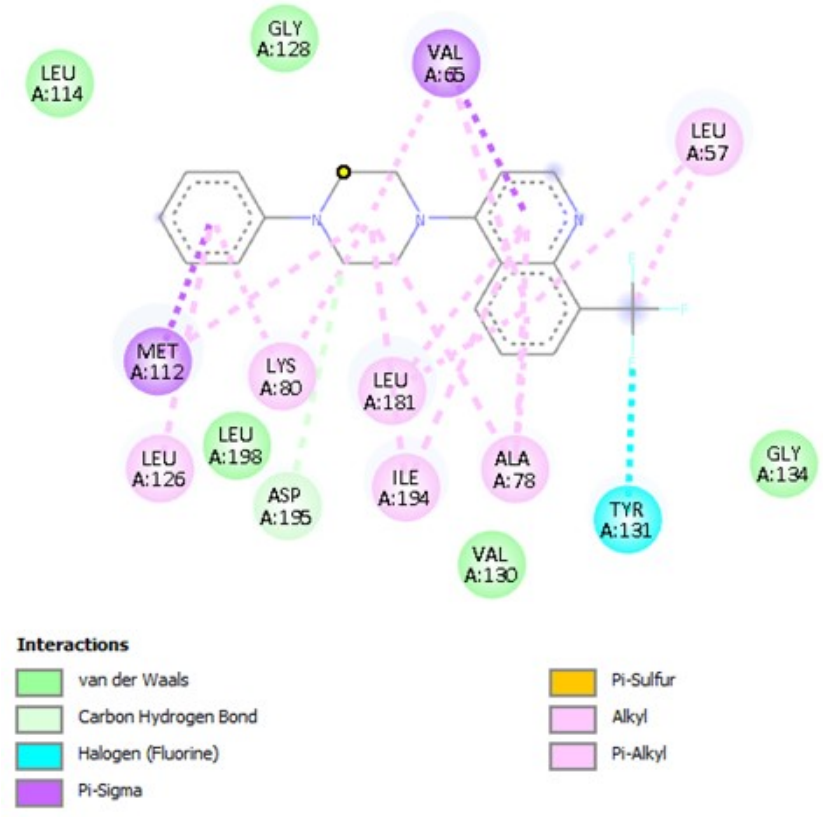

(b) 4JBV-6

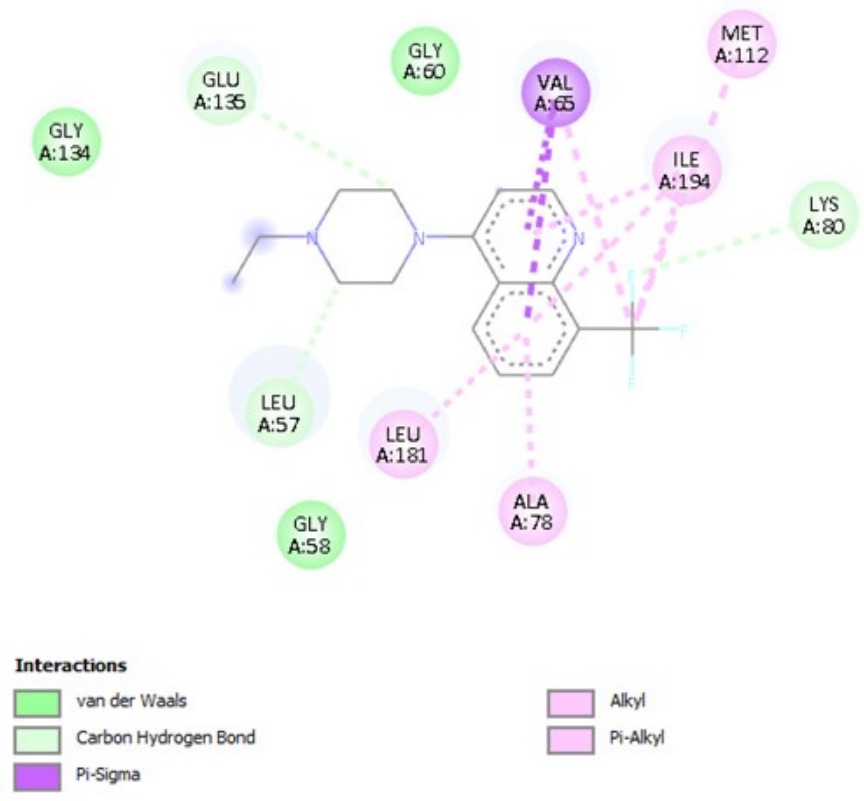

(c) 4JBV-7

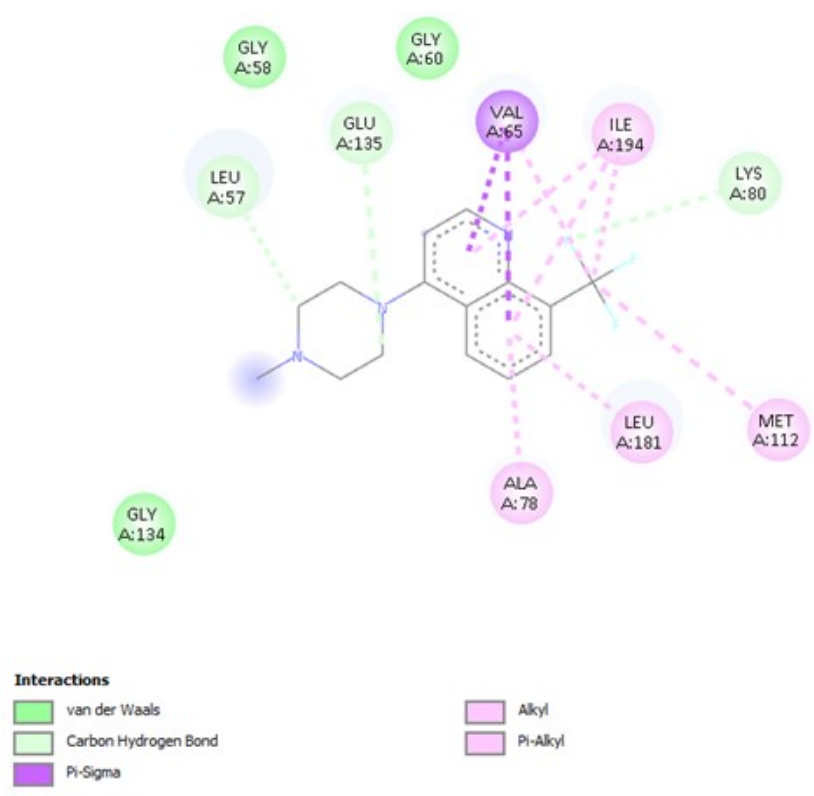

(d) 4JBV-8

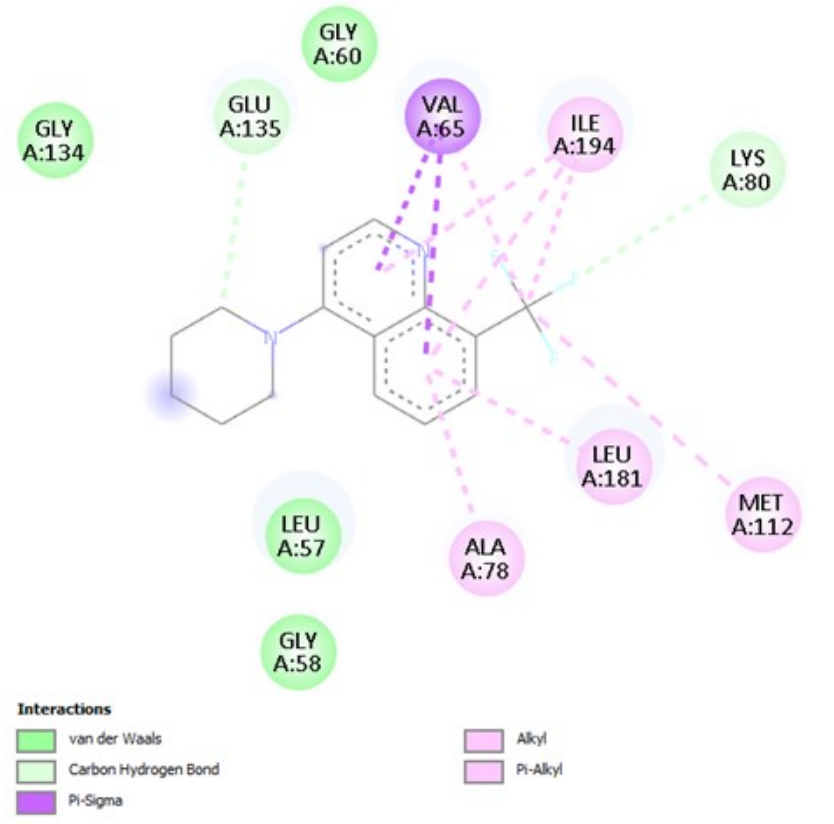

(e) 4JBV-9

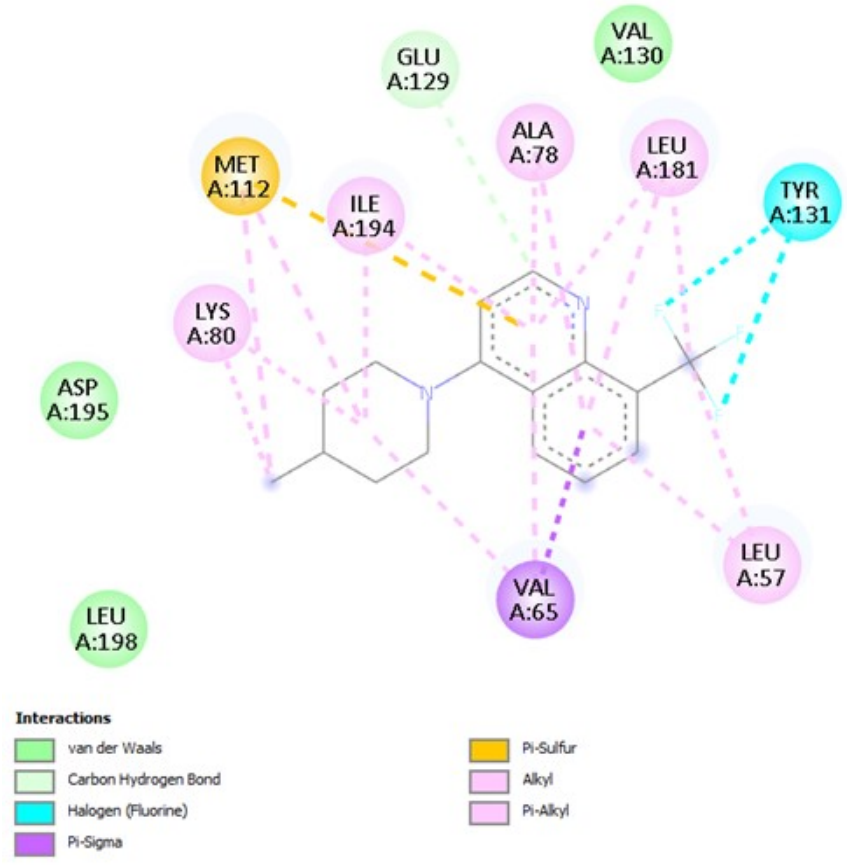

(f) 4JBV-10

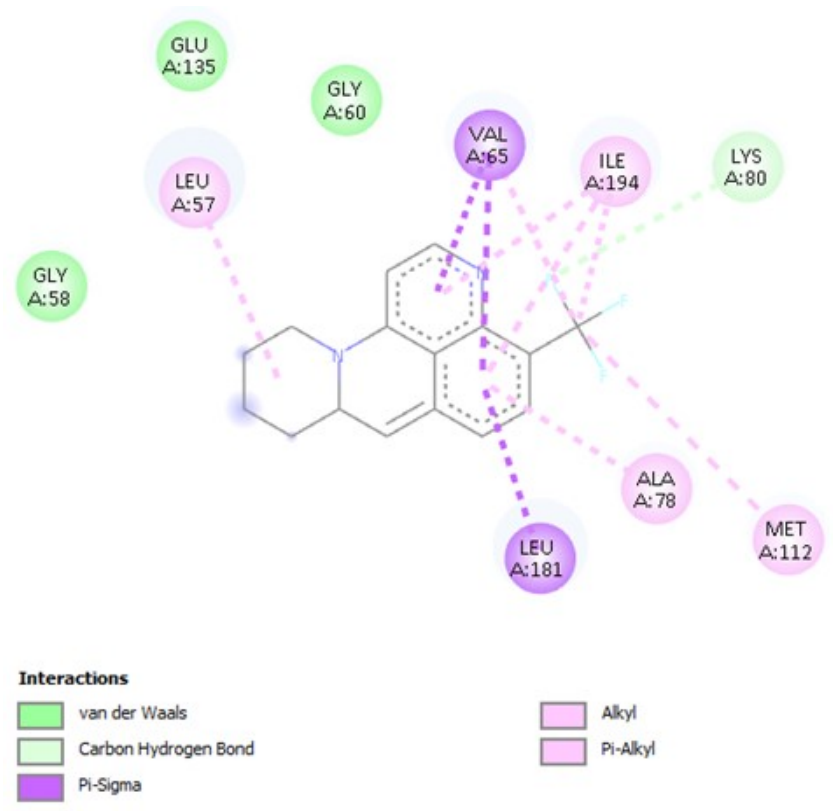

(g) 4JBV-11

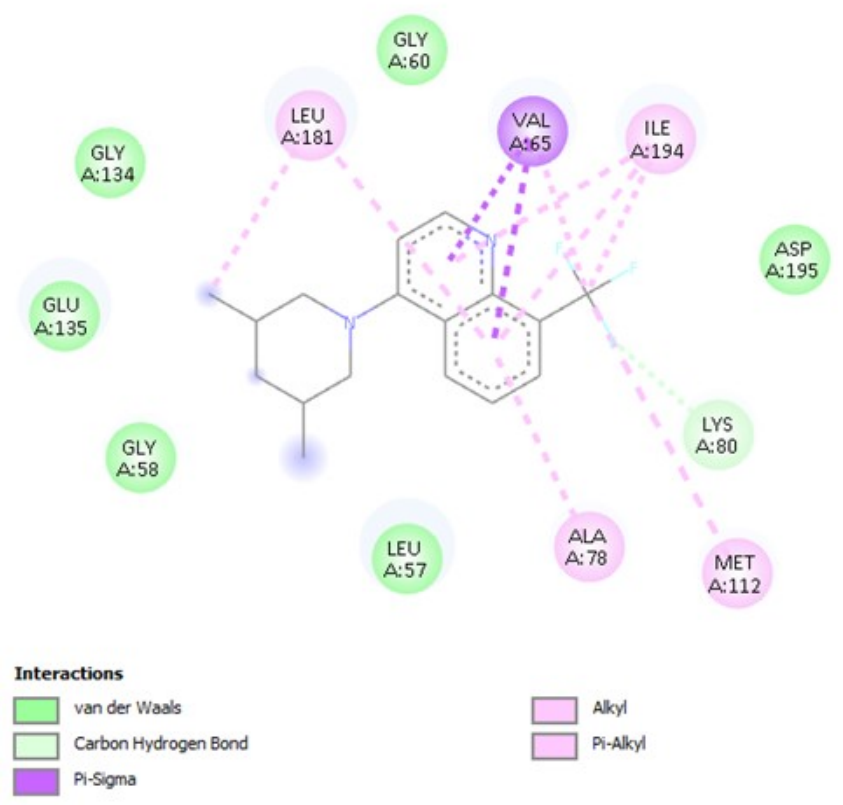

(h) 4JBV-12

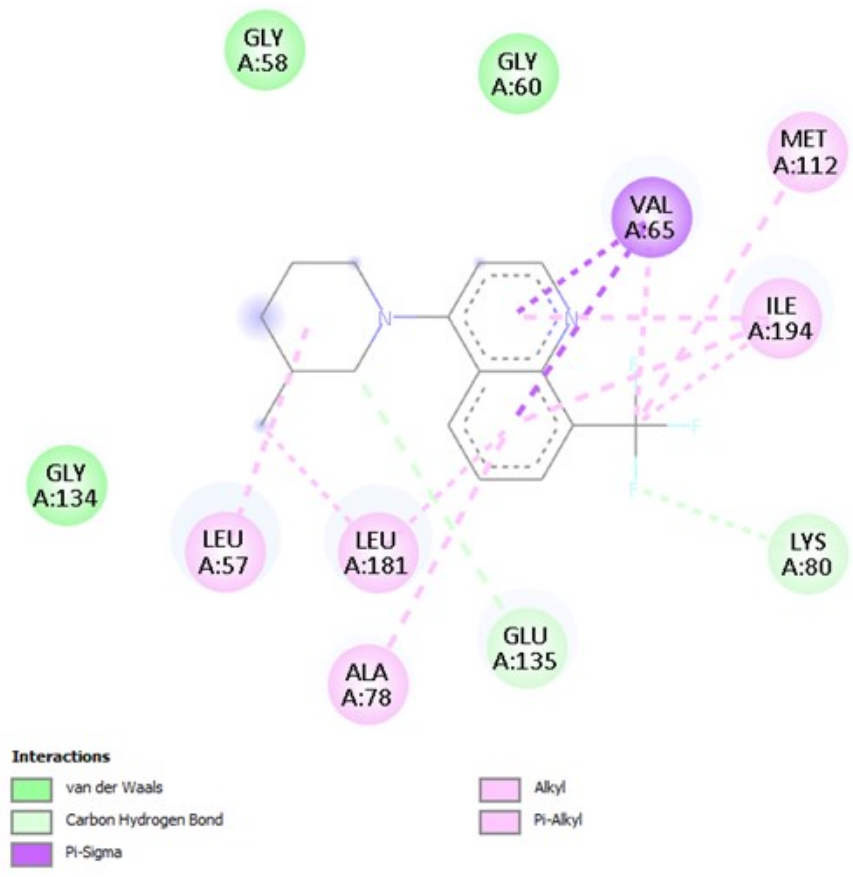

(i) 4JBV-13

Figure S27. Figure 2D ligand-protein interaction of 4JBV complexes.

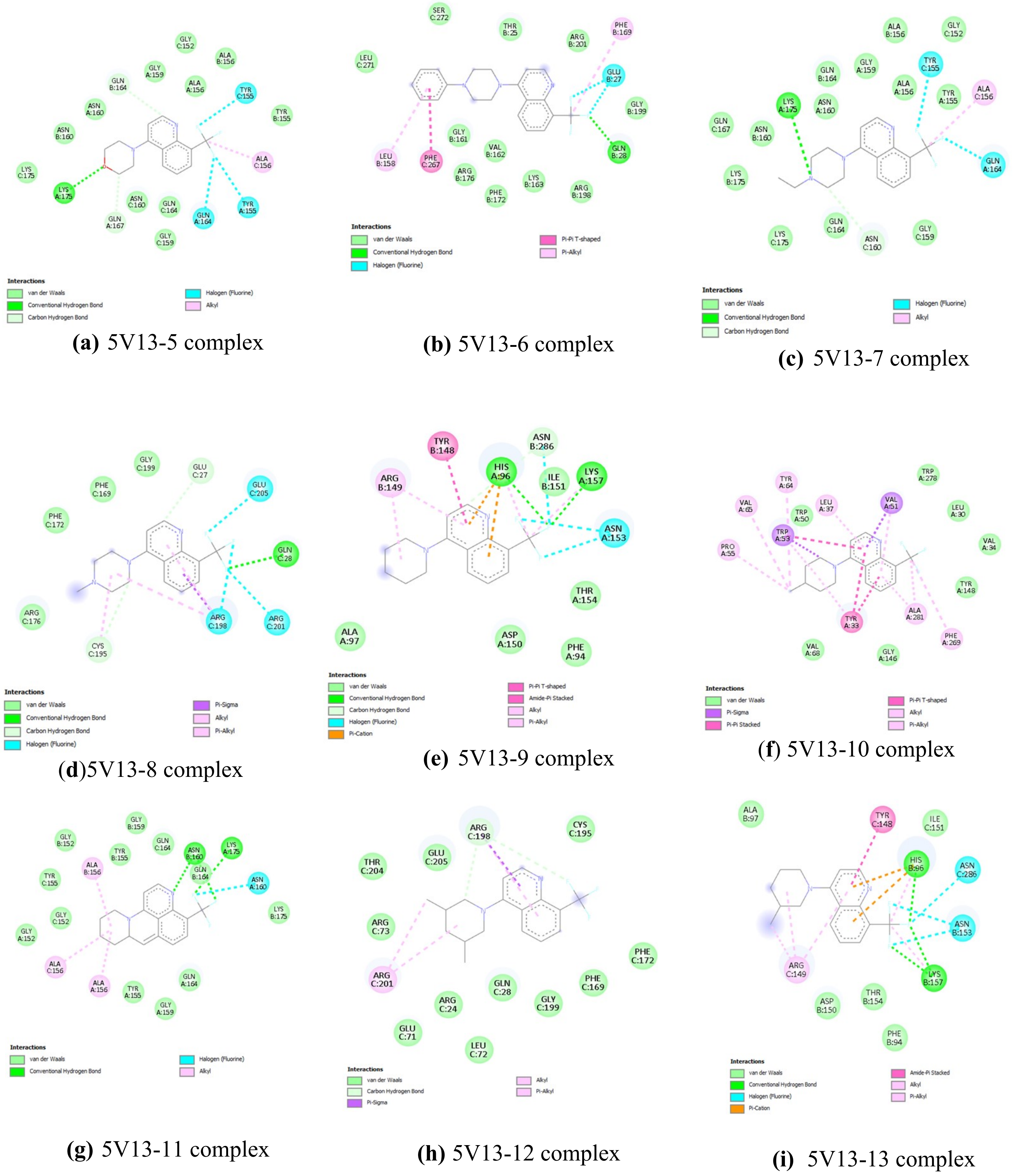

(a) 5V13-5 complex
(b) 5V13-6 complex
(c) 5V13-7 complex
(d) 5V13-8 complex
(e) 5V13-9 complex
(f) 5V13-10 complex
(g) 5V13-11 complex
(h) 5V13-12 complex
(i) 5V13-13 complex

Figure S28. 2D ligand-protein interaction of 5V13-complexes.

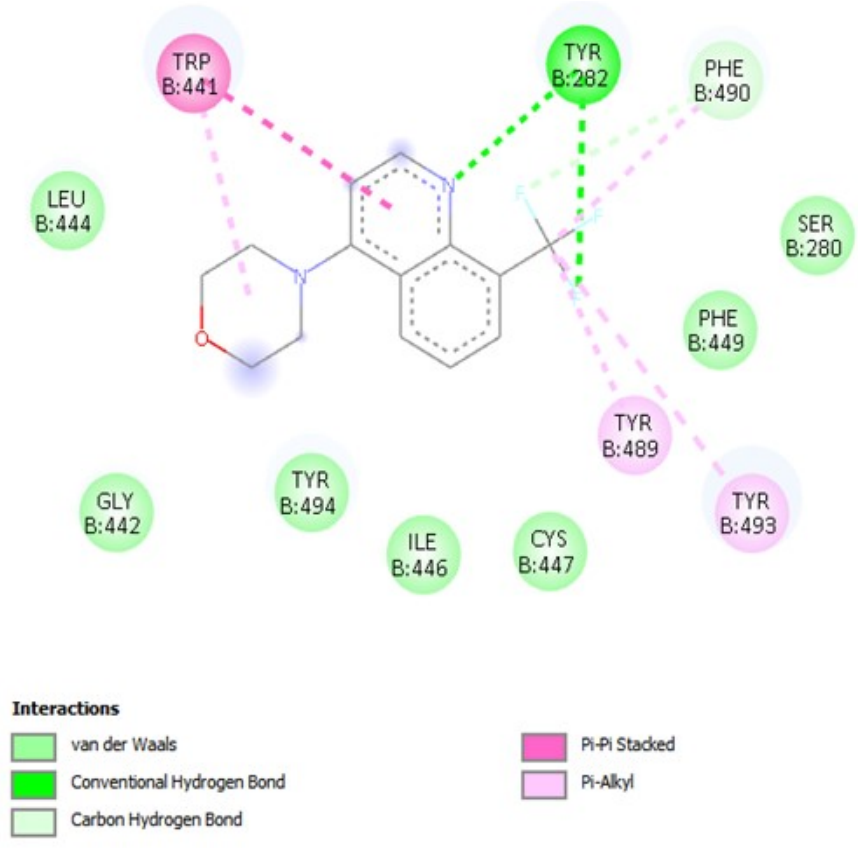

(a) 6ARY-5 complex

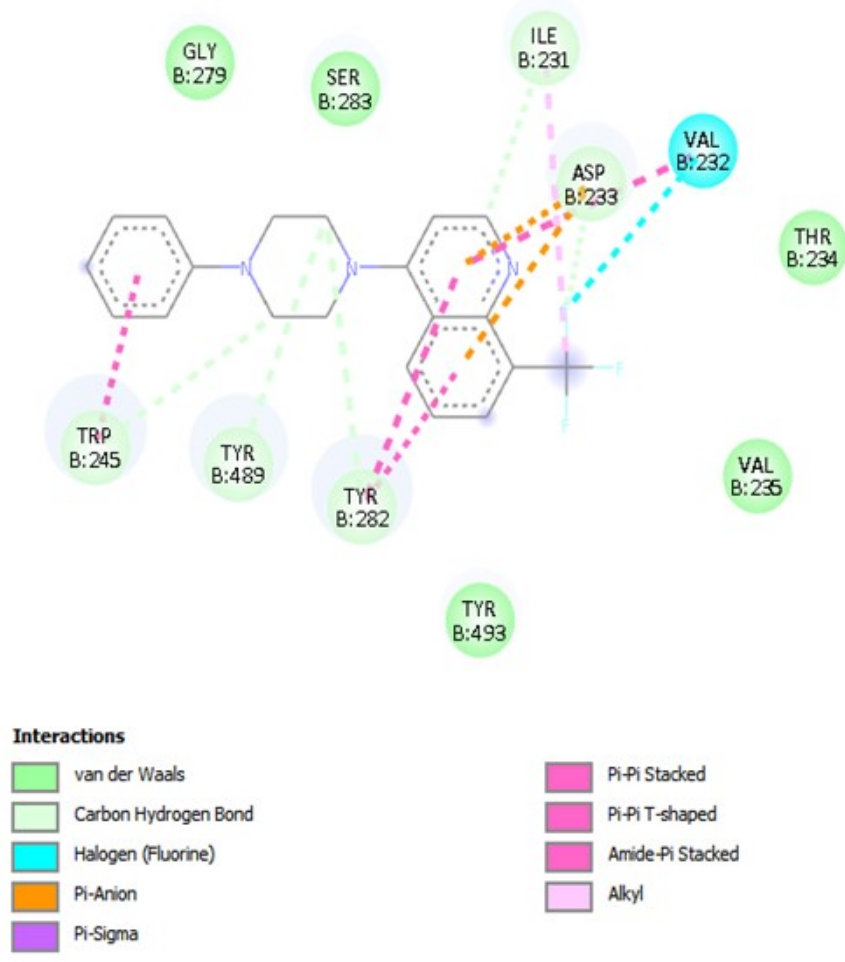

(b) 6ARY-6 complex

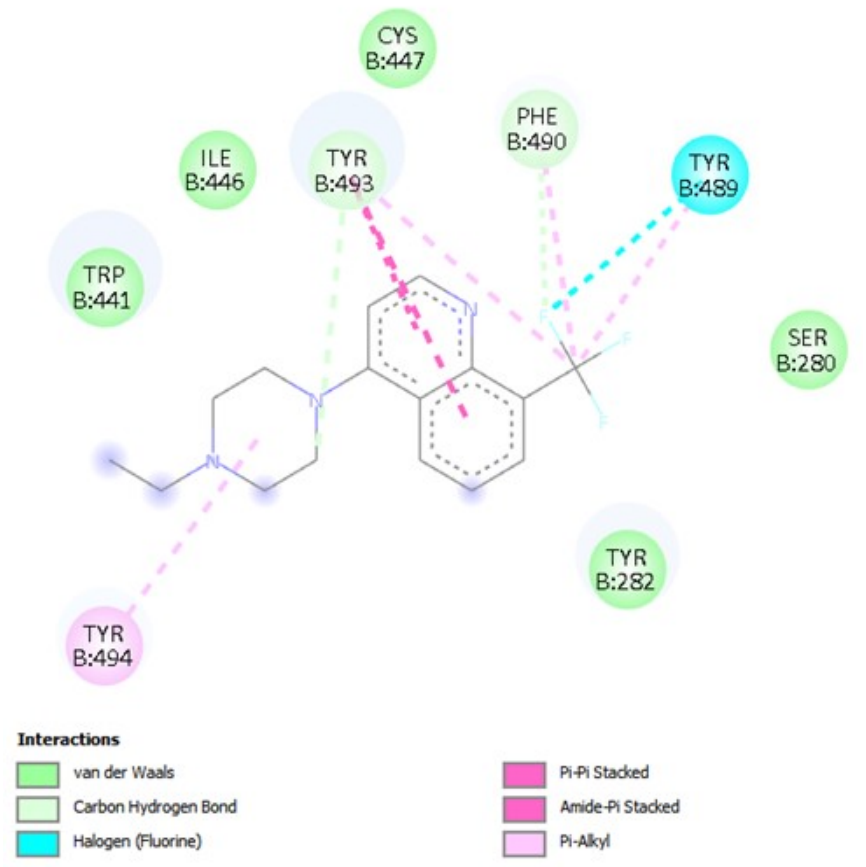

(c) 6ARY-7 complex

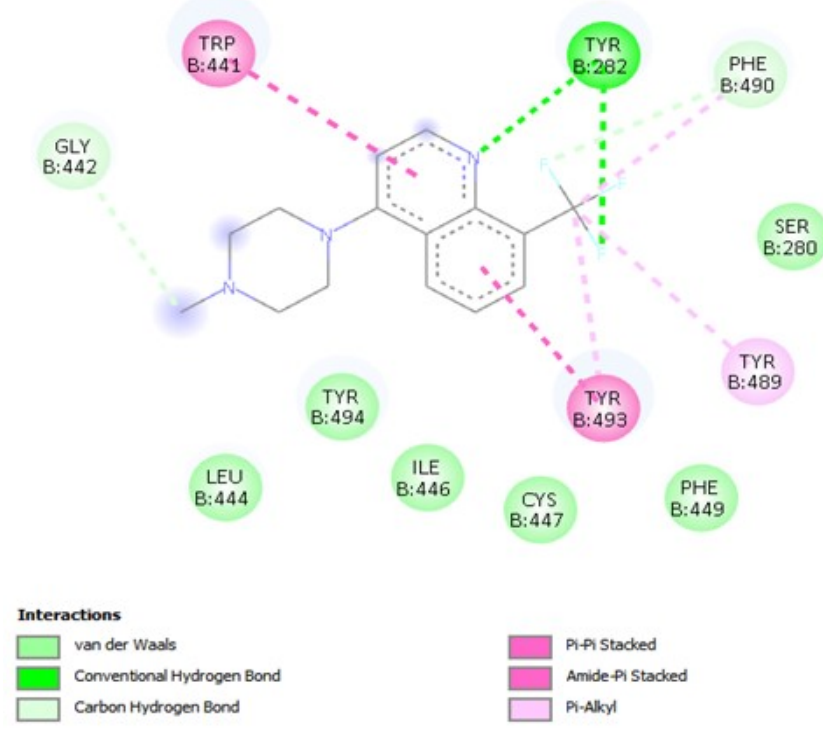

(d) 6ARY-8 complex

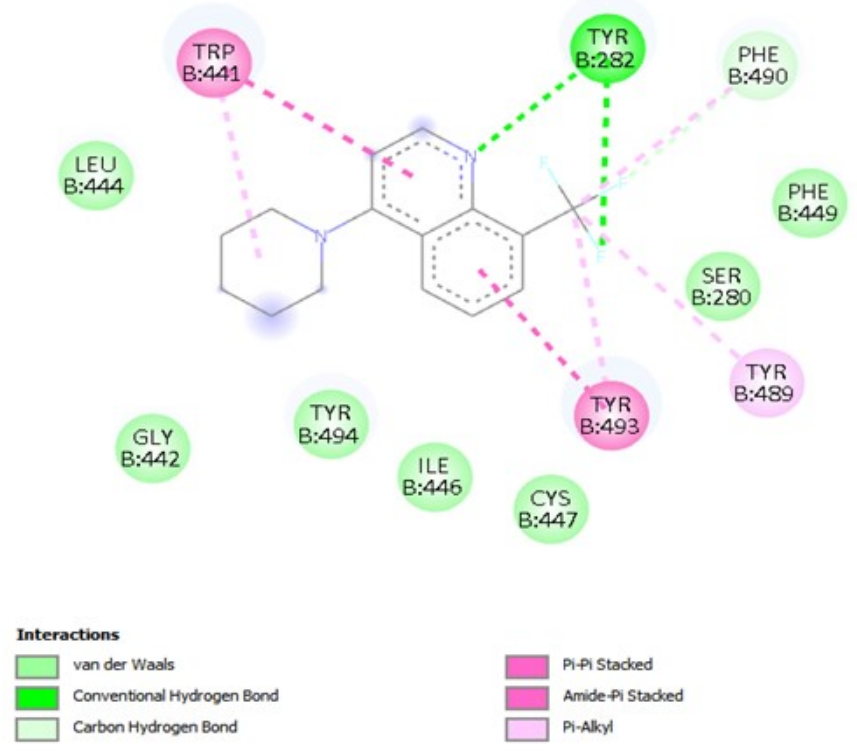

(e) 6ARY-9 complex

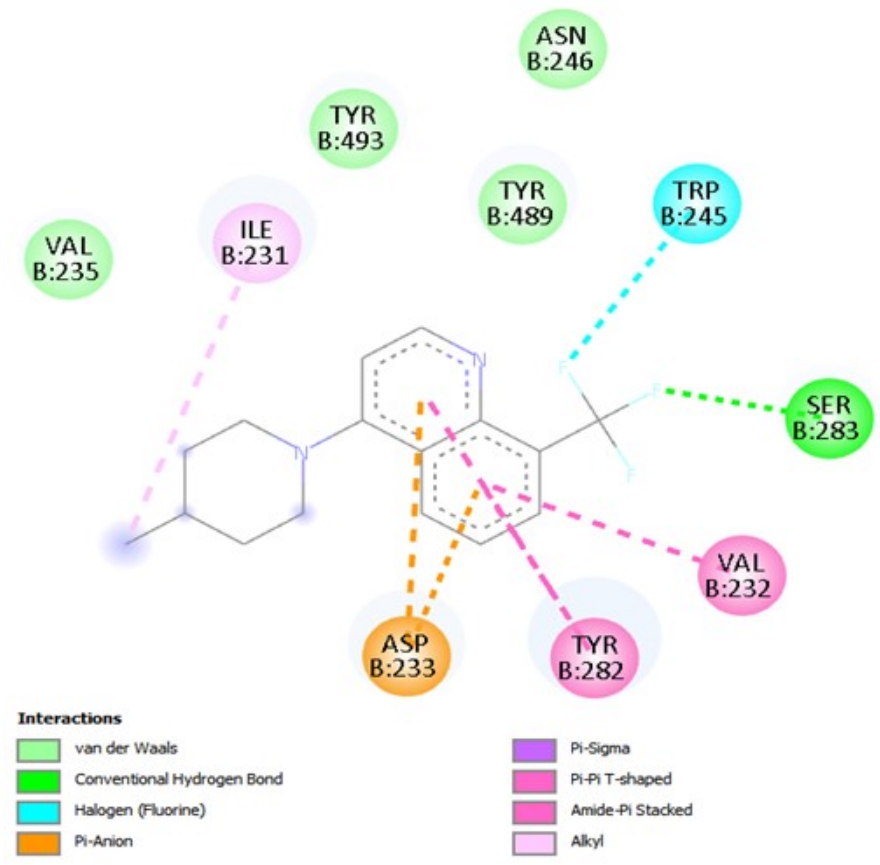

(f) 6ARY-10 complex

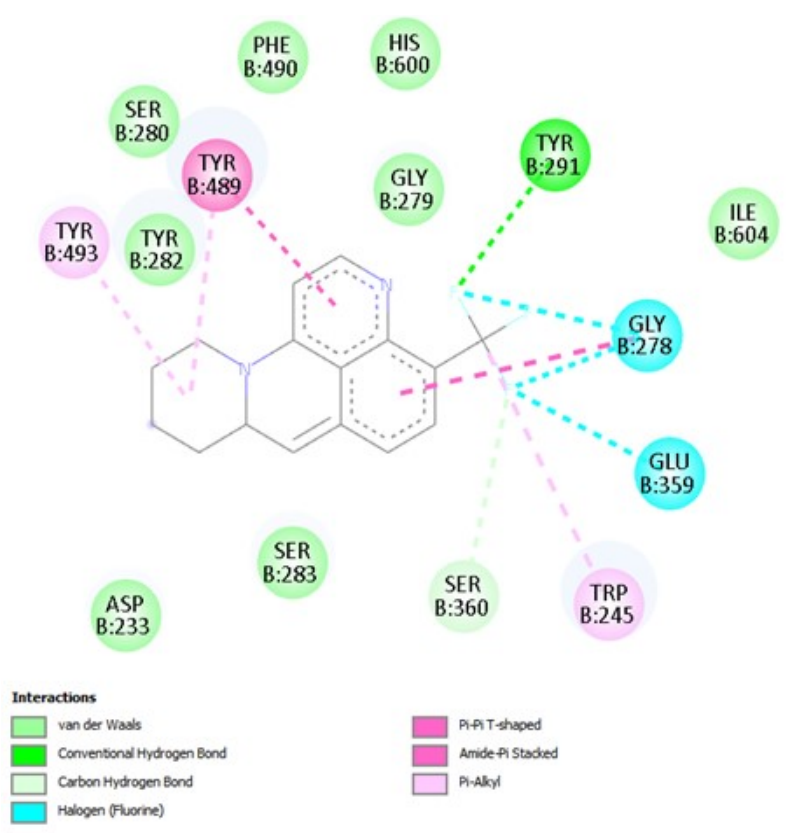

(g) 6ARY-11 complex

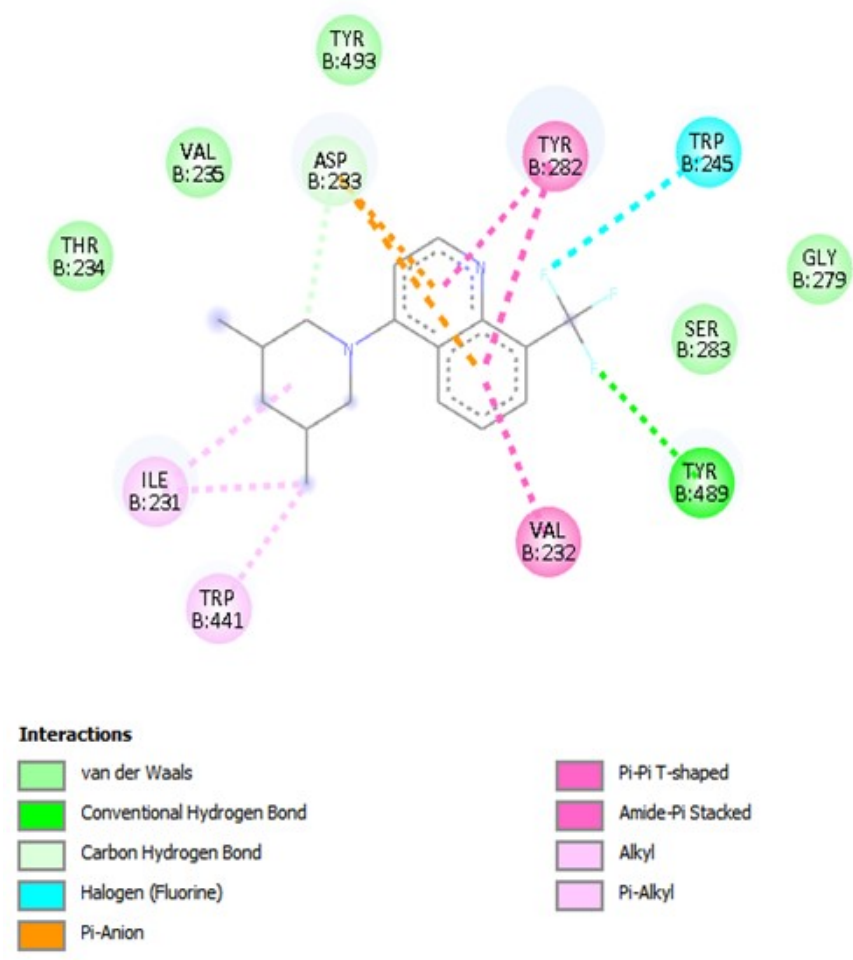

(**h**) 6ARY-5 complex

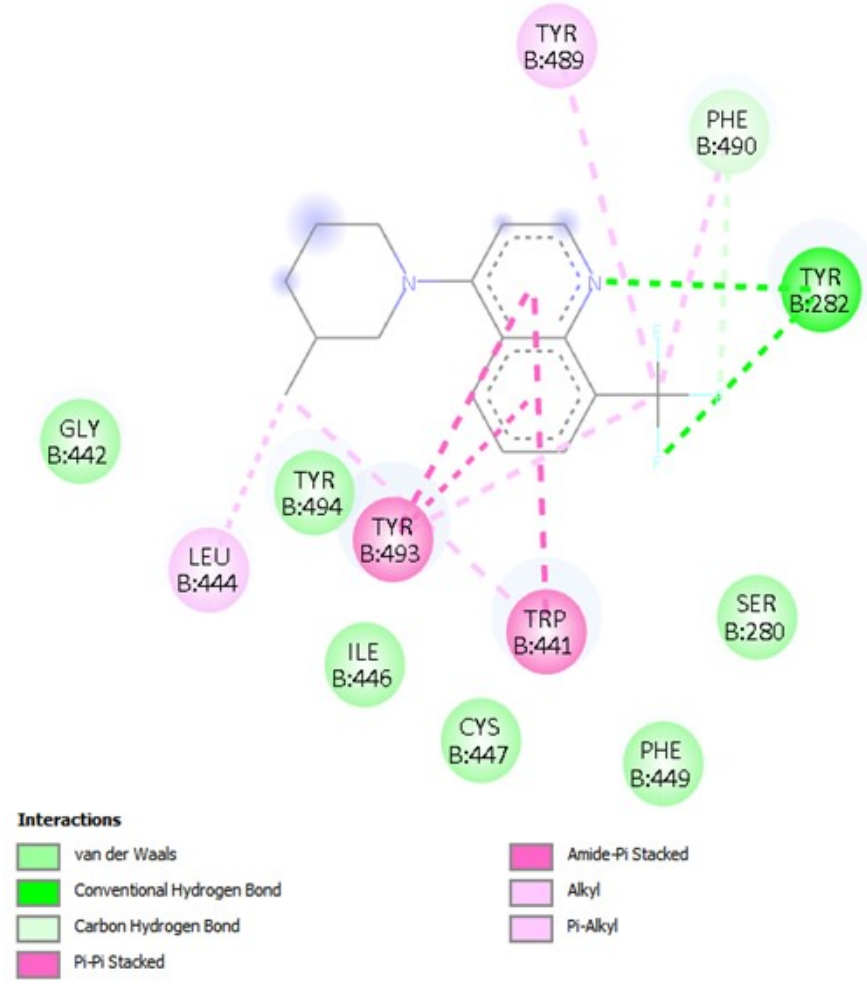

(j) 6ARY-5 complex

Figure S29. Figure 2D ligand-protein interaction of 6ARY-complexes.

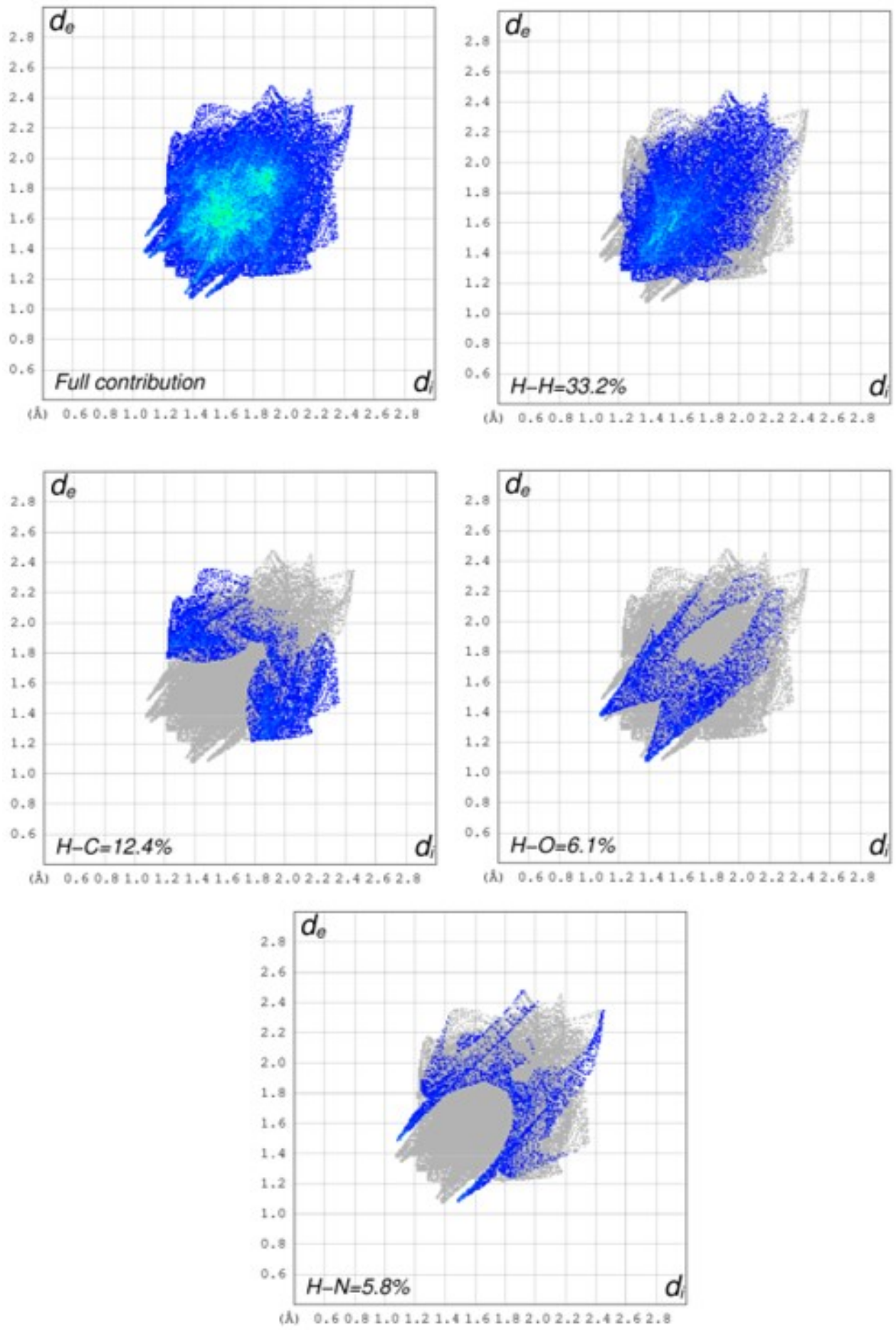

Figure S30. 2D fingerprint plot of compound **4a**.

Table S11. Molecular interaction energies (k J/mol) in compound **4a.**

	N	Symop	R	Electron Density	E_ele	E_pol	E_dis	E_rep	E_tot
	2	x, y, z	11.52	B3LYP/6-31G(d,p)	-1.2	-0.8	-6.7	1.1	-7.0
	1	-x, -y, -z	13.49	B3LYP/6-31G(d,p)	-4.0	-0.5	-5.6	1.4	-8.7
	2	x+1/2, -y+1/2, z+1/2	6.75	B3LYP/6-31G(d,p)	-0.5	-2.3	-31.7	15.1	-20.5
	2	-x+1/2, y+1/2, -z+1/2	9.88	B3LYP/6-31G(d,p)	-6.1	-1.8	-13.3	9.3	-13.6
	1	-x, -y, -z	7.05	B3LYP/6-31G(d,p)	-4.8	-1.6	-22.8	12.5	-18.4
	1	-x, -y, -z	4.20	B3LYP/6-31G(d,p)	-11.6	-3.2	-73.4	33.5	-57.9
	2	x+1/2, -y+1/2, z+1/2	9.63	B3LYP/6-31G(d,p)	-8.4	-2.4	-13.2	10.4	-15.7
	2	-x+1/2, y+1/2, -z+1/2	7.14	B3LYP/6-31G(d,p)	-9.6	-1.8	-20.6	9.8	-23.3

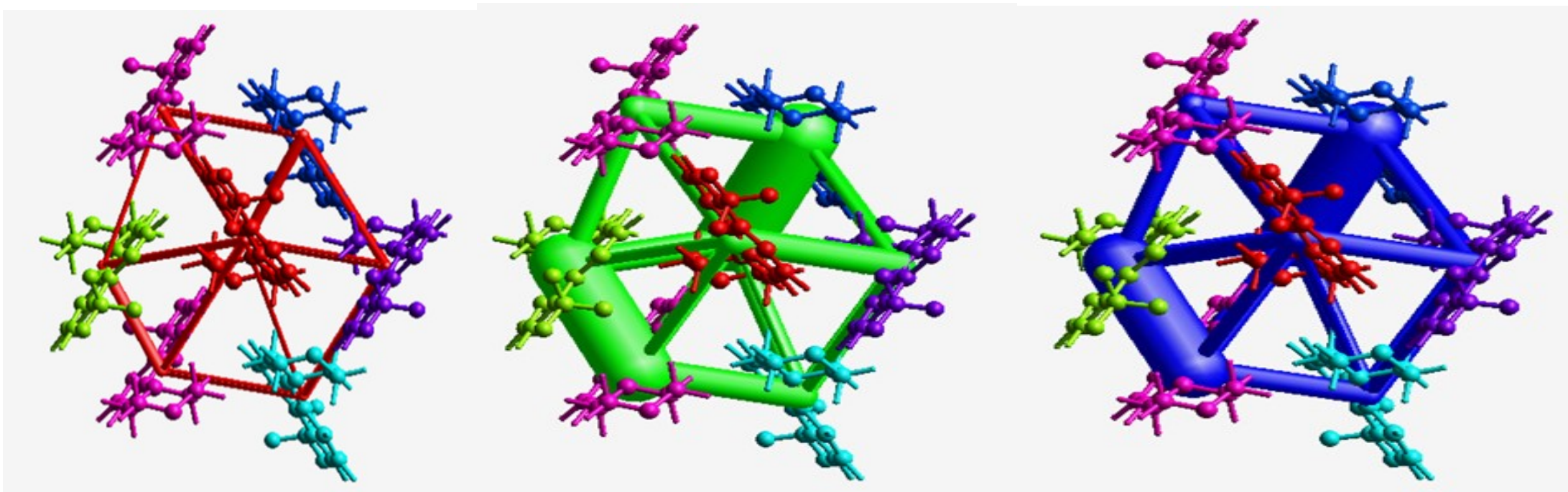

Figure S31. Graphical visualization of the **(a)** coulomb energy term (E_{ele}), **(b)** dispersion energy term (E_{dis}) and, **(c)** total energy term (E_{tot}) of compound **4a.**
